# Sexually transmitted infection prevalence and testing coverage among people who inject drugs: A systematic matic review

**DOI:** 10.1016/j.drugalcdep.2025.112732

**Published:** 2025-05-24

**Authors:** Olivia Price, Paige Webb, Jason Grebely, Amy Peacock, Nicholas Medland, Phillip Read, Emily Cooke, Matthew Hickman, Peter Vickerman, Louisa Degenhardt

**Affiliations:** a National Drug and Alcohol Research Centre, UNSW, Sydney, Australia; b Kirby Institute, UNSW, Sydney, Australia; c School of Psychological Sciences, University of Tasmania, Hobart, Australia; d Kirketon Road Centre, South Eastern Sydney Local Health District, Sydney, NSW, Australia; e Population Health Science, Bristol Medical School, University of Bristol, Bristol, UK

**Keywords:** Sexually transmitted infections, People who inject drugs, Syphilis, Chlamydia, Gonorrhoea

## Abstract

**Background:**

Individual- and structural-level factors place people who inject drugs at excess risk of sexually transmitted infections (STI). We reviewed existing evidence on STI prevalence and testing coverage among this population.

**Methods:**

We conducted searches of peer-reviewed and grey literature for papers or reports published between January 2008 and April 2024. Data were summarised using narrative synthesis, and where appropriate, regional and global estimates weighted by population size of people who inject drugs were generated using random effects meta-analysis. Meta-regressions were undertaken to examine study- and country-level sources of heterogeneity.

**Findings:**

Data on STI prevalence and testing coverage were sparse, which precluded meta-analysis except for syphilis prevalence. We estimated global active syphilis prevalence among people who inject drugs as 3.2 % (95 % confidence interval [CI]: 2.3–4.6). There was regional variation, with estimates ranging from 0.1 % (0–0.3) in North America to 7.2 % (3.6–11.7) in Latin America. Based on a small number of studies (n = 12) that stratified estimates by gender, active syphilis prevalence was higher among women (8.1 %, 3.6–13.7) than men (2.3 %, 1.4–3.5). Higher HIV prevalence among people who inject drugs at both study and country level were associated with higher syphilis prevalence. Chlamydia prevalence estimates (n = 22) ranged 0–12.5 %, gonorrhoea prevalence (n = 20) ranged 0–2 %, and past year STI testing uptake (n = 8) ranged 8–62 %.

**Interpretation:**

More evidence on STI prevalence and testing uptake among people who inject drugs is urgently needed. Embedding STI diagnosis and treatment services within bloodborne virus programs could be an effective control measure.

## 1. Introduction

Globally, sexually transmitted infections (STI) pose a significant pubic health challenge (Rowley et al., 2019). Although STIs are often asymptomatic, when left untreated they are associated with negative health outcomes, including cancer, infertility, stillbirth, neurological and cardiovascular disease, and increased risk of HIV transmission (Galvin and Cohen, 2004; Holmes et al., 2008). STIs remain stigmatised, which creates barriers to testing and treatment (Hood and Friedman, 2011).

People who inject drugs appear to be at elevated risk of STIs (Bradshaw et al., 2005; Brookmeyer et al., 2019; Khan et al., 2013; Kidd et al., 2019; Reno et al., 2020). Substance use (regardless of route of administration) can interfere with decision-making and increase likelihood of engaging in sexual risk behaviours (e.g., unprotected sex) (Leigh and Stall, 1993). Stimulant use, particularly methamphetamine, is associated with increased impulsivity and altered sexual behaviour (Strathdee et al., 2021). Indirectly, marginalisation, socioeconomic and other structural factors increase reliance on sex work or transactional sex among people who inject drugs (Dumchev, 2022). Indeed, globally it is estimated that 14.9 % of people who inject drugs have recently engaged in sex work (Degenhardt et al., 2023). People who inject drugs also experience barriers to primary care (Motavalli et al., 2021). which may extend to STI testing and treatment and compound the increased transmission risk.

Intersectionality is an important consideration when examining the epidemiology of STIs among people who inject drugs. For example, transactional sex, sex work, and the experience of gender-based violence appear to be more common among women who inject drugs (Dumchev, 2022; Iversen et al., 2021; Valencia et al., 2020). and these risk environments may increase STI risk. The intentional use or injection of substances (particularly methamphetamine) to enhance or disinhibit sexual experiences is more common among gay, bisexual and other men who have sex with men and people who engage in sex work (Compton and Jones, 2021; Iversen et al., 2021; Strathdee et al., 2021). Data on substance use and sexual risk among other gender or sexual minority groups (e.g., bisexual and other women who have sex with women, transgender people) is lacking (Hibbert et al., 2021). There is evidence to suggest they are at greater risk of hepatitis C and HIV infection (Iversen et al., 2015). although more research is needed to discern whether this is a result of sexual or injecting risk (German and Latkin, 2015; Iversen et al., 2015). A better understanding of which subpopulations experience a greater prevalence of disease would be useful to more efficiently direct interventions (e.g., testing campaigns).

While there is evidence that people who inject drugs are at greater risk of STIs, there has been no previous systematic review of existing evidence and global STI prevalence estimates for this population are not known (World Health Organization, 2022). To address these research gaps, we conducted a systematic review of peer-reviewed and grey literature to examine the STI prevalence and STI testing coverage among people who inject drugs.

## 2. Methods

Data were drawn from a global, multi-stage systematic review of peer-reviewed and grey literature primarily conducted to examine the sociodemographic characteristics and bloodborne virus prevalence among people who inject drugs, but also included variables relating to STIs (PROPSPERO registration: CRD42020173337; Degenhardt et al., 2023). The methodology adheres to PRISMA (Moher et al., 2009). and GATHER (Stevens et al., 2016) guidelines (Appendices A and B).

### 2.1. Eligibility criteria

We included studies that met the following criteria: (a) published January 1, 2008, onwards; (b) study population included people who inject drugs; and (c) reported the proportion of people who inject drugs with a sexually transmitted infection or who had been tested for a sexually transmitted infection.

We excluded data prior to 2008 because STI incidence has increased over time and these data may not be representative of the current situation. We defined people who inject drugs as those with a lifetime history of injecting drug use however defined by the study (e.g., self-report, physical evidence of injecting drugs). We also considered clients of supervised injecting centres and needle-syringe programs to be people who inject drugs.

We focused on non-bloodborne STIs because bloodborne STIs (e.g., HIV, hepatitis C virus) have been described extensively among people who inject drugs (Degenhardt et al., 2023). We included data on bacterial vaginosis, chlamydia, donovanosis, gonorrhoea, herpes simplex 2 virus, human papillomavirus, Lymphogranuloma venereum, syphilis, and trichomoniasis. We included estimates regardless of ascertainment method (e.g., self-report, diagnostic test conducted by the study investigators). For STI testing coverage estimates, we were interested in testing for any STI and only included testing conducted outside of the study.

Studies were excluded if the sample size was insufficient (<40), the study population was a subpopulation (e.g., participants recruited based on their HIV or other bloodborne virus status, incarceration history, or sexual identity). We also excluded the following study designs: case-control studies, non-original work (e.g., editorials, reviews), and non-baseline cohort studies.

There were no limitations on language. There were study team members proficient in English, French, Farsi and Mandarin; other languages were read via Google Translate or the Microsoft Word 365 translate function.

### 2.2. Information sources, search strategy and selection process

In the original review (Degenhardt et al., 2023), we conducted searches of the peer-reviewed literature (Medline, Embase, and PsycINFO) and key websites and grey literature (Tran et al., 2020). The search strategy combined search domains for injecting drug use, epidemiology research, bloodborne viruses, and harm reduction measures for people who use drugs. We also contacted experts at relevant international agencies for data. Multiple iterations of the search have been conducted since 2007 (Degenhardt et al., 2017, 2023; Mathers et al., 2008; Nelson et al., 2011). with the most recent search conducted on 1 April 2022. More detailed information, including the exact search strategy, is published elsewhere (Degenhardt et al., 2023). For this review, we included eligible studies located during these searches that were published between January 1 2008 and March 31 2022.

We performed an additional search on 16 April 2024 to locate STI data published between 1 April 2022 and 15 April 2024. This search covered both the peer-reviewed and grey literature, retaining the search domain pertaining to the population of interest (i.e., people who inject drugs), with an additional search domain pertaining to STI prevalence and testing coverage (see Appendix C for detail). For this search, each study was screened independently by two authors (OP and EC) at both title/abstract and full text level. Conflicts were resolved through discussion by these authors.

### 2.3. Data collection process and data items

For STI prevalence, we extracted data for the number/proportion of participants who were tested for an STI or who self-reported their STI status, number/proportion of participants with an STI diagnosis, the timeframe for the STI diagnosis (e.g., current, past year), the method for ascertaining STI status (e.g., self-report, study ascertained), and the type of diagnostic test (if performed by the study investigators). For STI testing coverage, we extracted the number/proportion of participants who reported STI testing (excluding testing undertaken by the study investigators), and the timeframe in which they were tested.

We also extracted information on publication (e.g., year of publication, literature type), study setting (e.g., study year, recruitment method, inclusion/exclusion criteria, geographic location), sociodemographic characteristics of the study population (e.g., gender [note, we have chosen to use the term gender but this term may refer to either gender or sex as this distinction was not always clear], age, sexual identity [including men who have sex with men]), sexual risk behaviours (e.g., condomless sex with a casual partner, sex work), drug use risk behaviours (e.g., reuse of needles, sharing needles), and HIV prevalence (using serological information only). As in previous reviews, we graded the methodology of studies that reported prevalence estimates. The grading considered the number of sample types, number of site types, and ascertainment method used by the study (Degenhardt et al., 2017, 2023). When studies disaggregated data by geographic location and/or year of data collection, we extracted data using the same categories. All extracted data, including grading of prevalence estimates, were double checked by a second researcher.

### 2.4. Synthesis of results

Where there were sufficient data available, regional and global estimates weighted by national population size of people who inject drugs were generated using random effects meta-analysis. Where data were insufficient, narrative synthesis was used.

Random effects meta-analysis was used to pool estimates by country first, which allowed estimation of heterogeneity across countries (rather than across studies) using the *I*^2^ statistic. We weighted country-level prevalence estimates by country-level population size of people who inject drugs (Degenhardt et al., 2023) to generate regional (countries grouped according to UNAIDS regions) and global STI prevalence estimates among people who inject drugs. Countries without prevalence data were assigned the regional estimate where ≥ 2 countries in the region had data available or the global estimate where ≤ 1 countries in the region had data available. The exception was regions with only two countries (North America and Australasia) where the regional estimate was assigned to a country missing data if the other country had available data. For meta-analysis of STI prevalence estimates, we only included estimates generated by study investigator-conducted testing (self-report results were summarised separately by narrative synthesis). For STI testing coverage, self-report estimates were retained.

For meta-analysis of syphilis prevalence, we applied a correction to point estimates based on the diagnostic test used (Tsuboi et al., 2021). This resulted in standardised estimates that approximated active syphilis, that is, a reactive result on both a treponemal and non-treponemal test. Use of only one of these tests does not allow distinction between active or past infection. Further information is provided in Appendix D.

### 2.5. Subgroup analyses

Due to gender differences in STI prevalence, we stratified meta-analyses by gender where there were sufficient data. We only included studies that reported prevalence for both men and women (i.e., we excluded studies that recruited only one gender) to ensure comparability between genders.

### 2.6. Sensitivity analyses

We conducted three sensitivity analyses. In the first, we restricted prevalence data to the most recent data available for each country (i.e., excluded data points ≥5 years older than the most recent data point for a given country). In the second, we limited analyses to estimates for people who had injected drugs during the past year. Finally, we excluded studies that recruited participants of only one gender.

### 2.7. Meta-regression

We used meta-regression to examine potential study-level and country-level sources of heterogeneity. Study-level characteristics of interest were age (mean or median), duration of injecting drug use (mean or median), and the proportion of participants who were women, men who have sex with men, reported the drug injected most frequently was a stimulant (e.g., cocaine, methamphetamine), reported lifetime incarceration, reported recent (i.e., past year) unstable housing, reported recent injecting risk behaviour (defined as receptive needle sharing), reported recent sexual risk behaviour (defined as condomless sex with a casual partner), reported recent sex work, were living with HIV (based on serological testing), and the year that data collection for the study was completed. Variables were only included in meta-regressions where ≥ 25 % of estimates had available data points.

Country-level indicators hypothesised to be associated with STI prevalence or testing coverage comprised: general population STI prevalence (specific to the STI being analysed), injecting drug use prevalence, HIV prevalence among people who inject drugs, the Human Development Index (considers life expectancy, education, and national income), the Gender Inequality Index, income inequality (measured by the Gini coefficient), World Bank country income level (low/lower middle vs. upper middle/high), needle-syringe program coverage (number of syringes distributed per person who injects drugs per year), and opioid agonist treatment coverage (proportion of people who inject drugs receiving treatment). National indicator data and their sources are provided in Appendix E.

We used generalized linear models to investigate the relationship between each factor of interest and the outcome. We logit-transformed all variables that were proportions, including the outcome. Prior to transformation, we converted zero values to half the smallest non-zero value and values of one to one minus half the smallest non-zero value. This was necessary to retain studies that reported zero prevalence of the STI and/or recruited participants of only one sex or gender. Where multiple data points were available for a country, all estimates were included and clustered by country. We investigated non-linearity by adding a quadratic term (i.e., the square of the factor being investigated). We compared the quadratic term model with the base model using the likelihood ratio test and Bayesian Information Criterion. We assumed that country-level syphilis prevalence would be associated with prevalence among people who inject drugs, so we also performed an adjusted analysis incorporating this term. The significance level was set at 0.05. Meta-analysis and meta-regression were performed in Stata (version 16; StataCorp, 2019), while data visualisation was undertaken in R (version 4.3.1; R Core Team, 2020), using the ggplot2 package (Wickham, 2016).

### 2.8. Role of the funding source

The funder of the study had no role in the study design, data collection, data analysis, data interpretation, or writing of the report.

## 3. Results

### 3.1. Study selection

**Fig. 1.**
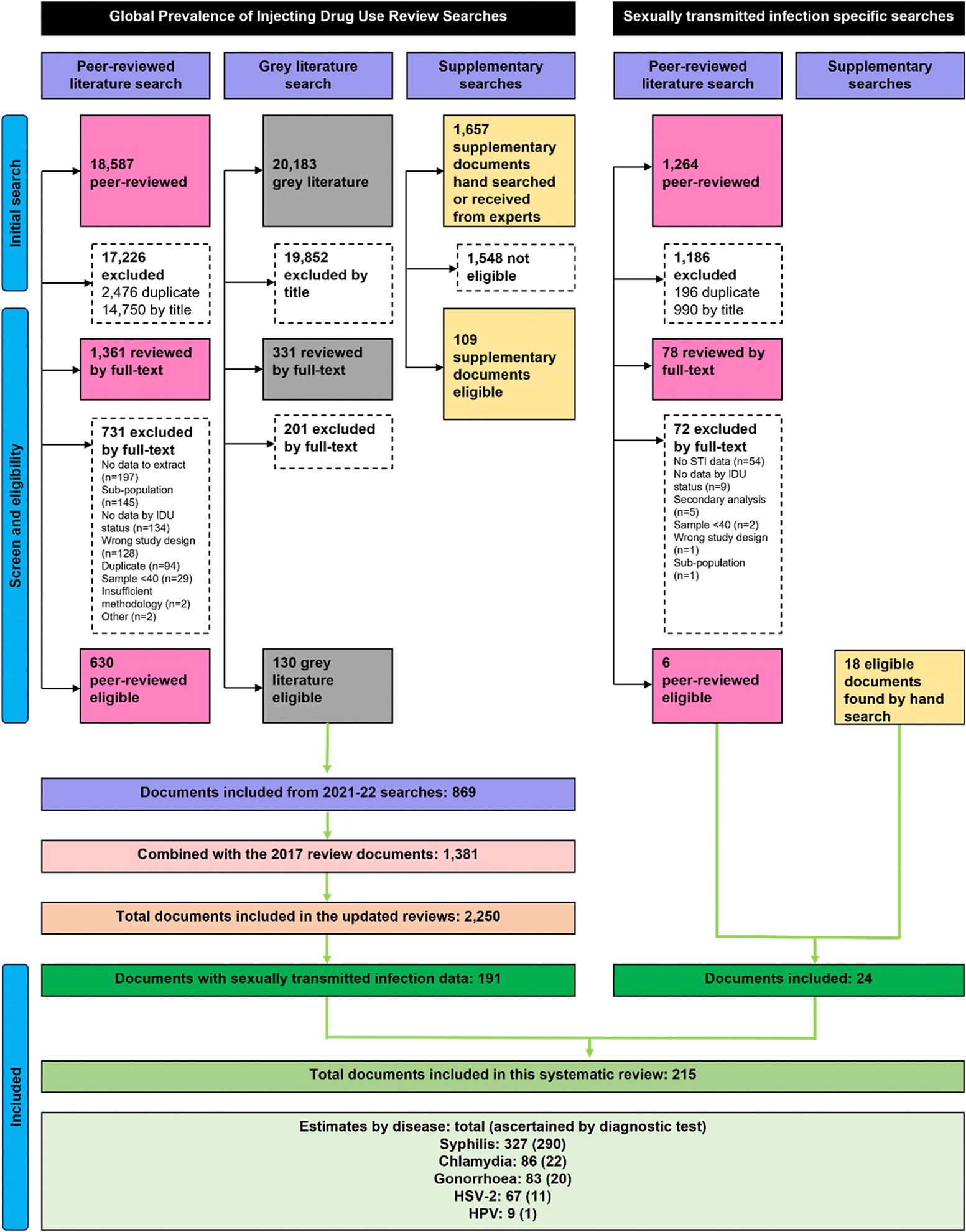
Study flowchart.

In the 2022 search for papers and reports published between January 2017 and March 2022, 40,427 documents were screened, of which 869 contained information on eligible samples of people who inject drugs and were included in the main review of bloodborne viruses and exposure to behavioural and environmental risks (Fig. 1). These documents were added to the 1381 eligible documents published since 2008 in prior searches (Degenhardt et al., 2017). Of these 2250 documents, 191 contained relevant data on STI prevalence or testing coverage.

In our search of peer-reviewed literature databases for papers containing STI data published between April 2022 and April 2024, 1264 records were identified and title/abstract screened, of which 78 were reviewed by full-text, and six deemed eligible. Through our search of relevant websites and requests to international experts, we located 18 eligible grey literature reports. In total, 215 documents were included in this review of STI prevalence and testing coverage.

### 3.2. Study characteristics

Just over half the included documents were grey literature (n = 119, 55 %). Most documents (68 %, n = 146) included samples of people who had injected drugs within the past year; 8 % (n = 18) included people with a lifetime history of injecting drugs, and the remainder did not specify the timeframe (23 %, n = 49). Approximately one fifth (23 %, n = 49) of documents were from high income countries, with 28 % (n = 61) from upper middle-income countries, 39 % (n = 83) from lower middle-income countries, and 10 % (n = 22) from low-income countries. Most studies were conducted between 2010 and 2019 (65 %, n = 140), with 28 % (n = 60) and 7 % (n = 15) conducted between 2000 and 2009 and 2020–2024, respectively.

### 3.3. Results of syntheses

Data were sparse for all outcomes of interest except syphilis prevalence (Table 1). Therefore, we performed meta-analysis for syphilis and used narrative synthesis for all remaining STIs and estimates of testing coverage.

### 3.4. Syphilis

In total, we located 327 syphilis prevalence estimates. A minority of these (n = 37, from five countries) were based on self-report and were not included in meta-analyses but are reported in Appendix K. There were two countries (Spain and Puerto Rico) that we could only locate syphilis prevalence estimates based on self-report.

The 290 serological estimates included for meta-analysis were obtained from 52 out of 190 countries with evidence of injecting drug use, which represent approximately 75 % of the global population of people who inject drugs (Appendix F). The income level of countries with data available varied: 23 % were classified as high income, 31 % were upper-middle, 35 % were lower-middle, and 12 % were low income. Most estimates were from studies that recruited participants from multiple sites (n = 270), comprising 172 that recruited a single sample type and 98 that recruited multiple sample types. A minority recruited multiple sample types from a single site (n = 15) or a single sample type from a single site (n = 20). The studies included were conducted between 2003 and 2024, inclusive, with a greater number of prevalence point estimates reported from 2003 to 2012 (n = 179) than 2013–2024 (n = 111).

Meta-analysis included 116, 720 people who inject drugs tested for syphilis (Table 2). Three regions – Eastern Europe, East and Southeast Asia, and South Asia – contributed the vast majority of data (87 % of people who inject drugs who were tested). In total, 42 % of prevalence estimates were based on both treponemal and non-treponemal positive tests, 27 % reported either treponemal-positive or non-treponemal-positive results, and 12 % used a rapid test, while 18 % did not report the diagnostic test used (Appendix F).

**Fig. 2.**
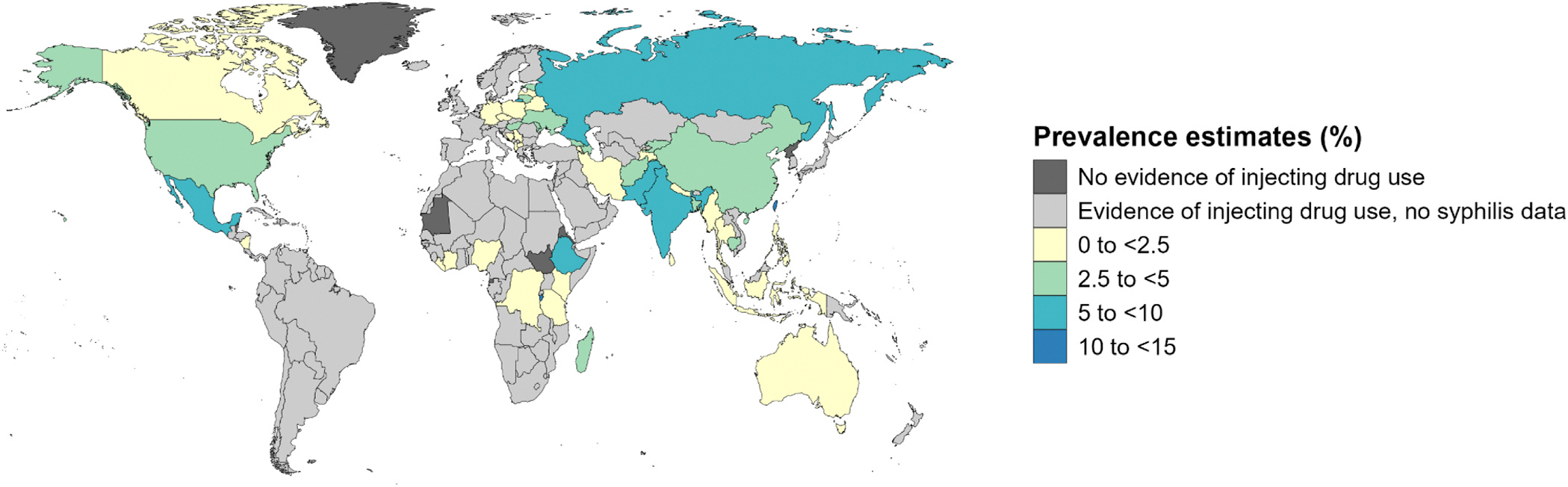
Syphilis prevalence estimates among people who inject drugs.

The global prevalence of active syphilis among people who inject drugs was estimated as 3.2 % (95 % CI: 2.3–4.6), with prevalence lowest in North America (0.1 %, 95 % CI: 0–0.3) and highest in Latin America (7.2 %, 95 % CI: 3.6–11.7; Fig. 2). There was considerable heterogeneity across countries (*I*^2^=91.8; Appendix G).

There were 12 studies from 11 countries that reported syphilis prevalence estimates for both men and women (African Development Bank Group, 2012; Azim et al., 2008; Barska and Sazonov, 2016; Global Fund, 2012; Grigoryan et al., 2013; HEALTH, 2020; Ministry of Health and Quality of Life, 2017; Phalkun Mun et al., 2018; Rahimi-Movaghar et al., 2010; Republic of Maldives, 2008; Rusch et al., 2009; Tan and Zhou, 2010), reported in Appendix H. The pooled prevalence of active syphilis for women was 8.1 % (3.6–13.7), while for men it was 2.3 % (1.4–3.5). Sensitivity analyses produced similar point prevalence estimates to the main analysis, with reduced precision (Appendix I).

### 3.5. Analysis of variables associated with syphilis prevalence

**Fig. 3.**
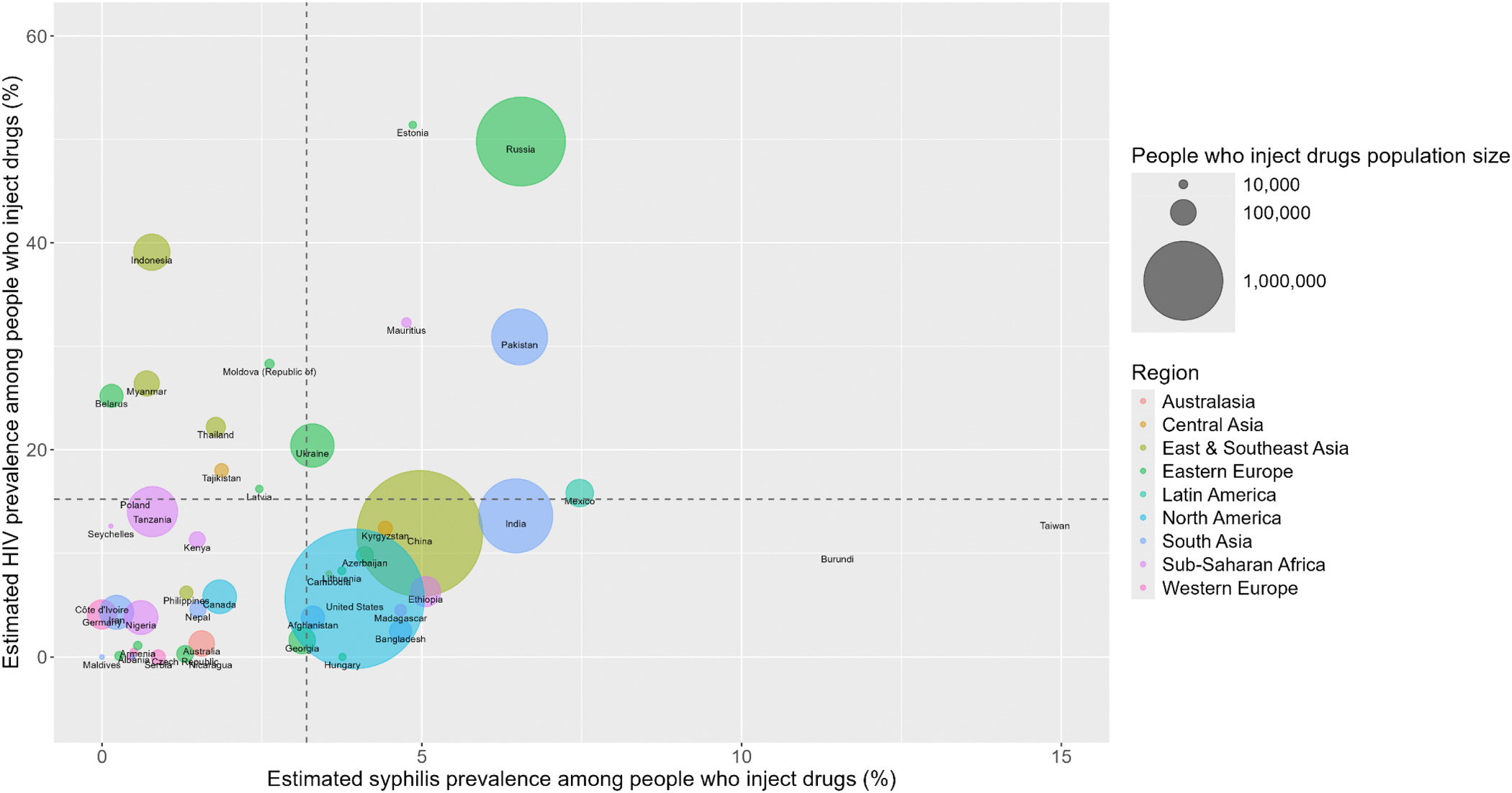
Estimated national prevalence of syphilis and HIV among people who inject drugs. Notes: The dotted lines indicate the estimated global prevalence for HIV (15.2 %) and syphilis (3.2 %) among people who inject drugs. Data for HIV prevalence and population size of people who inject drugs were obtained from a previous review (Degenhardt et al., 2023). Three countries with syphilis prevalence data (Comoros, Liberia, Republic of Congo) are not plotted as HIV prevalence data were unavailable. Five countries (Burundi, Nicaragua, Poland, Republic of Congo, Taiwan) had no population size data available; their HIV and syphilis prevalence are denoted without a corresponding bubble.

More recent studies had lower syphilis prevalence among people who inject drugs (Table 3 and Appendix J). Studies undertaken in Eastern Europe, North and Latin America or Central and South Asia were associated with higher syphilis prevalence than those conducted in Western Europe. Higher needle syringe programme coverage at the country-level was associated with lower syphilis prevalence. There was an inverted-U-shaped association between HIV and syphilis prevalence, indicated by better model fit and a significant negative quadratic term. Syphilis prevalence increased as HIV prevalence increased, but this relationship was reversed at the higher limit of HIV prevalence. This was apparent when considering both study- and country-level HIV prevalence and was also evident when estimated country-level prevalence for syphilis among people who inject drugs was plotted against HIV prevalence among people who inject drugs (Fig. 3).

Due to insufficient data points (i.e., available for *<*25 % of estimates), we could not assess the association between syphilis prevalence and the proportion of participants who identified as men who have sex with men, had even been incarcerated, were recently unstably housed, recently engaged in sex work, or predominantly injected stimulants.

### 3.6. Other STIs

Other STIs that we located data for were chlamydia, gonorrhoea, herpes simplex virus 2, human papillomavirus, and trichomoniasis (Table 1). For these STIs, estimates obtained from studies that conducted diagnostic testing are reported in Table 4 and summarised in text, while data obtained from self-report are presented in Appendix K.

We located 22 chlamydia prevalence estimates from eight countries. Estimates ranged from 0 % in Pakistan to 12.5 % in India. Most estimates were from lower middle or upper middle-income countries (n = 20, 91 %). Gonorrhoea estimates (n = 20) were obtained from the same eight countries. Prevalence ranged from 0 % in multiple countries (Hungary, India, Indonesia, Nigeria and Pakistan) to 2.0 % in the United States. There were 12 estimates from five countries of lifetime herpes simplex virus 2 infection, ranging from 3.4 % in India to 34 % in the United States. We also located one estimate from India for human papillomavirus (72.4 %).

Many studies reported composite estimates using self-report data for any STI diagnosis (116 estimates from 40 countries) or any STI symptoms (41 estimates from 32 countries; Appendix K).

### 3.7. STI testing coverage

We located 19 estimates for STI testing uptake from 15 countries (Table 5). The majority pertained to testing for any STI or did not specify which STI was tested for (n = 17); the remaining two estimates from Canada and Germany were for syphilis testing uptake. Past 12-month uptake of testing for any STI was available for eight countries and estimates ranged 8 % in Canada to 62 % in Ethiopia.

## 4. Discussion

To our knowledge, this was the first systematic review of STI prevalence and STI testing coverage among people who inject drugs. Except for syphilis, prevalence data were sparse and often reliant on self-report. STI testing coverage data were also scarce. For syphilis, we found evidence from 52 of 190 countries with evidence of injecting drug use (representing approximately 75 % of the global population of people who inject drugs; Degenhardt et al., 2023) and estimated the global prevalence as 3.2 %, six times higher than general population estimate (0.5 %; Rowley et al., 2019), but lower than men who have sex with men (7.5 %; Tsuboi et al., 2021). Syphilis prevalence varied by region and gender and was associated with HIV prevalence.

Our findings point to an urgent need for more consistent and rigorous data collection to better understand the epidemiologyof STIs among people who inject drugs. Many studies relied on self-report to ascertain previous STI infections, which is dependent on the participant seeking testing and subject to recall and social desirability biases. Moreover, many reported aggregate outcomes, i.e., ‘any STI’, or lifetime estimates which do not provide sufficient information to investigate the epidemiology of STIs. Estimates for ‘any STI symptoms’ were also common and given many STIs are asymptomatic, are likely to be underestimates. STI testing could be integrated into existing biobehavioural surveys that test for bloodborne viruses, although for some STIs (e.g., chlamydia), this would require additional resources to collect a non-blood sample. Although there were sufficient data to estimate global syphilis prevalence, approximately half the available data points were more than a decade old and were particularly sparse or absent for Australasia, Central Asia, Latin America, North America, Sub-Saharan Africa, and Western Europe. The accuracy of syphilis prevalence estimates would also be improved by confirmatory testing for active infection (i.e., use of both a non-treponemal and treponemal test), although this is more resource intensive, cannot be done at point-of-care, and interpretation of test results can be complex (Pham et al., 2022). Another limitation of the available data was a lack of subgroup analyses, which limited our ability to examine intersectional risk factors that increase the risk of STIs (e.g., sexuality, type of drugs used, engagement in transactional sex). Understanding whether prevalence varies among subpopulation of people who inject drugs would enable tailoring of control strategies to reach people most at risk and more efficiently reduce disease incidence among this population.

Higher than general population syphilis prevalence confirms people who inject drugs are a key population for the scale up of STI control measures. Control of STIs requires widespread, stigma-free access to prevention measures (e.g., condoms) and an understanding of who and how often to screen, which enables timely diagnosis and linkage to treatment and prevention of onward transmission. The WHO and European Union Drugs Agency both recommend screening people who inject drugs for STIs, although neither advise how often this should occur (European Centre for Disease Prevention and Control (ECDC) and European Monitoring Centre for Drugs and Drug Addiction (EMCDDA), 2023; World Health Organization, 2022). Indeed, there is little consensus on the optimal screening interval for STIs among the general population (Medland and Guy, 2024) but it likely varies based on individual-level factors, including gender, sexuality, type of substance used, and engagement in transactional sex or sex work, and population-level factors, including local prevalence.

Accessible healthcare is a powerful determinant of STI prevalence; previous ecological and epidemiological studies have demonstrated that high prevalence in some populations is a consequence of reduced access to testing, rather than increased sexual risk behaviours (Fairley et al., 2022). Although we found insufficient data to describe access to STI testing among people who inject drugs, this population experience barriers to primary healthcare (Motavalli et al., 2021). Therefore, reducing structural barriers to STI testing and treatment is essential. The WHO recommends integrating STI testing in existing bloodborne virus prevention programs, ideally in a community-led setting trusted by people who inject drugs (World Health Organization, 2022). Qualitative evidence suggests this is acceptable to the population (Hinkes et al., 2024; Owens et al., 2020). Self-sampling may also increase participation and is recommended by the WHO (World Health Organization, 2020), although research specific to people who inject drugs is needed.

We found that syphilis prevalence increased with HIV prevalence, although this trend reversed at the upper end of HIV prevalence. We expected the concomitant increase given syphilis increases HIV transmission (Roberts and Klausner, 2016) and this association has previously been demonstrated at the individual level among a prospective cohort of people who inject drugs (Pines et al., 2015). The finding that this association reversed was not expected. It might be an artefact of the countries with available data or that syphilis control is a higher priority in countries with HIV prevalence. Nevertheless, our findings suggest that scale-up of syphilis testing is particularly important in areas with medium-high HIV prevalence. Syphilis can be tested concurrently with HIV in a point-of-care test, which may be a cost- and time-effective method in countries where HIV testing is already common, but confirmatory testing after a positive point-of-care test is required.

There are also emerging STI prevention measures that might be considered for people who inject drugs. Doxycycline as post-exposure prophylaxis (‘doxyPEP’) is highly effective at preventing syphilis and chlamydia and moderately effective for gonorrhoea, but potential benefits must be balanced with the increased risk of antimicrobial resistance (Cornelisse et al., 2024). Currently, doxyPEP is recommended for syphilis prevention among gay, bisexual and other men who have sex with men, the only population where it has been demonstrated to be effective (Cornelisse et al., 2024). Importantly, doxyPEP is useful to prevent incident infection, but we cannot discern whether the high syphilis prevalence we observed among people who inject drugs is due to high incidence or long-term infections that had not been detected and treated. More research investigating incidence among this population is needed to make this distinction and inform STI control strategies (including the use of doxyPEP) that consider local epidemiological context. It is also possible that vaccination for chlamydia and gonorrhoea will become available in the future (de la Maza et al., 2021; Vincent and Jerse, 2019), and people who inject drugs should be considered a priority population during rollout.

While the aforementioned strategies may directly reduce STI incidence among people who inject drugs, consideration of root causes is also important. It is hypothesised that drug dependence increases the reliance on transactional sex and/or sex work, which in turn increases the risk of STI transmission (Strathdee et al., 2021). For people with opioid dependence, OAT may reduce engagement in these behaviours and serve as an opportunity to link people to STI testing and treatment; the latter association has previously been observed for hepatitis C (Grebely et al., 2021). Ministries of Health should consider recommending STI testing for OAT clients in prescriber guidelines. The increased STI risk associated with use of methamphetamine and other stimulants (Strathdee et al., 2021), whether deliberately to enhance sex or not, must also be considered. Tailoring health promotion messaging or STI testing to people who use or inject these substances might be beneficial.

### 4.1. Limitations

We have identified and highlighted important gaps in the existing evidence that limited our ability to synthesise STI prevalence and testing coverage data. Our meta-analysis of syphilis prevalence was subject to these data limitations. Many syphilis estimates were over a decade old and as syphilis incidence has increased over the past decade, we may have underestimated current prevalence There is likely to be variation in prevalence within a country and multiple countries had only one point estimate that was drawn from a single city or region. We also relied on correction for different syphilis diagnostic methods used, which is a somewhat crude estimation and might be affected by population prevalence. However, this is an inherent limitation of the complicated nature of syphilis testing, and the prevalence estimates we compared our findings to used similar methods (Rowley et al., 2019; Tsuboi et al., 2021). We excluded studies that restricted the sample to a subpopulation, including people living with HIV and people who are incarcerated, who may have higher syphilis prevalence than the broader population of people who inject drugs. Finally, meta-regression does not assess causation, so care should be taken interpreting the results of those analyses; significant associations we observed between country-level indicators and prevalence may be an artefact of the countries with available data.

## 5. Conclusion

Data on STI prevalence and testing coverage among people who inject drugs are limited, with data particularly sparse for STIs other than syphilis. We estimate that global syphilis prevalence among people who inject drugs is six times higher than the general population, indicating they are a priority population for prevention and control measures. Integrating STI testing into existing bloodborne virus programs for people who inject drugs may be an efficient way to achieve this. Our understanding of the epidemiology of STIs among this population would be improved by more data of better quality and subgroup analyses that examine the role of intersectional risk factors.

## Supplementary Material

1

## Figures and Tables

**Table 1:** Availability of STI prevalence estimates, disaggregated by UNAIDS region.

	Syphilis		Chlamydia		Gonorrhoea		Herpes simplex virus 2		Human papillomavirus		Trichomoniasis	
	Diagnostic testing N countries (n estimates)	Self-report N countries (n estimates)	Diagnostic testing N countries (n estimates)	Self-report N countries (n estimates)	Diagnostic testing N countries (n estimates)	Self-report N countries (n estimates)	Diagnostic testing N countries (n estimates)	Self-report N countries (n estimates)	Diagnostic testing N countries (n estimates)	Self-report N countries (n estimates)	Diagnostic testing N countries (n estimates)	Self-report N countries (n estimates)

**Australasia**	1 (1)	-	-	-	-	-	-	-	-	-	-	-
**Caribbean**		1 (2)	-	1 (1)	-	1 (2)	-	1 (1)	-	-	-	-
**Central Asia**	2 (3)	-	-	-	-	-	-	-	-	-	-	-
**East and Southeast Asia**	7 (78)	-	2 (4)	-	2 (4)	3 (19)	-	-	-	-	-	-
**Eastern Europe**	14 (75)	1 (1)	1 (1)	3 (19)	1 (1)	2 (41)	1 (1)	2 (18)	-	-	-	2 (2)
**Latin America**	2 (5)	-	-	-	-	-	-	-	-	-	-	-
**Middle East & North Africa**	-	-	-	-	-	-	-	-	-	-	-	-
**North America**	2 (4)	2 (33)	1 (1)	2 (43)	1 (1)	-	1 (2)	2 (35)	-	2 (7)	-	-
**Pacific Island states & terr.**	-	-	-	-	-	-	-	-	-	-	-	-
**South Asia**	8 (93)	-	2 (14)	-	2 (12)	-	3 (17)	-	1 (1)	-	-	-
**Sub-Saharan Africa**	12 (24)	-	2 (2)	-	2 (2)	-	-	-	-	-	-	-
**Western Europe**	4 (7)	1 (1)	-	1 (1)	-	1 (1)	-	1 (1)	-	1 (1)	-	-
**Global**	**52 (290)**	**5 (37)**	**8 (22)**	**7 (64)**	**8 (20)**	**7 (63)**	**5 (12)**	**6 (55)**	**1 (1)**	**1 (8)**	**0 (0)**	**2 (2)**

**Table 2:** Prevalence of syphilis among people who inject drugs, disaggregated by UNAIDS region.

	Number of countries with evidence of injecting drug use	Number of countries with syphilis prevalence data	Number of point prevalence data	Study sample size (range)	Number of people who inject drugs tested (column %)	Number of people who inject drugs with positive syphilis test	Uncorrected syphilis prevalence (95 % CI)	Corrected syphilis prevalence (95 % CI)

**Australasia**	2	1	1	128	128 (0.1)	3	2.7 (0.5–6.5)	1.9 (5.5)
**Caribbean**	8	-	-	-	-	-	-	-
**Central Asia**	5	2	3	488–1916	3308 (2.8)	166	12.0 (9.6–14.7)	3.2 (2.3–4.3)
**East and Southeast Asia**	17	7	78	55–2530	33068 (28.3)	1255	6.3 (5.0–7.9)	4.6 (3.7–5.7)
**Eastern Europe**	17	14	75	50–9407	33159 (28.4)	1406	8.1 (6.8–9.8)	4.7 (3.7–6)
**Latin America**	19	2	5	41–1056	1609 (1.4)	114	7.4 (3.6–12)	7.2 (3.6–11.7)
**Middle East & North Africa**	21	-	-	-	-	-	-	-
**North America**	2	2	4	71–150	430 (0.4)	16	0.1 (0–0.3)	0.1 (0–0.3)
**Pacific Island states & terr.**	15	-	-	-	-	-	-	-
**South Asia**	9	8	93	57–4216	35097 (30.1)	1313	5.5 (3.4–7.9)	5.5 (3.5–8.0)
**Sub-Saharan Africa**	44	12	24	30–1701	7802 (6.7)	231	1.8 (0.7–4.4)	1.8 (0.7–4.4)
**Western Europe**	31	4	7	99–584	2119 (1.8)	19	0.3 (0.1–1.2)	0.2 (0.1–0.9)
**Global**	**190**	**52**	**290**	**30–9407**	**116720 (100)**	**4523**	**4.4 (3.2–6.0)**	**3.2 (2.3–4.6)**

**Notes.** CI = confidence interval. See Appendix D for information of syphilis prevalence correction, Appendix E for sources of these estimates, and Appendix G for country-level estimates and heterogeneity.

**Table 3:** The association between study-level and country-level characteristics with syphilis prevalence.

	Unadjusted analysis				Adjusted analysis			
	N*	β	95 % CI	*p*	N*	β	95 % CI	*p*

***Study-level characteristics***								
% female/woman	230	0.08	−0.07, 0.24	0.292	230	0.10	−0.03, 0.23	0.125
Mean/median age	209	−0.01	−0.07, 0.05	0.804	209	−0.00	−0.07, 0.06	0.888
Mean/median duration injecting drug use	99	0.00	−0.05, 0.06	0.918	99	0.00	−0.03, 0.05	0.511
Recent injecting risk behaviour^a^	155	0.13	−0.04, 0.30	0.124	155	0.13	−0.03, 0.30	0.118
Recent sexual risk behaviour^b^	107	0.02	−0.18, 0.23	0.825	107	0.03	−0.15, 0.22	0.733
HIV prevalence	238	−0.03	−0.36, 0.29	0.836	238	−0.27	−0.32, 0.27	0.858
HIV prevalence with quadratic term	238	−0.89	−1.43, −0.35	**0.001**	238	−0.88	−1.38, −0.38	**0.001**
Quadratic term	-	−0.17	−0.26, −0.82	**< 0.001**	-	−0.17	−0.26, −0.08	**< 0.001**
Year study completed	290	−0.09	−0.14, −0.03	**0.002**	290	−0.09	−0.15, −0.03	**0.006**
***Country-level characteristics***								
UNAIDS region								
Western Europe (reference)	290	-	-	-	289	-	-	-
Australasia	290	1.11	0.35, 1.87	**0.004**	290	1.54	0.48, 1.83	**0.001**
Eastern Europe	290	1.11	0.05, 2.17	**0.040**	289	1.09	0.08, 2.10	**0.034**
East & Southeast Asia	290	0.76	−0.53, 2.06	0.248	289	1.10	−0.29, 2.49	0.121
North & Latin America	290	1.98	1.07, 2.89	**< 0.001**	289	2.08	1.22, 2.94	**< 0.001**
Central & South Asia	290	1.39	0.43, 2.36	**0.005**	289	1.73	0.62, 2.85	**0.002**
Sub-Saharan Africa	290	1.06	−0.23, 2.35	0.107	289	1.58	−0.20, 3.36	0.081
Population syphilis prevalence	290	−0.10	−0.64, 0.44	0.710	-	-	-	-
Population syphilis prevalence with quadratic term	290	−5.86	−11.24, −0.47	**0.033**	-	-	-	-
Quadratic term	-	−0.54	−1.03, −0.04	**0.034**	-	-	-	-
Injecting drug use prevalence	237	−0.13	−0.51, 0.25	0.494	237	−0.20	−0.62, 0.23	0.361
HIV prevalence among people who inject drugs	274	0.03	−0.35, 0.40	0.883	274	0.04	−0.33, 0.40	0.849
HIV prevalence among people who inject drugs with quadratic term	274	−0.81	−1.63, 0.00	0.050	274	−0.91	−1.70, −0.13	**0.023**
Quadratic term	-	−0.15	−0.25, −0.04	**0.007**	-	−0.16	−0.27, −0.06	**0.002**
Opioid agonist treatment coverage^c^	258	−0.22	−0.49, 0.06	0.127	258	−0.21	−0.47, 0.04	0.106
Needle syringe program coverage^d^	251	−0.004	−0.006, −0.002	**< 0.001**	251	−0.004	−0.006, −0.002	**< 0.001**
Human Development Index	289	0.15	−3.22, 3.52	0.930	289	−0.64	−4.04, 2.86	0.711
Gender Inequality Index	286	−0.05	−2.52, 2.43	0.970	286	0.60	−2.27, 3.46	0.683
Income inequality (Gini coefficient)	273	0.02	−0.06, 0.09	0.700	272	0.02	−0.06, 0.10	0.625
World Bank income level								
Low/lower middle (reference)	290	-	-	-	290	-	-	-
Upper middle/high	290	−0.03	−0.91, −.85	0.955	290	−0.20	−1.06, 0.66	0.651

**Notes.** CI = confidence interval. Bolded values denote statistical significance, i.e., *p* < 0.05.

^ Adjusted models are independent of each other and adjusted for general population syphilis prevalence only.

* The maximum number of data points for each model is 290 (the number of syphilis prevalence estimates among people who inject drugs).

a Defined as any of receptive or distributive needle sharing or reuse of needles during the past 12 months.

b Defined as unprotected sex with a non-regular partner during the past 12 months.

c Number of people in opioid agonist treatment per 100 people who inject drugs.

d Number of needles/syringes distributed per person who injects drugs per year.

**Table 4:** Studies reporting prevalence of sexually transmitted infections other than syphilis among people who inject drugs.

Country and reference	Study year	Income level	Geographic coverage	Location (if not national)	Literature grade	Method grade	Recruitment method	Recency of injecting drug use	No. tested for STI (N)	Prevalence of STI (%)	No. tested by gender (N)	Prevalence by gender (%)

***Chlamydia***												
Hungary (Gyarmathy et al., 2009)	2006	High	City	Budapest	A1	A	Snowballing	1 month	186	12.0	-	-
India (FHI 360, 2011)	2010	Lower middle	City	Bishnupur	B2	B1	RDS	6 months	410	1.0	M: 410	M: 1.0
India (FHI 360, 2011)	2010	Lower middle	City	Churachandpur	B2	B1	RDS	6 months	411	1.9	M: 411	M: 1.9
India (FHI 360, 2011)	2010	Lower middle	City	Mumbai-Thane	B2	B1	RDS	6 months	327	0.8	M: 327	M: 0.8
India (FHI 360, 2011)	2010	Lower middle	City	Phek	B2	B1	RDS	6 months	418	12.5	M: 418	M: 12.5
India (FHI 360, 2011)	2010	Lower middle	City	Wokha	B2	B1	RDS	6 months	411	5.6	M: 411	M: 5.6
India (Mahanta et al. 2008)	2008	Lower middle	City	Bishnupur	A1	B1	RDS	6 months	420	1.7	M: 420	M: 1.7
India (Mahanta et al. 2008)	2008	Lower middle	City	Churachandpur	A1	B1	RDS	6 months	419	2.1	M: 419	M: 2.1
India (Mahanta et al. 2008)	2008	Lower middle	City	Mumbai-Thane	A1	B1	RDS	6 months	355	0.7	M: 355	M: 0.7
India (Mahanta et al. 2008)	2008	Lower middle	City	Phek	A1	B1	RDS	6 months	440	11.4	M: 440	M: 11.4
India (Mahanta et al. 2008)	2008	Lower middle	City	Wokha	A1	B1	RDS	6 months	420	11.0	M: 420	M: 11.0
India (Goswami et al., 2014)	2006	Lower middle	Sub-national	Manipur	A1	B1	RDS	6 months	839	1.9	M: 839	M: 1.9
India (Goswami et al., 2014)	2006	Lower middle	Sub-national	Nagaland	A1	B1	RDS	6 months	821	10.9	M: 821	M: 10.9
Indonesia (Morineau et al., 2012)	2007	Upper middle	City	Medan	A1	B1	RDS	Unspecified	234	6.0	-	-
Indonesia (Morineau et al., 2012)	2007	Upper middle	City	Jakarta	A1	B1	RDS	Unspecified	246	5.3	-	-
Indonesia (Morineau et al., 2012)	2007	Upper middle	City	Surabaya	A1	B1	RDS	Unspecified	249	6.8	-	-
Kenya (Tun et al., 2015)	2011	Lower middle	City	Nairobi	A1	B1	RDS	3 months	269	3.4	-	-
Nigeria (Tun et al., 2013)	2010	Lower middle	City	Lagos	A1	C	RDS	12 months	328	0.9	M: 328	M: 0.9
Pakistan (Platt et al., 2009)	2007	Lower middle	City	Abbottabad	A1	B1	RDS	1 month	100	0	-	-
Pakistan (Platt et al., 2009)	2007	Lower middle	City	Rawalpindi	A1	B1	RDS	1 month	301	1.0	-	-
Thailand (Visavakum et al., 2016)	2010	Upper middle	Sub-national	Songkhla	A1	B2	RDS	6 months	199	1.5	-	-
United States (Cari et al., 2020)	2018	High	City	Kentucky	A2	C	Convenience	Unspecified	47	6.4	-	-
***Gonorrhoea***												
Hungary (Gyarmathy et al., 2009)	2006	High	City	Budapest	A1	A	Snowballing	1 month	186	0	-	-
India (FHI 360, 2011)	2010	Lower middle	Sub-national	Churachandpur	B2	B1	RDS	6 months	411	0.7	M: 411	M: 0.7
India (FHI 360, 2011)	2010	Lower middle	Sub-national	Mumbai-Thane	B2	B1	RDS	6 months	327	0	M: 327	M: 0
India (FHI 360, 2011)	2010	Lower middle	Sub-national	Phek	B2	B1	RDS	6 months	418	2.0	M: 418	M: 2.0
India (FHI 360, 2011)	2010	Lower middle	Sub-national	Wokha	B2	B1	RDS	6 months	411	1.3	M: 411	M: 1.3
India (Mahanta et al. 2008)	2008	Lower middle	Sub-national	Bishnupur	A1	B1	RDS	6 months	420	0.3	M: 420	M: 0.3
India (Mahanta et al. 2008)	2008	Lower middle	Sub-national	Churachandpur	A1	B1	RDS	6 months	419	0	M: 419	M: 0
India (Mahanta et al. 2008)	2008	Lower middle	Sub-national	Phek	A1	B1	RDS	6 months	440	0.6	M: 440	M: 0.6
India (Mahanta et al. 2008)	2008	Lower middle	Sub-national	Wokha	A1	B1	RDS	6 months	420	1.6	M: 420	M: 1.6
India (Goswami et al., 2014)	2006	Lower middle	City	Manipur	A1	B1	RDS	6 months	839	0.2	M: 839	M: 0.2
India (Goswami et al., 2014)	2006	Lower middle	City	Nagaland	A1	B1	RDS	6 months	821	1.9	M: 821	M: 1.9
Indonesia (Morineau et al., 2012)	2007	Upper middle	Sub-national	Jakarta	A1	B1	RDS	Unspecified	234	1.3	-	-
Indonesia (Morineau et al., 2012)	2007	Upper middle	Sub-national	Medan	A1	B1	RDS	Unspecified	249	0	-	-
Indonesia (Morineau et al., 2012)	2007	Upper middle	Sub-national	Surabaya	A1	B1	RDS	Unspecified	249	1.2	-	-
Kenya (Tun et al., 2015)	2011	Lower middle	City	Nairobi	A1	B1	RDS	3 months	269	1	-	-
Nigeria (Tun et al., 2013)	2010	Lower middle	City	Lagos	A1	C	RDS	12 months	328	0	M: 328	M: 0
Pakistan (Platt et al., 2009)	2007	Lower middle	City	Abbottabad	A1	B1	RDS	1 month	100	0	-	-
Pakistan (Platt et al., 2009)	2007	Lower middle	City	Rawalpindi	A1	B1	RDS	1 month	301	1.0	-	-
Thailand (Visavakum et al., 2016)	2010	Upper middle	Sub-National	Songkhla	A1	B2	RDS	6 months	199	0.5	-	-
United States (Cari et al., 2020)	2018	High	City	Kentucky	A2	C	Convenience	Unspecified	49	2.0	-	-
***Herpes simplex virus***												
Afghanistan (Ruisenor-Escudero, 2014)	2009	Low	City	Herat	A1	B1	RDS	1 month	109	5.5	M: 109	M: 5.5
Afghanistan (Ruisenor-Escudero, 2014)	2009	Low	City	Kabul	A1	B1	RDS	1 month	286	8.4	M: 286	M: 8.4
Afghanistan (Ruisenor-Escudero, 2014)	2009	Low	City	Mazar-i-Sharif	A1	B1	RDS	1 month	102	20.6	M: 102	M: 20.6
Estonia (Uuskula et al., 2013)	2012	High income	City	Kohtla-Jarve	A2	C	Snowballing	1 month	600	32	-	-
India (Ghosh et al., 2012)	2012	Lower middle	Sub-National	West Bengal	A1	C	Convenience	Unspecified	58	3.4	M: 58	M: 3.4
India (Panda et al., 2014)	2010	Lower middle	Sub-National	Punjab	A1	B1	Probability	3 months	1165	10	M: 1165	M: 10
India (FHI 360, 2011)	2010	Lower middle	Sub-National	Mumbai-Thane, Bishnupur, Churachandpur, Phek, Wokha	B2	B1	RDS	6 months	198	17.2	M: 198	M: 17.2
India (Mahanta et al. 2008)	2008	Lower middle	City	Bishnupur, Churachandpur, Phek, Wokha, Mumbai/Thane	Al	B1	RDS	6 months	227	15.9	M: 227	M: 15.9
Pakistan (Platt et al., 2009)	2007	Lower middle	City	Abbottabad	Al	B1	RDS	1 month	100	6.0	-	-
Pakistan (Platt et al., 2009)	2007	Lower middle	City	Rawalpindi	Al	B1	RDS	1 month	301	11.0	-	-
United States (Havens et al., 2013)	2010	High income	Sub-National	Appalachian Kentucky	Al	B1	RDS	Lifetime	392	12.5	-	-
United States (Oster et al., 2014)	2010	High income	National		Al	A	Probability	Lifetime	416	34.0	-	-
***Human papillomavirus***												
India (Ghosh et al., 2012)	2012	Lower middle	Sub-national	West Bengal	Al	C	Convenience	Unspecified	58	72.4	F: 58	F: 72.4

**Notes.** – data not disaggregated by sex/gender. STI = sexually transmitted infection; RDS = respondent-driven sampling. For gender-specific estimates, M=man/male, F=woman/female. Where studies aggregated data over multiple years, the most recent year is reported as the study year; where studies reported disaggregated data over multiple years, prevalence for each year was reported separately. Literature grade key: A1 = peer- reviewed journal article; A2 = abstract of published article only; B1 = published book/report/monograph from scholarly or commercial publisher; B2 = published book/report/monograph from international governmental or monitoring organisation; B3 = published book/report/monograph from other source; C = conference abstract; D = other unpublished report. Method grade key: A = multisite study with > 1 sample types (e.g. needle-syringe programmes, drug treatment centres), B1 = single sample type and multiple sites; B2 = multiple sample types and a single site; C = single sample type and single site. Prevalence studies that used self-report data are not included here, for these data, see Appendix K.

**Table 5:** Studies reporting coverage of sexually transmitted infection testing among people who inject drugs.

Country and reference	Study year	Income level	Geographic coverage	Location (if not national)	Literature grade	Method grade	Recruitment method	Recency of injecting	Timeframe of STI test	STI tested for	Sample size (N)	STI testing coverage estimate (%)	Gender-specific sample size (N)	Gender-specific STI testing coverage estimate (%)

Australia (Agramunt et al., 2024)	2023	High	National		B3	A	Convenience	6 months	6 months	Any/Unspecified	802	24		
Australia (Peacock, 2019)	2019	High	National		B3	A	Convenience	6 months	12 months	Any/Unspecified	855	46		
Australia (Stafford and Burns, 2011)	2010	High	National		B3	A	Convenience	6 months	12 months	Any/Unspecified	902	58		
Azerbaijan (Ministry of Health the Republic of Azerbaijan, 2008)	2008	Upper middle	City	Sumgait	B3	B1	Convenience	1 month	12 months	Any/Unspecified	150	17		
Bangladesh (The Global Fund, 2021)	2020	Lower middle	Sub-National	Narayanganj, Cumilla, Gazipur, Dhaka, Rajshahi, Chapainawabgani, Barishal, Mymensingh	B2	A	RDS	1 month	12 months	Any/Unspecified	3033	23		
Benin (Plan Benin, 2015)	2015	Lower middle	National		B2	B1	Convenience	12 months	12 months	Any/Unspecified	383	52		
Burundi (Ministere de la, 2022)	2021	Low	National		B2	A	RDS	6 months	3 months	Any/Unspecified	586	23		
Canada (Pant Pai et al., 2013)	2012	High	City	Montreal	C	B1	Convenience	1 month	Lifetime/Unspecified	Syphilis	109	59		
Canada (Caldarelli et al., 2013)	2012	High	City	Middlesex-London	B3	B1	Convenience	6 months	12 months	Any/Unspecified	204	8	M: 150 F: 54	M: 4 F: 19
China (Zhang et al., 2010)	2006	Upper middle	Sub-National	Sichuan	A1	A	Snowballing	3 months	12 months	Any/Unspecified	4308	18		
China (Lau et al., 2008)	2005	Upper middle	Sub-National	Sichuan	A1	B1	Snowballing	6 months	12 months	Any/Unspecified	1923	28	F: 1923	F: 28
Ethiopia (Deyessa et al., 2020)	2019	Low	City	Addis Ababa	A1	B1	RDS	3 months	12 months	Any/Unspecified	276	62		
Germany (Zimmermann et al., 2023)	2022	High	National		B2	A	Convenience	12 months	12 months	Syphilis	563	16		
Indonesia (Persaudaraan Korban Napza Indonesia (PKNI), 2015)	2015	Upper middle	City	Jakarta	B3	C	RDS	12 months	12 months	Any/Unspecified	326	10		
Kazakhstan (Rosenkranz et al., 2016)	2013	Upper middle	Sub-National	East Kazakhstan oblast, Karaganda oblast	A1	A	RDS	1 month	Lifetime/Unspecified	Any/Unspecified	600	53		
Kenya (Syvertsen et al., 2015)	2014	Lower middle	City	Kisumu	A1	B1	Not specified	1 month	Lifetime/Unspecified	Any/Unspecified	151	34	M: 127 F: 24	M: 32 F: 50
Kyrgyzstan (Rosenkranz et al., 2016)	2013	Lower middle	Sub-National	Bishkek, Osh oblast, Chuy oblast	A1	A	RDS	1 month	Lifetime/Unspecified	Any/Unspecified	900	69		
Malaysia (Disease Control Division Ministry of Health Malaysia, 2019)	2017	Upper middle	National		B3	A	RDS	1 month	3 months	Any/Unspecified	1413	2	M: 1413	M: 2
Morocco (Johnston, 2017)	2017	Lower middle	Regional	Northern Moroccol (Tangier, Tetouan, Nador)	B3	A	RDS	6 months	3 months	Any/Unspecified	451	24		

**Notes.** STI = sexually transmitted infection; RDS = respondent-driven sampling. For gender-specific estimates, M=man/male, F=woman/female. Where studies aggregated data over multiple years, the most recent year is reported as the study year; where studies reported disaggregated data over multiple years, prevalence for each year is reported separately. Literature grade key: A1 = peer-reviewed journal article; A2 = abstract of published article only; B1 = published book/report/monograph from scholarly or commercial publisher; B2 = published book/report/monograph from international governmental or monitoring organisation; B3 = published book/report/monograph from other source; C = conference abstract; D = other unpublished report. Method grade key: A = participants recruited from multiple settings using multiple geographical locations; B1 = participants recruited from multiple settings in a single geographical location; B2 = participants recruited from single setting in multiple geographical locations; C = participants recruited from a single setting within a single geographical location.

## Appendix A. Supporting information

Supplementary data associated with this article can be found in the online version at doi:10.1016/j.drugalcdep.2025.112732.
