# Supplementary material for: Sexually transmitted infection prevalence and testing coverage among people who inject drugs: A systematic matic review
Source: Drug Alcohol Depend. Author manuscript; Available in PMC 2026 Jun 16. (PMC13270363; doi:10.1016/j.drugalcdep.2025.112732)
Supplement: 1 [file NIHMS2176754-supplement-1.pdf]

Sexually transmitted infection prevalence and testing coverage among people who inject drugs: a systematic review

Supplementary appendix

Olivia Price[1], Paige Webb[1], Jason Grebely[2], Amy Peacock[1,3], Nicholas Medland[2], Phillip Read[2,3], Emily Cooke[1], Matt Hickman[5], Peter Vickerman[5], Louisa Degenhardt[1]

1. National Drug and Alcohol Research Centre, UNSW, Sydney, Australia
2. School of Psychological Sciences, University of Tasmania, Hobart, Australia
3. Kirby Institute, UNSW, Sydney, Australia
4. Kirketon Road Centre, South Eastern Sydney Local Health District, Sydney, NSW, Australia
5. Population Health Science, Bristol Medical School, University of Bristol, Bristol, UK

Appendix A. PRISMA checklist. 3

Appendix B. GATHER checklist. 5

Appendix C. Additional literature search. 6

Appendix D. Syphilis diagnostic test correction factors used to standardise syphilis prevalence estimates. 8

Appendix E. Country-level socioeconomic, development and health characteristic values (grouped by UNAIDS region) 9

Appendix F. Study-level information for syphilis prevalence estimates among people who inject drugs. 13

Appendix G. Country-level syphilis prevalence estimates, grouped by UNAIDS region. 30

Appendix H. Syphilis prevalence estimates stratified by sex/gender. 35

Table H.1. Syphilis prevalence estimates located among people who inject drugs and identify as man/male. 35

Table H.2. Syphilis prevalence estimates located among people who inject drugs and identify as woman/female. 42

Appendix I. Sensitivity analyses of syphilis prevalence estimates. 44

Table I.1. Prevalence of syphilis among people who inject drugs, restricted to the most recent data for each country (i.e., data points within five years of the most recent data point). 44

Table I.2. Prevalence of syphilis among people who inject drugs, restricted to people who had recently injected drugs (i.e., within the past 12 months). 45

Table I.3. Prevalence of syphilis among people who inject drugs, excluding samples that only reported syphilis prevalence for one gender/sex. 46

Appendix J. Association between study-level and country-level characteristics and syphilis prevalence. 47

Figure J.1. Association between study-level demographic and behavioural profiles of people who inject drugs with syphilis prevalence. 47

Figure J.2. Association between country-level demographic and behavioural profiles of people who inject drugs with syphilis prevalence. 48

Appendix K. Studies reporting prevalence of sexually transmitted infections using participant self-report data. 49

Appendix A. PRISMA checklist.

Section/topic	#	Checklist item	Reported on page #
**TITLE**			
Title	1	Identify the report as a systematic review, meta-analysis, or both.	1
**ABSTRACT**			
Structured summary	2	Provide a structured summary including, as applicable: background; objectives; data sources; study eligibility criteria, participants, and interventions; study appraisal and synthesis methods; results; limitations; conclusions and implications of key findings; systematic review registration number.	3
**INTRODUCTION**			
Rationale	3	Describe the rationale for the review in the context of what is already known.	7
Objectives	4	Provide an explicit statement of questions being addressed with reference to participants, interventions, comparisons, outcomes, and study design (PICOS).	8
**METHODS**			
Protocol and registration	5	Indicate if a review protocol exists, if and where it can be accessed (e.g., Web address), and, if available, provide registration information including registration number.	9
Eligibility criteria	6	Specify study characteristics (e.g., PICOS, length of follow-up) and report characteristics (e.g., years considered, language, publication status) used as criteria for eligibility, giving rationale.	9
Information sources	7	Describe all information sources (e.g., databases with dates of coverage, contact with study authors to identify additional studies) in the search and date last searched.	9-10
Search	8	Present full electronic search strategy for at least one database, including any limits used, such that it could be repeated.	Appendix, p6-7
Study selection	9	State the process for selecting studies (i.e., screening, eligibility, included in systematic review, and, if applicable, included in the meta-analysis).	10
Data collection process	10	Describe method of data extraction from reports (e.g., piloted forms, independently, in duplicate) and any processes for obtaining and confirming data from investigators.	10
Data items	11	List and define all variables for which data were sought (e.g., PICOS, funding sources) and any assumptions and simplifications made.	10-11
Risk of bias in individual studies	12	Describe methods used for assessing risk of bias of individual studies (including specification of whether this was done at the study or outcome level), and how this information is to be used in any data synthesis.	11
Summary measures	13	State the principal summary measures (e.g., risk ratio, difference in means).	10
Synthesis of results	14	Describe the methods of handling data and combining results of studies, if done, including measures of consistency (e.g., I^2) for each meta-analysis.	11

Section/topic	#	Checklist item	Reported on page #
Risk of bias across studies	15	Specify any assessment of risk of bias that may affect the cumulative evidence (e.g., publication bias, selective reporting within studies).	NA
Additional analyses	16	Describe methods of additional analyses (e.g., sensitivity or subgroup analyses, meta-regression), if done, indicating which were pre-specified.	12
**RESULTS**			
Study selection	17	Give numbers of studies screened, assessed for eligibility, and included in the review, with reasons for exclusions at each stage, ideally with a flow diagram.	14
Study characteristics	18	For each study, present characteristics for which data were extracted (e.g., study size, PICOS, follow-up period) and provide the citations.	Appendix, p17-35
Risk of bias within studies	19	Present data on risk of bias of each study and, if available, any outcome level assessment (see item 12).	Appendix, p13-29
Results of individual studies	20	For all outcomes considered (benefits or harms), present, for each study: (a) simple summary data for each intervention group (b) effect estimates and confidence intervals, ideally with a forest plot.	NA
Synthesis of results	21	Present results of each meta-analysis done, including confidence intervals and measures of consistency.	Table 2
Risk of bias across studies	22	Present results of any assessment of risk of bias across studies (see Item 15).	NA
Additional analysis	23	Give results of additional analyses, if done (e.g., sensitivity or subgroup analyses, meta-regression [see Item 16]).	15, Table 3, appendix p44-48
**DISCUSSION**			
Summary of evidence	24	Summarize the main findings including the strength of evidence for each main outcome; consider their relevance to key groups (e.g., healthcare providers, users, and policy makers).	18
Limitations	25	Discuss limitations at study and outcome level (e.g., risk of bias), and at review-level (e.g., incomplete retrieval of identified research, reporting bias).	18, 21
Conclusions	26	Provide a general interpretation of the results in the context of other evidence, and implications for future research.	18-20
**FUNDING**			
Funding	27	Describe sources of funding for the systematic review and other support (e.g., supply of data); role of funders for the systematic review.	1,2,3,13

Appendix B. GATHER checklist.

Item #	Checklist item	Reported on page #
**Objectives and funding**		
**1**	Define the indicator(s), populations (including age, sex, and geographic entities), and time period(s) for which estimates were made.	9
**2**	List the funding sources for the work.	1-2
**Data Inputs**		
For all data inputs from multiple sources that are synthesized as part of the study:		
**3**	Describe how the data were identified and how the data were accessed.	9-10
**4**	Specify the inclusion and exclusion criteria. Identify all ad-hoc exclusions.	9
**5**	Provide information on all included data sources and their main characteristics. For each data source used, report reference information or contact name/institution, population represented, data collection method, year(s) of data collection, sex and age range, diagnostic criteria or measurement method, and sample size, as relevant.	Appendix p13-29
**6**	Identify and describe any categories of input data that have potentially important biases (e.g., based on characteristics listed in item 5).	12
For data inputs that contribute to the analysis but were not synthesized as part of the study:		
**7**	Describe and give sources for any other data inputs.	NA
For all data inputs:		
**8**	Provide all data inputs in a file format from which data can be efficiently extracted (e.g., a spreadsheet rather than a PDF), including all relevant meta-data listed in item 5. For any data inputs that cannot be shared because of ethical or legal reasons, such as third-party ownership, provide a contact name or the name of the institution that retains the right to the data.	Appendix p13-29
**Data analysis**		
**9**	Provide a conceptual overview of the data analysis method. A diagram may be helpful.	11
**10**	Provide a detailed description of all steps of the analysis, including mathematical formulae. This description should cover, as relevant, data cleaning, data pre-processing, data adjustments and weighting of data sources, and mathematical or statistical model(s).	11-12
**11**	Describe how candidate models were evaluated and how the final model(s) were selected.	11-12
**12**	Provide the results of an evaluation of model performance, if done, as well as the results of any relevant sensitivity analysis.	16
**13**	Describe methods for calculating uncertainty of the estimates. State which sources of uncertainty were, and were not, accounted for in the uncertainty analysis.	NA
**14**	State how analytic or statistical source code used to generate estimates can be accessed.	NA
**Results and Discussion**		
**15**	Provide published estimates in a file format from which data can be efficiently extracted.	Table 2
**16**	Report a quantitative measure of the uncertainty of the estimates (e.g. uncertainty intervals).	Table 2
**17**	Interpret results in light of existing evidence. If updating a previous set of estimates, describe the reasons for changes in estimates.	18-20
**18**	Discuss limitations of the estimates. Include a discussion of any modelling assumptions or data limitations that affect interpretation of the estimates.	21

Appendix C. Additional literature search.

The literature search for the original systematic review was conducted in April 2022. Further detail regarding this search is available elsewhere.[1] We performed an additional search in April 2024 to locate new data. The updated search covered both published literature (using databases PubMed, Embase, and PsycINFO) and grey literature. We retained the initial search domain pertaining to the population of interest (i.e., people who inject drugs) and added a search domain pertaining to sexually transmitted infection prevalence and testing coverage.

<u>Published literature search strategy</u>

PubMed

1	IDU OR "IDUs" OR PWID OR "injecting drug*" OR "intravenous drug*" OR "injecting substance*" OR "intravenous substance*" OR "people who inject*" OR "injection drug*" OR substance abuse, intravenous[Mesh]
2	"Sexually Transmitted Diseases"[Mesh] OR “sexually transmitted infection*” OR “sexually transmitted disease*” OR “sexual health” OR “venereal disease*” OR “venereal infection*” OR “Chlamydia trachomatis”[Mesh] OR “chlamydia” OR "Lymphogranuloma Venereum"[Mesh] OR "Lymphogranuloma Venereum" OR “LGV” OR "Herpes Genitalis"[Mesh] OR “genital herpes” OR “HSV-2” OR "Gonorrhea"[Mesh] OR “gonorrhea” OR “gonorrhoea” OR “gonorrhoea” OR “Neisseria gonorrhoeae” OR “Neisseria gonorrheae” OR “Syphilis”[Mesh] OR “syphilis” OR “Treponema pallidum” OR "Trichomonas Vaginitis"[Mesh] OR “trichomoniasis” OR “trichomonas vaginalis” OR "Vaginosis, Bacterial"[Mesh] OR “bacterial vaginosis” OR "Granuloma Inguinale"[Mesh] OR “granuloma inguinale” OR “donovanosis” OR “Human Papillomavirus Viruses”[Mesh] OR “human papillomavirus*” OR “HPV” OR “genital wart*”
**3**	**#1 AND #2**

Embase (OVID)

1	IDU OR IDUs OR PWID OR "injecting drug*" OR "injecting substance*" OR "people who inject*" OR "injection drug*" OR **exp intravenous drug abuse/** OR **exp intravenous drug administration/**
2	**exp sexually transmitted disease/** OR “sexually transmitted infection*” OR “sexually transmitted disease*” OR “sexual health” OR “venereal disease*” OR “venereal infection*” OR **exp Chlamydia infection**/ OR “chlamydia” OR **exp lymphogranuloma venereum**/ OR "Lymphogranuloma Venereum" OR “LGV” OR **exp genital herpes/** OR “genital herpes” OR “HSV-2” OR **exp gonorrhea/** OR “gonorrhoea” OR “gonorrhea” OR “Neisseria gonorrhoeae” OR “Neisseria gonorrheae” OR **exp syphilis/** OR “syphilis” OR “Treponema pallidum” OR **exp trichomoniasis/** OR “trichomoniasis” OR “trichomonas vaginalis” OR “trichomonas vaginitis” OR **exp bacterial vaginosis/** OR “bacterial vaginosis” OR **exp granuloma inguinale/** OR “donovanosis” OR “granuloma inguinale” OR **exp Wart virus/** OR “human papillomavirus*” OR “HPV” OR “genital wart*”
**3**	**1 AND 2**

Notes. Keywords are noted in regular type, EMTREE terms in bold.

PsycINFO (OVID)

1	IDU OR IDUs OR PWID OR "injecting drug*" OR "injecting substance*" OR "people who inject*" OR "injection drug*" OR **exp intravenous drug usage/**
3	**exp Sexually Transmitted Diseases/** OR “sexually transmitted infection*” OR “sexually transmitted disease*” OR “sexual health” OR “venereal disease*” OR “venereal infection*” OR “chlamydia” OR "Lymphogranuloma Venereum" OR “LGV” OR **exp Herpes Genitalis/** OR “genital herpes” OR “HSV-2” OR **exp Gonorrhea/** OR “gonorrhoea” OR “gonorrhea” OR “Neisseria gonorrhoeae” OR “Neisseria gonorrheae” OR **exp syphilis/** OR “syphilis” OR “Treponema pallidum” OR “trichomoniasis” OR “trichomonas vaginalis” OR “trichomonas vaginitis” OR “bacterial vaginosis” OR “donovanosis” OR “granuloma inguinale” OR **exp Human Papillomavirus/** OR “human papillomavirus*” OR “HPV” OR “genital wart*”
**3**	**1 AND 2**

Notes. Keywords are noted in regular type, index terms in bold.

Grey literature search strategy

We followed the strategy outlined previously,[1] adding sexually transmitted infection search terms. We searched websites that previously had relevant results for people who inject drugs. We also located new grey literature reports through contact with international organisations.

We used the ‘Google Advanced Search’ option as follows:

("people who inject drugs" OR "injecting drug use" OR "injection drug use" OR inject OR prevalence OR "drug use" OR "harm reduction" OR "targeted information" OR "targeted info"OR IDU OR PWID) AND ("sexually transmitted infection" OR "syphilis" OR "chlamydia" OR "gonorrhoea" OR "gonorrhea" OR "HPV" or "herpes") site:XXX

We searched the following websites:

- https://www.unodc.org
- https://www.who.int
- https://www.iasociety.org/
- https://www.opensocietyfoundations.org/voices/topics/harm-reduction
- https://www.who.int/westernpacific
- https://www.moh.govt.nz/
- https://www.nsi.bg/en/content/37/basic-page/south-america
- https://www.cicad.oas.org/Main/default_ENG.asp
- https://bjs.ojp.gov/
- https://carpha.org/
- https://www.afro.who.int/
- https://www.moh.gov.sa/Pages/Default.aspx
- http://www.euro.who.int/en/home
- https://www.emro.who.int/index.html
- https://www.moh.am/
- https://mzd.gov.cz/
- https://www.pzh.gov.pl/
- http://www.ccm.md/
- https://ghrn.ge/index.php?lang=eng
- https://www.publichealthscotland.scot/
- https://www.sciensano.be/en

Appendix D. Syphilis diagnostic test correction factors used to standardise syphilis prevalence estimates.

Diagnostic test	Correction factor
Treponemal and non-treponemal positive	1.0
Treponemal and non-treponemal positive, with rapid plasma regain ≥1:8 titre	2.5
Treponemal positive without non-treponemal confirmation	0.53
Non-treponemal positive without treponemal confirmation	0.53
Rapid test	0.70
Unknown	0.75

Notes. Table adapted from the World Health Organization [2] When a study used more than one type of test, we used the estimate derived from a combination of treponemal and non-treponemal tests.

Appendix E. Country-level socioeconomic, development and health characteristic values (grouped by UNAIDS region)

Country	Syphilis prevalence among general population (%)[1]	Injecting drug use prevalence (%)[2]	HIV prevalence among people who inject drugs (%)[2]	OAT coverage (no. people accessing OAT per person who injects drugs)[3]	NSP coverage (no. needles per person who inject drugs distributed per year)[3]	Human development index[4]	Gender inequality index[5]	Gini coefficient[6]	Country income level[7]
Australasia									
Australia	0.002275	0.6	1.3	54	508	0.946	0.063	34.3	High
Central Asia									
Kyrgyzstan	0.001471	0.68	12.4	5	68	0.701	0.345	40.9	Lower middle
Tajikistan	0.001763	0.46	18	3	250	0.679	0.269	34	Lower middle
East & Southeast Asia									
Cambodia	0.001407	0.04	8	6	146	0.6	0.486	NA	Lower middle
China	0.00397	0.25	11.9	7	4	0.788	0.186	37.1	Upper middle
Indonesia	0.003547	0.11	39.1	1	2	0.713	0.439	37.9	Upper middle
Myanmar	0.011082	0.26	26.4	22	339	0.608	0.479	30.7	Lower middle
Philippines	0.004718	0.03	6.2	NK	NK	0.710	0.388	40.7	Lower middle
Taiwan, Province of China	0.003675	NA	13.4	NC	NC	NA	NA	NA	High
Thailand	0.004988	0.11	22.2	10	11	0.803	0.310	35.1	Upper middle
Eastern Europe									
Armenia	0.001627	0.45	1.1	6	57	0.786	0.198	27.9	Upper middle
Azerbaijan	0.002049	0.61	9.8	0.5	26	0.76	0.329	26.6	Upper middle
Belarus	0.001472	1.25	25.2	1	37	0.801	0.096	31.8	Upper middle
Bosnia and Herzegovina	0.001392	0.47	0.1	13	97	0.779	0.148	33	Upper middle
Czech Republic	0.001357	0.59	0.3	12	183	0.895	0.113	26.2	High
Estonia	0.001599	0.82	51.4	15	238	0.899	0.093	30.7	High

Country	Syphilis prevalence among general population (%)[1]	Injecting drug use prevalence (%)[2]	HIV prevalence among people who inject drugs (%)[2]	OAT coverage (no. people accessing OAT per person who injects drugs)[3]	NSP coverage (no. needles per person who inject drugs distributed per year)[3]	Human development index[4]	Gender inequality index[5]	Gini coefficient[6]	Country income level[7]
Georgia	0.001916	4.19	1.6	2	34	0.814	0.283	34.2	Upper middle
Latvia	0.001568	0.6	16.2	10	135	0.879	0.142	40.9	High
Lithuania	0.001547	0.47	8.3	13	30	0.879	0.098	40.9	High
Moldova (Republic of)	0.002429	0.4	28.3	5	229	0.763	0.156	25.7	Upper middle
Poland	0.001585	NK	15.4	NE	NC	0.881	0.105	28.8	High
Russia	0.001939	1.32	49.8	NK	3	0.821	0.178	36	Upper middle
Ukraine	0.00175	1.01	20.4	4	63	0.734	0.188	25.6	Lower middle
Latin America									
Mexico	0.002855	0.13	15.8	9	5	0.781	0.352	45.4	Upper middle
Nicaragua	0.003259	NA	0	NK	NK	0.669	0.397	46.2	Lower middle
North America									
Canada	0.001787	0.7	5.8	65	286	0.935	0.069	31.7	High
United States	0.002778	1.46	5.6	18	28	0.927	0.180	39.8	High
South Asia									
Afghanistan	0.003474	0.37	3.8	0.5	NE	0.462	0.665	NA	Low
Bangladesh	0.008144	0.07	2.5	1	33	0.67	0.498	31.8	Lower middle
India	0.007036	0.09	13.6	3	34	0.644	0.437	34.2	Lower middle
Iran	0.001984	0.31	4.3	GTP	37	0.780	0.484	40.9	Lower middle
Maldives	0.00393	0.6	0	4	NK	0.762	0.328	29.3	Upper middle
Nepal	0.008731	0.2	4.6	2	68	0.601	0.495	32.8	Lower middle
Pakistan	0.008514	0.37	30.9	NK	12	0.540	0.522	29.6	Lower middle
Sri Lanka	0.000819	0.02	0	NK	NK	0.780	0.376	37.7	Lower middle
Sub-Saharan Africa									

Country	Syphilis prevalence among general population (%)[1]	Injecting drug use prevalence (%)[2]	HIV prevalence among people who inject drugs (%)[2]	OAT coverage (no. people accessing OAT per person who injects drugs)[3]	NSP coverage (no. needles per person who inject drugs distributed per year)[3]	Human development index[4]	Gender inequality index[5]	Gini coefficient[6]	Country income level[7]
Burundi	0.010147	NK	10.2	NK	NK	0.420	0.499	37.5	Low
Comoros	0.01588	NK	NK	NK	NK	0.586	NA	45.3	Lower middle
Côte d'Ivoire	0.013258	0.01	5.3	NE	NK	0.534	0.612	37.2	Lower middle
Democratic Republic of the Congo	0.031155	0.08	NA	NK	NK	0.481	0.605	42.1	Low
Ethiopia	0.008409	0.21	6.3	NK	NK	0.492	0.494	35	Low
Kenya	0.013223	0.11	11.3	3	8	0.601	0.533	40.9	Lower middle
Liberia	0.033418	0.39	NA	NK	NK	0.487	0.656	40.9	Low
Madagascar	0.027358	0.12	4.5	NE	0	0.487	0.574	42.6	Low
Mauritius	0.004351	1.31	32.3	47	80	0.796	0.369	36.8	Upper middle
Nigeria	0.013153	0.16	3.8	NK	NK	0.548	0.677	35.1	Lower middle
Seychelles	0.003171	3.05	12.6	GTP	NE	0.802	NA	32.1	High
Tanzania	0.01781	1.24	14	1	1	0.532	0.513	40.5	Lower middle
Western Europe									
Albania	0.001403	0.36	0.5	7	0	0.789	0.116	29.4	Upper middle
Germany	0.002036	0.24	4.1	62	32	0.950	0.071	31.7	High
Macedonia (TFYR)	0.011269	0.46	0	29	69	0.765	0.134	40.9	Upper middle
Serbia	0.001352	0.49	0	15	47	0.805	0.119	35	Upper middle

Abbreviations. OAT = opioid agonist treatment. NSP = needle syringe program.

Data sources for Appendix E.

[1]Global Burden of Disease, available at https://vizhub.healthdata.org/gbd-results/.

[2] Degenhardt (2023). Epidemiology of injecting drug use, prevalence of injecting-related harm, and exposure to behavioural and environmental risks among people who inject drugs: a systematic review. Lancet Glob Health. https://doi.org/10.1016/S2214-109X(23)00057-8.
[3] Colledge-Frisby (2023). Global coverage of interventions to prevent and manage drug-related harms among people who inject drugs: a systematic review. Lancet Glob Health. https://doi.org/10.1016/S2214-109X(23)00058-X.
[4] United Nations Development Program, available at https://hdr.undp.org/data-center/human-development-index#/indicies/HDI.
[5] United Nations Development Program, available at https://hdr.undp.org/data-center/thematic-composite-indices/gender-inequality-index#/indicies/GII.
[6] World Bank, available at https://data.worldbank.org/indicator/SI.POV.GINI.
[7] World Bank, available at https://datatopics.worldbank.org/world-development-indicators/the-world-by-income-and-region.html.

Appendix F. Study-level information for syphilis prevalence estimates among people who inject drugs.

Country and reference	Study year	Income level	Geographic coverage	Location (if not national)	Literature grade	Method grade	Recruitment method	Recency of injecting drug use	Syphilis diagnostic method	No. syphilis tested (N)	No. syphilis positive (n)	Crude prev. est. (%)	Adj. prev. est. (%)
Australasia													
Australia[3]	2024	High	City	Sydney	C	C	Convenience	Unspecified	Rapid test	128	3	2	2
Central Asia													
Kyrgyzstan[3]	2013	Lower middle	National		B2	B1	RDS	12 months	Treponemal positive without non-treponemal confirmation	904	76	8	5
Tajikistan[4]	2018	Lower middle	National		B3	A	RDS	6 months	Treponemal and non-treponemal positive	1916	13	0.7	0.5
Tajikistan[5]	2004	Lower middle	City	Dushanbe	A1	B1	Convenience	1 month	Treponemal and non-treponemal positive	488	77	16	16
East and Southeast Asia													
Cambodia[6]	2017	Lower middle	National		A1	A	RDS	12 months	Rapid test	310	16	5	4
Cambodia[7]	2017	Lower middle	National		A1	A	RDS	12 months	Rapid test	310	16	5	4
Cambodia[8]	2017	Lower middle	National		B3	B1	RDS	12 months	Rapid test	310	16	5	4
China[9]	2017	Upper middle	City	Chongqing	A1	B1	Convenience	12 months	Treponemal and non-treponemal positive	1716	91	5	5
China[9]	2015	Upper middle	City	Chongqing	A1	B1	Convenience	12 months	Treponemal and non-treponemal positive	2267	81	4	4
China[10]	2015	Upper middle	Sub-National	Yi Prefecture	A1	B1	Convenience	Lifetime	Rapid test	89	4	5	3
China[11]	2013	Upper middle	City	Shaoguan	A1	C	Convenience	Unspecified	Treponemal positive without non-treponemal confirmation	183	13	7	4
China[9]	2013	Upper middle	City	Chongqing	A1	B1	Convenience	12 months	Treponemal and non-treponemal positive	2004	93	5	5
China[9]	2011	Upper middle	City	Chongqing	A1	B1	Convenience	12 months	Treponemal and non-treponemal positive	2184	117	5	5
China[12]	2010	Upper middle	City	Gansu	A1	C	Convenience	Unspecified	Treponemal positive without non-treponemal confirmation	251	31	12	7
China[13]	2009	Upper middle	City	Liuzhou	A1	C	Convenience	Unspecified	Treponemal positive without non-treponemal confirmation	479	17	4	2
China[14]	2009	Upper middle	City	Jinchang	A1	C	Convenience	Unspecified	Treponemal positive without non-treponemal confirmation	97	5	5	3
China[15]	2009	Upper middle	City	Guizhou	A1	C	Convenience	Unspecified	Treponemal positive without non-treponemal confirmation	170	32	19	10

Country and reference	Study year	Income level	Geographic coverage	Location (if not national)	Literature grade	Method grade	Recruitment method	Recency of injecting drug use	Syphilis diagnostic method	No. syphilis tested (N)	No. syphilis positive (n)	Crude prev. est. (%)	Adj. prev. est. (%)
China[16]	2009	Upper middle	City	Guizhou	A1	C	Convenience	Unspecified	Treponemal positive without non-treponemal confirmation	99	20	20	11
China[17]	2009	Upper middle	City	Shenzhen	A1	C	Convenience	Unspecified	Treponemal positive without non-treponemal confirmation	195	4	2	1
China[18]	2008	Upper middle	Sub-National	Liangshan and Panzhihua Prefectures	A1	A	Convenience	3 months	Treponemal and non-treponemal positive	2530	144	6	6
China[19]	2008	Upper middle	City	Qingyuan	A1	B1	Convenience	12 months	Unknown	477	75	16	12
China[20]	2008	Upper middle	City	Nanning	A1	C	Snowball	Unspecified	Treponemal and non-treponemal positive	200	20	10	10
China[21]	2008	Upper middle	City	Mianyang	A1	C	Convenience	Unspecified	Treponemal positive without non-treponemal confirmation	651	52	8	4
China[20]	2007	Upper middle	City	Nanning	A1	C	Snowball	Unspecified	Treponemal and non-treponemal positive	200	10	5	5
China[22]	2007	Upper middle	City	Liuzhou	A1	C	Convenience	Unspecified	Treponemal positive without non-treponemal confirmation	304	21	7	4
China[23]	2007	Upper middle	City	Yueyang	A1	C	Convenience	Unspecified	Treponemal positive without non-treponemal confirmation	306	18	6	3
China[24]	2005	Upper middle	Sub-National	Guangxi Nanning, Xinjian Yili, Yunnan Honghe	A1	A	Snowball	3 months	Treponemal and non-treponemal positive	647	33	5	5
Indonesia[25]	2013	Upper middle	City	Medan	B3	B1	RDS	12 months	Treponemal and non-treponemal positive	55	4	7	7
Indonesia[25]	2013	Upper middle	City	Jakarta	B3	B1	RDS	12 months	Treponemal and non-treponemal positive	240	9	4	4
Indonesia[25]	2013	Upper middle	City	Bandung	B3	B1	RDS	12 months	Treponemal and non-treponemal positive	240	3	1	1
Indonesia[25]	2013	Upper middle	City	Malang	B3	B1	RDS	12 months	Treponemal and non-treponemal positive	203	0	0	0
Indonesia[25]	2013	Upper middle	City	Surabaya	B3	B1	RDS	12 months	Treponemal and non-treponemal positive	240	0	0	0
Indonesia[26]	2007	Upper middle	City	Medan	A1	B1	Probability	Unspecified	Treponemal positive without non-treponemal confirmation	250	6	2	1
Indonesia[26]	2007	Upper middle	City	Jakarta	A1	B1	Probability	Unspecified	Treponemal positive without non-treponemal confirmation	242	2	1	0

Country and reference	Study year	Income level	Geographic coverage	Location (if not national)	Literature grade	Method grade	Recruitment method	Recency of injecting drug use	Syphilis diagnostic method	No. syphilis tested (N)	No. syphilis positive (n)	Crude prev. est. (%)	Adj. prev. est. (%)
Indonesia[26]	2007	Upper middle	City	Bandung	A1	B1	Probability	Unspecified	Treponemal positive without non-treponemal confirmation	250	0	0	0
Indonesia[26]	2007	Upper middle	City	Surabaya	A1	B1	Probability	Unspecified	Treponemal positive without non-treponemal confirmation	250	4	2	1
Myanmar[27]	2018	Lower middle	City	Bamaw	B3	A	RDS	1 month	Rapid test	354	0	0	0
Myanmar[27]	2018	Lower middle	City	Hpakant	B3	A	RDS	1 month	Rapid test	657	7	1	1
Myanmar[27]	2018	Lower middle	City	Indaw	B3	A	RDS	1 month	Rapid test	356	2	1	<1
Myanmar[27]	2018	Lower middle	City	Kalay	B3	A	RDS	1 month	Rapid test	209	0	0	0
Myanmar[27]	2018	Lower middle	City	Kutkai	B3	A	RDS	1 month	Rapid test	407	4	1	1
Myanmar[27]	2018	Lower middle	City	Lashio	B3	A	RDS	1 month	Rapid test	604	6	1	1
Myanmar[27]	2018	Lower middle	City	Mandalay	B3	A	RDS	1 month	Rapid test	856	26	3	2
Myanmar[27]	2018	Lower middle	City	Mohynyin	B3	A	RDS	1 month	Rapid test	554	5	1	1
Myanmar[27]	2018	Lower middle	City	Muse	B3	A	RDS	1 month	Rapid test	442	11	2	2
Myanmar[27]	2018	Lower middle	City	Myitkyina	B3	A	RDS	1 month	Rapid test	462	4	1	1
Myanmar[27]	2018	Lower middle	City	Tamu	B3	A	RDS	1 month	Rapid test	254	7	3	2
Myanmar[27]	2018	Lower middle	City	Waimaw	B3	A	RDS	1 month	Rapid test	454	1	<1	<1
Myanmar[28]	2014	Lower middle	Sub-National	Yangon	B2	A	Convenience	Unspecified	Non-treponemal positive without treponemal confirmation	200	21	11	6
Myanmar[28]	2014	Lower middle	Sub-National	Mandalay	B2	A	Convenience	Unspecified	Non-treponemal positive without treponemal confirmation	200	4	2	1
Myanmar[28]	2014	Lower middle	Sub-National	Lashio	B2	A	Convenience	Unspecified	Non-treponemal positive without treponemal confirmation	200	0	0	0
Myanmar[28]	2014	Lower middle	Sub-National	Tachileik	B2	A	Convenience	Unspecified	Non-treponemal positive without treponemal confirmation	87	3	4	2
Myanmar[28]	2014	Lower middle	Sub-National	Muse	B2	A	Convenience	Unspecified	Non-treponemal positive without treponemal confirmation	100	0	0	0

Country and reference	Study year	Income level	Geographic coverage	Location (if not national)	Literature grade	Method grade	Recruitment method	Recency of injecting drug use	Syphilis diagnostic method	No. syphilis tested (N)	No. syphilis positive (n)	Crude prev. est. (%)	Adj. prev. est. (%)
Myanmar[28]	2014	Lower middle	Sub-National	Myitkyina	B2	A	Convenience	Unspecified	Non-treponemal positive without treponemal confirmation	200	0	0	0
Myanmar[29]	2012	Lower middle	Sub-National	Yangon, Mandalay, Taunggyi, Lashio, Tachileik, Muse, and Myitkyina	B3	B1	Probability	Unspecified	Non-treponemal positive without treponemal confirmation	1217	16	1	1
Myanmar[30]	2011	Lower middle	Sub-National	Yangon	B3	B1	Probability	Unspecified	Non-treponemal positive without treponemal confirmation	200	7	4	2
Myanmar[30]	2011	Lower middle	Sub-National	Mandalay	B3	B1	Probability	Unspecified	Non-treponemal positive without treponemal confirmation	200	1	1	0
Myanmar[30]	2011	Lower middle	Sub-National	Taunggyi	B3	B1	Probability	Unspecified	Non-treponemal positive without treponemal confirmation	100	4	4	2
Myanmar[30]	2011	Lower middle	Sub-National	Lashio	B3	B1	Probability	Unspecified	Non-treponemal positive without treponemal confirmation	200	0	0	0
Myanmar[30]	2011	Lower middle	Sub-National	Muse	B3	B1	Probability	Unspecified	Non-treponemal positive without treponemal confirmation	200	0	0	0
Myanmar[30]	2011	Lower middle	Sub-National	Myitkyeena	B3	B1	Probability	Unspecified	Non-treponemal positive without treponemal confirmation	200	0	0	0
Myanmar[31]	2010	Lower middle	Sub-National	Yangon, Mandalay, Taunggyi, Lashio, Muse and Myitkyina	B3	B1	Probability	Unspecified	Non-treponemal positive without treponemal confirmation	1029	4	0	0
Myanmar[32]	2009	Lower middle	Sub-National	Yangon	B3	B1	Unspecified	Unspecified	Non-treponemal positive without treponemal confirmation	200	11	6	3
Myanmar[32]	2009	Lower middle	Sub-National	Mandalay	B3	B1	Unspecified	Unspecified	Non-treponemal positive without treponemal confirmation	200	2	1	1
Myanmar[32]	2009	Lower middle	Sub-National	Lashio	B3	B1	Unspecified	Unspecified	Non-treponemal positive without treponemal confirmation	200	1	1	0
Myanmar[32]	2009	Lower middle	Sub-National	Muse	B3	B1	Unspecified	Unspecified	Non-treponemal positive without treponemal confirmation	150	0	0	0

Country and reference	Study year	Income level	Geographic coverage	Location (if not national)	Literature grade	Method grade	Recruitment method	Recency of injecting drug use	Syphilis diagnostic method	No. syphilis tested (N)	No. syphilis positive (n)	Crude prev. est. (%)	Adj. prev. est. (%)
Myanmar[32]	2009	Lower middle	Sub-National	Myitkyina	B3	B1	Unspecified	Unspecified	Non-treponemal positive without treponemal confirmation	195	0	0	0
Myanmar[33]	2008	Lower middle	Sub-National	Yangon	B3	B1	Convenience	Unspecified	Non-treponemal positive without treponemal confirmation	200	20	10	5
Myanmar[33]	2008	Lower middle	Sub-National	Mandalay	B3	B1	Convenience	Unspecified	Non-treponemal positive without treponemal confirmation	154	1	1	0
Myanmar[33]	2008	Lower middle	Sub-National	Lashio	B3	B1	Convenience	Unspecified	Non-treponemal positive without treponemal confirmation	171	0	0	0
Myanmar[33]	2008	Lower middle	Sub-National	Muse	B3	B1	Convenience	Unspecified	Non-treponemal positive without treponemal confirmation	150	2	1	1
Myanmar[33]	2008	Lower middle	Sub-National	Myitkyina	B3	B1	Convenience	Unspecified	Non-treponemal positive without treponemal confirmation	200	3	2	1
Philippines[34]	2013	Lower middle	National		B2	B1	RDS	Unspecified	Unknown	767	37	5	4
Philippines[35]	2011	Lower middle	Sub-National	Cebu	B3	B1	RDS	6 months	Unknown	306	12	4	3
Philippines[35]	2011	Lower middle	Sub-National	Gen Santos	B3	B1	RDS	6 months	Unknown	366	0	0	0
Philippines[35]	2011	Lower middle	Sub-National	Zamboanga	B3	B1	RDS	6 months	Unknown	300	0	0	0
Philippines[35]	2011	Lower middle	Sub-National	Mandaue	B3	B1	RDS	6 months	Unknown	311	12	4	3
Taiwan[36]	2011	High	National		C	C	Convenience	Unspecified	Unknown	87	17	20	15
Thailand[37]	2012	Upper middle	Sub-National	Bangkok	B3	B1	RDS	6 months	Unknown	321	2	1	1
Thailand[37]	2012	Upper middle	Sub-National	Chiang Mai	B3	B1	RDS	6 months	Unknown	274	16	6	4
Thailand[37]	2012	Upper middle	Sub-National	Songkhla	B3	B1	RDS	6 months	Unknown	173	3	2	1
Eastern Europe													
Armenia[38]	2021	Upper middle	City	Yerevan	B2	A	RDS	3 months	Rapid test	300	0	0	0
Armenia[38]	2021	Upper middle	City	Gyumri	B2	A	RDS	3 months	Rapid test	150	2	1	1
Armenia[38]	2021	Upper middle	City	Vanadzor	B2	A	RDS	3 months	Rapid test	150	2	1	1
Armenia[39]	2018	Upper middle	City	Yerevan	B3	B1	Unspecified	3 months	Unknown	300	10	3	3

Country and reference	Study year	Income level	Geographic coverage	Location (if not national)	Literature grade	Method grade	Recruitment method	Recency of injecting drug use	Syphilis diagnostic method	No. syphilis tested (N)	No. syphilis positive (n)	Crude prev. est. (%)	Adj. prev. est. (%)
Armenia[39]	2018	Upper middle	City	Gyumri	B3	B1	Unspecified	1 month	Unknown	150	0	0	0
Armenia[39]	2018	Upper middle	City	Vanadzor	B3	B1	Unspecified	1 month	Unknown	150	0	0	0
Armenia[40]	2012	Upper middle	City	Yerevan	B2	B1	RDS	3 months	Treponemal and non-treponemal positive	300	2	1	1
Armenia[40]	2012	Upper middle	City	Gyumri	B2	B1	RDS	3 months	Treponemal and non-treponemal positive	50	0	0	0
Armenia[40]	2012	Upper middle	City	Vanadzor	B2	B1	RDS	3 months	Treponemal and non-treponemal positive	50	0	0	0
Armenia[41]	2011	Upper middle	City	Yerevan	B3	B1	RDS	3 months	Treponemal and non-treponemal positive	270	11	4	4
Azerbaijan[42]	2012	Upper middle	City	Baku	B2	B2	Convenience	12 months	Unknown	300	17	6	4
Azerbaijan[42]	2012	Upper middle	City	Lankaran	B2	B2	Convenience	12 months	Unknown	150	7	5	4
Azerbaijan[42]	2012	Upper middle	City	Sumqayit	B2	B2	Convenience	12 months	Unknown	150	8	5	4
Azerbaijan[42]	2012	Upper middle	City	Hajigabul	B2	B2	Convenience	12 months	Unknown	150	22	15	11
Azerbaijan[42]	2012	Upper middle	City	Shirvan	B2	B2	Convenience	12 months	Unknown	150	10	7	5
Azerbaijan[42]	2012	Upper middle	City	Ganja	B2	B2	Convenience	12 months	Unknown	150	9	6	5
Azerbaijan[42]	2012	Upper middle	City	Masalli	B2	B2	Convenience	12 months	Unknown	150	11	7	6
Azerbaijan[43]	2008	Upper middle	City	Baku	B3	B1	Convenience	1 month	Treponemal positive without non-treponemal confirmation	200	7	4	2
Azerbaijan[43]	2008	Upper middle	City	Sumgait	B3	B1	Convenience	1 month	Treponemal positive without non-treponemal confirmation	150	8	5	3
Azerbaijan[43]	2008	Upper middle	City	Gandja	B3	B1	Convenience	1 month	Treponemal positive without non-treponemal confirmation	150	5	3	2
Azerbaijan[43]	2008	Upper middle	City	Lenkoran Rayon	B3	B1	Convenience	1 month	Treponemal positive without non-treponemal confirmation	150	5	3	2
Azerbaijan[43]	2008	Upper middle	City	Masalli	B3	B1	Convenience	1 month	Treponemal positive without non-treponemal confirmation	150	16	11	6
Azerbaijan[43]	2008	Upper middle	City	Ali-Bayramli	B3	B1	Convenience	1 month	Treponemal positive without non-treponemal confirmation	100	8	8	4

Country and reference	Study year	Income level	Geographic coverage	Location (if not national)	Literature grade	Method grade	Recruitment method	Recency of injecting drug use	Syphilis diagnostic method	No. syphilis tested (N)	No. syphilis positive (n)	Crude prev. est. (%)	Adj. prev. est. (%)
Azerbaijan[43]	2008	Upper middle	City	Hajikabul	B3	B1	Convenience	1 month	Treponemal positive without non-treponemal confirmation	100	10	10	5
Belarus[44]	2015	Upper middle	City	Gomel	B2	B1	RDS	Unspecified	Unknown	360	1	0	0
Belarus[44]	2015	Upper middle	City	Minsk	B2	B1	RDS	Unspecified	Unknown	400	0	0	0
Belarus[44]	2015	Upper middle	City	Pinsk	B2	B1	RDS	Unspecified	Unknown	283	7	3	2
Belarus[44]	2015	Upper middle	City	Polotsk	B2	B1	RDS	Unspecified	Unknown	215	0	0	0
Belarus[44]	2015	Upper middle	City	Svetlogorsk	B2	B1	RDS	Unspecified	Unknown	160	0	0	0
Belarus[44]	2015	Upper middle	City	Soligorsk	B2	B1	RDS	Unspecified	Unknown	235	0	0	0
Bosnia and Herzegovina[45]	2012	Upper middle	City	Sarajevo	B3	B1	RDS	1 month	Unknown	200	0	0	0
Bosnia and Herzegovina[45]	2012	Upper middle	City	Zenica	B3	B1	RDS	1 month	Unknown	209	0	0	0
Bosnia and Herzegovina[45]	2012	Upper middle	City	Mostar	B3	B1	RDS	1 month	Unknown	200	1	1	0
Bosnia and Herzegovina[45]	2012	Upper middle	City	Bejeljina	B3	B1	RDS	1 month	Unknown	129	1	1	1
Bosnia and Herzegovina[45]	2012	Upper middle	City	Banja Luka	B3	B1	RDS	1 month	Unknown	260	2	1	1
Czech Republic[46]	2018	High	Subnational	Pilsen Region	A1	B1	Unspecified	Lifetime	Unknown	384	7	2	1
Estonia[47]	2007	High	City	Tallinn	A1	B1	RDS	Unspecified	Non-treponemal positive without treponemal confirmation	350	33	9	5
Georgia[48]	2009	Upper middle	City	Tbilisi	B3	A	Unspecified	1 month	Treponemal positive without non-treponemal confirmation	306	19	6	3
Georgia[48]	2009	Upper middle	City	Batumi	B3	A	Unspecified	1 month	Treponemal positive without non-treponemal confirmation	206	15	7	4
Georgia[48]	2009	Upper middle	City	Zugdidi	B3	A	Unspecified	1 month	Treponemal positive without non-treponemal confirmation	204	14	7	4
Georgia[48]	2009	Upper middle	City	Telavi	B3	A	Unspecified	1 month	Treponemal positive without non-treponemal confirmation	205	11	5	3
Georgia[48]	2009	Upper middle	City	Gori	B3	A	Unspecified	1 month	Treponemal positive without non-treponemal confirmation	187	7	4	2

Country and reference	Study year	Income level	Geographic coverage	Location (if not national)	Literature grade	Method grade	Recruitment method	Recency of injecting drug use	Syphilis diagnostic method	No. syphilis tested (N)	No. syphilis positive (n)	Crude prev. est. (%)	Adj. prev. est. (%)
Hungary[49]	2006	High	City	Budapest	A1	A	Snowball	1 month	Treponemal and non-treponemal positive	186	7	4	4
Latvia[50]	2007	High	City	Riga	B3	B1	RDS	Unspecified	Non-treponemal positive without treponemal confirmation	407	18	4	2
Lithuania[50]	2008	High	City	Vilnius	B3	B1	RDS	Unspecified	Non-treponemal positive without treponemal confirmation	400	28	7	4
Moldova (Republic of)[51]	2020	Upper middle	City	Chisinau	B2	A	RDS	12 months	Rapid test	365	13	4	3
Moldova (Republic of)[51]	2020	Upper middle	City	Balti	B2	A	RDS	12 months	Rapid test	357	19	5	4
Moldova (Republic of)[51]	2020	Upper middle	City	Tiraspol	B2	A	RDS	12 months	Rapid test	333	12	4	3
Moldova (Republic of)[51]	2020	Upper middle	City	Ribnita	B2	A	RDS	12 months	Rapid test	322	14	4	3
Moldova[52]	2013	Upper middle	City	Chisinau	B2	B2	RDS	12 months	Unknown	339	2	1	0
Moldova[52]	2013	Upper middle	City	Balti	B2	B2	RDS	12 months	Unknown	362	3	1	1
Moldova[52]	2013	Upper middle	City	Tiraspol	B2	B2	RDS	12 months	Unknown	295	24	8	6
Moldova[52]	2013	Upper middle	City	Ribnita	B2	B2	RDS	12 months	Unknown	97	8	8	6
Poland[53]	2009	High	Sub-National	Gdansk, Krakow	B2	B1	RDS	Lifetime	Non-treponemal positive without treponemal confirmation	193	1	0	0
Russia[54]	2011	Upper middle	City	Barnaul	C	B2	Unspecified	Unspecified	Unknown	300	42	14	11
Russia[55]	2003	Upper middle	Sub-National	Moscow, Volgograd, Barnaul	A1	B1	Convenience	1 month	Treponemal positive without non-treponemal confirmation	1389	152	11	6
Ukraine[56]	2020	Lower middle	National		B2	A	RDS	1 month	Rapid test	6001	144	2	2
Ukraine[57]	2015	Lower middle	National		B2	B1	RDS	1 month	Unknown	9407	334	4	3
Ukraine[58]	2009	Lower middle	City	Simferopol	B2	B1	Unspecified	1 month	Treponemal positive without non-treponemal confirmation	248	9	4	2
Ukraine[58]	2009	Lower middle	City	Mykolaiv	B2	B1	Unspecified	1 month	Treponemal positive without non-treponemal confirmation	241	36	15	8
Ukraine[58]	2009	Lower middle	City	Dnipropetrov sk	B2	B1	Unspecified	1 month	Treponemal positive without non-treponemal confirmation	244	21	9	5

Country and reference	Study year	Income level	Geographic coverage	Location (if not national)	Literature grade	Method grade	Recruitment method	Recency of injecting drug use	Syphilis diagnostic method	No. syphilis tested (N)	No. syphilis positive (n)	Crude prev. est. (%)	Adj. prev. est. (%)
Ukraine[58]	2009	Lower middle	City	Severodonetsk	B2	B1	Unspecified	1 month	Treponemal positive without non-treponemal confirmation	247	13	5	3
Ukraine[58]	2009	Lower middle	City	Kryvy Rih	B2	B1	Unspecified	1 month	Treponemal positive without non-treponemal confirmation	243	25	10	6
Ukraine[58]	2009	Lower middle	City	Zaporizhya	B2	B1	Unspecified	1 month	Treponemal positive without non-treponemal confirmation	246	13	5	3
Ukraine[58]	2009	Lower middle	City	Kyiv	B2	B1	Unspecified	1 month	Treponemal positive without non-treponemal confirmation	379	53	14	7
Ukraine[58]	2009	Lower middle	City	Vinnytsya	B2	B1	Unspecified	1 month	Treponemal positive without non-treponemal confirmation	243	22	9	5
Ukraine[58]	2009	Lower middle	City	Cherkasy	B2	B1	Unspecified	1 month	Treponemal positive without non-treponemal confirmation	245	7	3	2
Ukraine[58]	2009	Lower middle	City	Chernihiv	B2	B1	Unspecified	1 month	Treponemal positive without non-treponemal confirmation	245	2	1	0
Ukraine[58]	2009	Lower middle	City	Zhytomyr	B2	B1	Unspecified	1 month	Treponemal positive without non-treponemal confirmation	242	11	4	2
Ukraine[58]	2009	Lower middle	City	Chervonograd	B2	B1	Unspecified	1 month	Treponemal positive without non-treponemal confirmation	238	6	2	1
Ukraine[58]	2009	Lower middle	City	Rivne	B2	B1	Unspecified	1 month	Treponemal positive without non-treponemal confirmation	243	36	15	8
Ukraine[58]	2009	Lower middle	City	Ivano-Frankivsk	B2	B1	Unspecified	1 month	Treponemal positive without non-treponemal confirmation	244	34	14	7
Ukraine[58]	2009	Lower middle	City	Ternopil	B2	B1	Unspecified	1 month	Treponemal positive without non-treponemal confirmation	99	5	5	3
Ukraine[58]	2009	Lower middle	City	Uzhgorod	B2	B1	Unspecified	1 month	Treponemal positive without non-treponemal confirmation	98	3	3	2
Ukraine[58]	2009	Lower middle	City	Chernivtsi	B2	B1	Unspecified	1 month	Treponemal positive without non-treponemal confirmation	98	1	1	1
Latin America													
Mexico[59]	2007	Upper middle	City	Tijuana	A1	B1	RDS	6 months	Treponemal and non-treponemal positive	1056	76	7	7

Country and reference	Study year	Income level	Geographic coverage	Location (if not national)	Literature grade	Method grade	Recruitment method	Recency of injecting drug use	Syphilis diagnostic method	No. syphilis tested (N)	No. syphilis positive (n)	Crude prev. est. (%)	Adj. prev. est. (%)
Mexico[60]	2005	Upper middle	City	Ciudad Juárez	A1	A	RDS	1 month	Treponemal and non-treponemal positive	193	7	4	4
Mexico[61]	2005	Upper middle	City	Tijuana	A1	B1	RDS	1 month	Treponemal and non-treponemal positive	219	28	13	13
Nicaragua[62]	2020	Lower middle	National		B3	B1	Unspecified	Unspecified	Unknown	100	1	1	1
Nicaragua[63]	2014	Lower middle	National		B3	B1	Convenience	Lifetime	Treponemal and non-treponemal positive	41	2	5	5
North America													
Canada[64]	2012	High	City	Montreal	C	B1	Convenience	Unspecified	Treponemal and non-treponemal positive	109	2	2	2
United States[65]	2018	High	City	Kentucky	A2	C	Convenience	Unspecified	Unknown	71	3	4	3
United States[60]	2006	High	City	El Paso	A1	A	RDS	1 month	Treponemal and non-treponemal positive	150	8	5	5
United States[60]	2005	High	Sub-National	Doña Ana County	A1	A	RDS	1 month	Treponemal and non-treponemal positive	100	3	3	3
South Asia													
Afghanistan[66]	2012	Low	City	Kabul	B3	B1	RDS	3 months	Rapid test	368	21	6	4
Afghanistan[66]	2012	Low	City	Herat	B3	B1	RDS	3 months	Rapid test	184	5	3	2
Afghanistan[66]	2012	Low	City	Mazar-i-Shariff	B3	B1	RDS	3 months	Rapid test	254	23	7	6
Afghanistan[66]	2012	Low	City	Jalalabad	B3	B1	RDS	3 months	Rapid test	236	18	4	5
Afghanistan[66]	2012	Low	City	Charikar	B3	B1	RDS	3 months	Rapid test	117	3	4	2
Afghanistan[67]	2009	Low	City	Kabul	A1	A	Convenience	1 month	Treponemal and non-treponemal positive	483	6	1	1
Afghanistan[68]	2009	Low	City	Herat	A1	B1	RDS	1 month	Rapid test	160	3	2	1
Afghanistan[68]	2009	Low	City	Kabul	A1	B1	RDS	1 month	Rapid test	286	10	4	2
Afghanistan[68]	2009	Low	City	Mazar-i-Sharif	A1	B1	RDS	1 month	Rapid test	102	17	17	12
Afghanistan[69]	2008	Low	City	Herat	A1	B1	Convenience	6 months	Treponemal and non-treponemal positive	333	4	1	1
Afghanistan[69]	2008	Low	City	Jalalabad	A1	B1	Convenience	6 months	Treponemal and non-treponemal positive	96	0	0	0
Afghanistan[69]	2008	Low	City	Kabul	A1	B1	Convenience	6 months	Treponemal and non-treponemal positive	463	10	2	2
Afghanistan[69]	2008	Low	City	Mazar-i-Sharif	A1	B1	Convenience	6 months	Treponemal and non-treponemal positive	187	26	14	14
Bangladesh[70]	2020	Lower middle	Subnational	Narayanganj	B2	A	RDS	1 month	Treponemal and non-treponemal positive	380	27	7	7
Bangladesh[70]	2020	Lower middle	Subnational	Cumilla	B2	A	RDS	1 month	Treponemal and non-treponemal positive	381	8	2	2
Bangladesh[70]	2020	Lower middle	Subnational	Gazipur	B2	A	RDS	1 month	Treponemal and non-treponemal positive	372	21	6	6
Bangladesh[70]	2020	Lower middle	Subnational	Dhaka	B2	A	RDS	1 month	Treponemal and non-treponemal positive	652	30	5	5

Country and reference	Study year	Income level	Geographic coverage	Location (if not national)	Literature grade	Method grade	Recruitment method	Recency of injecting drug use	Syphilis diagnostic method	No. syphilis tested (N)	No. syphilis positive (n)	Crude prev. est. (%)	Adj. prev. est. (%)
Bangladesh[70]	2020	Lower middle	Subnational	Rajshahi	B2	A	RDS	1 month	Treponemal and non-treponemal positive	331	4	1	1
Bangladesh[70]	2020	Lower middle	Subnational	Chapainaw-abganj	B2	A	RDS	1 month	Treponemal and non-treponemal positive	260	5	2	2
Bangladesh[70]	2020	Lower middle	Subnational	Barishal	B2	A	Probability	1 month	Treponemal and non-treponemal positive	281	1	0	0
Bangladesh[70]	2020	Lower middle	Subnational	Mymensing-h	B2	A	Probability	1 month	Treponemal and non-treponemal positive	376	1	0	0
Bangladesh[71]	2011	Lower middle	City	Dhaka	B3	A	Convenience	12 months	Treponemal and non-treponemal positive with RPR reagin >1:8 titre	1243	53	4	11
Bangladesh[71]	2011	Lower middle	City	Mymensing-h	B3	A	Convenience	12 months	Treponemal and non-treponemal positive with RPR reagin >1:8 titre	375	8	2	5
Bangladesh[71]	2011	Lower middle	City	Narayanganj	B3	A	Convenience	12 months	Treponemal and non-treponemal positive with RPR reagin >1:8 titre	261	14	5	13
Bangladesh[71]	2011	Lower middle	City	Tongi	B3	A	Convenience	12 months	Treponemal and non-treponemal positive with RPR reagin >1:8 titre	149	5	3	8
Bangladesh[71]	2011	Lower middle	City	Norsingdi	B3	A	Convenience	12 months	Treponemal and non-treponemal positive with RPR reagin >1:8 titre	101	8	8	20
Bangladesh[71]	2011	Lower middle	City	Chandpur	B3	A	Convenience	12 months	Treponemal and non-treponemal positive with RPR reagin >1:8 titre	115	7	6	15
Bangladesh[71]	2011	Lower middle	City	Teknaf	B3	A	Convenience	12 months	Treponemal and non-treponemal positive with RPR reagin >1:8 titre	96	5	5	13
Bangladesh[71]	2011	Lower middle	City	Rajshahi	B3	A	Convenience	12 months	Treponemal and non-treponemal positive with RPR reagin >1:8 titre	401	19	3	12
Bangladesh[71]	2011	Lower middle	City	Chapai Nawabganj	B3	A	Convenience	12 months	Treponemal and non-treponemal positive with RPR reagin >1:8 titre	220	4	2	5
Bangladesh[71]	2011	Lower middle	City	Kanshat	B3	A	Convenience	12 months	Treponemal and non-treponemal positive with RPR reagin >1:8 titre	92	1	1	3
Bangladesh[71]	2011	Lower middle	City	Char Norendrapur	B3	A	Convenience	12 months	Treponemal and non-treponemal positive with RPR reagin >1:8 titre	124	1	1	2
Bangladesh[71]	2011	Lower middle	City	Rangpur	B3	A	Convenience	12 months	Treponemal and non-treponemal positive with RPR reagin >1:8 titre	103	3	3	7

Country and reference	Study year	Income level	Geographic coverage	Location (if not national)	Literature grade	Method grade	Recruitment method	Recency of injecting drug use	Syphilis diagnostic method	No. syphilis tested (N)	No. syphilis positive (n)	Crude prev. est. (%)	Adj. prev. est. (%)
Bangladesh[71]	2011	Lower middle	City	Naogaon	B3	A	Convenience	12 months	Treponemal and non-treponemal positive with RPR reagin >1:8 titre	382	3	1	2
Bangladesh[71]	2011	Lower middle	City	Pabna	B3	A	Convenience	12 months	Treponemal and non-treponemal positive with RPR reagin >1:8 titre	101	0	0	0
Bangladesh[71]	2011	Lower middle	City	Ishwardi	B3	A	Convenience	12 months	Treponemal and non-treponemal positive with RPR reagin >1:8 titre	57	3	5	13
Bangladesh[71]	2011	Lower middle	City	Sirajganj	B3	A	Convenience	12 months	Treponemal and non-treponemal positive with RPR reagin >1:8 titre	344	6	2	4
Bangladesh[71]	2011	Lower middle	City	Hili	B3	A	Convenience	12 months	Treponemal and non-treponemal positive with RPR reagin >1:8 titre	138	3	2	5
Bangladesh[71]	2011	Lower middle	City	Dinajpur	B3	A	Convenience	12 months	Treponemal and non-treponemal positive with RPR reagin >1:8 titre	385	2	1	1
Bangladesh[71]	2011	Lower middle	City	Jessore	B3	A	Convenience	12 months	Treponemal and non-treponemal positive with RPR reagin >1:8 titre	190	5	3	7
Bangladesh[71]	2011	Lower middle	City	Sathkhira	B3	A	Convenience	12 months	Treponemal and non-treponemal positive with RPR reagin >1:8 titre	285	0	0	0
Bangladesh[71]	2011	Lower middle	City	Srimongol	B3	A	Convenience	12 months	Treponemal and non-treponemal positive with RPR reagin >1:8 titre	79	2	3	6
Bangladesh[71]	2011	Lower middle	City	Barisal	B3	A	Convenience	12 months	Treponemal and non-treponemal positive with RPR reagin >1:8 titre	404	3	1	2
Bangladesh[71]	2011	Lower middle	City	Benapole	B3	A	Convenience	12 months	Treponemal and non-treponemal positive	96	0	0	0
Bangladesh[72]	2006	Lower middle	City	Dhaka	A1	A	Convenience	12 months	Treponemal and non-treponemal positive with RPR reagin >1:8 titre	4216	87	2	5
Bangladesh[73]	2003	Lower middle	City	Dhaka	A1	B1	Convenience	Unspecified	Treponemal and non-treponemal positive with RPR reagin >1:8 titre	561	19	3	9
India[74]	2012	Lower middle	Sub-National	West Bengal	A1	C	Convenience	Unspecified	Treponemal and non-treponemal positive with RPR reagin >1:8 titre	58	0	0	0
India[75]	2010	Lower middle	Sub-National	Punjab	A1	B1	Convenience	3 months	Treponemal and non-treponemal positive	1155	20	2	2
India[76]	2010	Lower middle	City	Mumbai-Thane	B2	B1	RDS	6 months	Treponemal and non-treponemal positive	327	27	8	8

Country and reference	Study year	Income level	Geographic coverage	Location (if not national)	Literature grade	Method grade	Recruitment method	Recency of injecting drug use	Syphilis diagnostic method	No. syphilis tested (N)	No. syphilis positive (n)	Crude prev. est. (%)	Adj. prev. est. (%)
India[76]	2010	Lower middle	City	Bishnupur	B2	B1	RDS	6 months	Treponemal and non-treponemal positive	410	17	4	4
India[76]	2010	Lower middle	City	Churachandpur	B2	B1	RDS	6 months	Treponemal and non-treponemal positive	411	11	3	3
India[76]	2010	Lower middle	City	Phek	B2	B1	RDS	6 months	Treponemal and non-treponemal positive	418	58	14	14
India[76]	2010	Lower middle	City	Wokha	B2	B1	RDS	6 months	Treponemal and non-treponemal positive	411	68	17	17
India[77]	2009	Lower middle	Sub-National	Manipur	A1	B1	RDS	6 months	Treponemal and non-treponemal positive	821	32	4	4
India[77]	2009	Lower middle	Sub-National	Nagaland	A1	B1	RDS	6 months	Treponemal and non-treponemal positive	829	111	13	13
India[78]	2008	Lower middle	City	Bishnupur	A1	B1	RDS	6 months	Treponemal and non-treponemal positive	420	24	6	6
India[78]	2008	Lower middle	City	Churachandpur	A1	B1	RDS	6 months	Treponemal and non-treponemal positive	419	4	1	1
India[78]	2008	Lower middle	City	Phek	A1	B1	RDS	6 months	Treponemal and non-treponemal positive	440	33	7	8
India[78]	2008	Lower middle	City	Wokha	A1	B1	RDS	6 months	Treponemal and non-treponemal positive	420	82	20	20
India[78]	2008	Lower middle	City	Mumbai/ Thane	A1	B1	RDS	6 months	Treponemal and non-treponemal positive	355	17	5	5
India[79]	2006	Lower middle	Sub-National	Manipur	A1	B1	RDS	6 months	Treponemal and non-treponemal positive	839	34	4	4
India[79]	2006	Lower middle	Sub-National	Nagaland	A1	B1	RDS	6 months	Treponemal and non-treponemal positive	821	99	12	12
Iran[80]	2007	Lower middle	City	Tehran	A1	A	Snowball	Unspecified	Non-treponemal positive without treponemal confirmation	887	4	1	0
Maldives[81]	2008	Upper middle	City	Male'	B2	B1	Snowball	6 months	Unknown	147	0	0	0
Maldives[81]	2008	Upper middle	City	Addu	B2	B1	Snowball	6 months	Unknown	129	0	0	0
Nepal[82]	2020	Lower middle	Subnational	Province 1	B2	A	RDS	6 months	Unknown	200	0	0	0
Nepal[82]	2020	Lower middle	Subnational	Province 2	B2	A	RDS	6 months	Unknown	200	0	0	0
Nepal[82]	2020	Lower middle	Subnational	Bagmati	B2	A	RDS	6 months	Unknown	470	8	2	0
Nepal[82]	2020	Lower middle	Subnational	Gandaki	B2	A	RDS	6 months	Unknown	350	8	2	1
Nepal[82]	2020	Lower middle	Subnational	Lumbini	B2	A	RDS	6 months	Unknown	250	1	0	0
Nepal [82]	2020	Lower middle	Subnational	Karnali	B2	A	RDS	6 months	Unknown	100	0	0	0

Country and reference	Study year	Income level	Geographic coverage	Location (if not national)	Literature grade	Method grade	Recruitment method	Recency of injecting drug use	Syphilis diagnostic method	No. syphilis tested (N)	No. syphilis positive (n)	Crude prev. est. (%)	Adj. prev. est. (%)
Nepal[82]	2020	Lower middle	Subnational	Sudurpasc-him	B2	A	RDS	6 months	Unknown	120	0	0	0
Nepal[82]	2020	Lower middle	Subnational	Province 1, Bagmati, Gandanki	B2	A	Convenience	6 months	Unknown	150	15	10	5
Nepal[83]	2020	Lower middle	National		B3	B2	RDS	6 months	Treponemal and non-treponemal positive	1690	20	1	1
Nepal[83]	2020	Lower middle	National		B3	B2	Convenience	6 months	Treponemal and non-treponemal positive	150	15	10	10
Nepal[84]	2017	Lower middle	Sub-National	Kathmandu, Bhaktapur and Lalitpur districts	B3	A	RDS	3 months	Treponemal and non-treponemal positive with RPR reagin >1:8 titre	340	3	1	2
Nepal[85]	2017	Lower middle	Sub-National	Pokhara Valley	B3	B1	Probability	3 months	Treponemal and non-treponemal positive	155	6	4	4
Nepal[86]	2017	Lower middle	Sub-National	West to Far West Terai Districts	B3	B2	Probability	3 months	Treponemal and non-treponemal positive with RPR reagin >1:8 titre	300	6	2	5
Nepal[87]	2016	Lower middle	City	Kathmandu	C	C	Probability	Unspecified	Non-treponemal positive without treponemal confirmation	160	13	8	4
Nepal[88]	2016	Lower middle	Sub-National	Kathmandu Valley	B3	A	Probability	3 months	Rapid test	160	12	8	5
Nepal[89]	2015	Lower middle	Sub-National	Eastern Terai	B3	B1	Probability	3 months	Treponemal and non-treponemal positive with RPR reagin >1:8 titre	360	4	1	3
Nepal[90]	2015	Lower middle	Sub-National	Kathmandu Valley	B3	B1	RDS	3 months	Treponemal and non-treponemal positive with RPR reagin >1:8 titre	340	0	0	0
Nepal[91]	2015	Lower middle	Sub-National	Pokhara Valley	B3	B1	RDS	3 months	Treponemal and non-treponemal positive with RPR reagin >1:8 titre	345	4	1	3
Nepal[92]	2012	Lower middle	Sub-National	Eastern Terai	B3	B1	Probability	3 months	Treponemal and non-treponemal positive	100	6	2	6
Nepal[93]	2011	Lower middle	Sub-National	Kathmandu Valley	B3	B1	RDS	3 months	Treponemal and non-treponemal positive with RPR reagin >1:8 titre	340	0	0	0
Nepal[94]	2009	Lower middle	Sub-National	Eastern Terai	B3	B1	Probability	3 months	Treponemal and non-treponemal positive	345	6	2	2
Nepal[95]	2009	Lower middle	Sub-National	Kathmandu Valley	B3	B1	RDS	3 months	Treponemal and non-treponemal positive	300	5	2	2
Nepal[96]	2009	Lower middle	Sub-National	Pokhara Valley	B3	B1	RDS	3 months	Treponemal and non-treponemal positive	300	2	1	1

Country and reference	Study year	Income level	Geographic coverage	Location (if not national)	Literature grade	Method grade	Recruitment method	Recency of injecting drug use	Syphilis diagnostic method	No. syphilis tested (N)	No. syphilis positive (n)	Crude prev. est. (%)	Adj. prev. est. (%)
Nepal[97]	2009	Lower middle	Sub-National	Western to Far-Western Terai	B3	B1	Probability	3 months	Treponemal and non-treponemal positive	300	5	2	2
Pakistan[98]	2007	Lower middle	City	Rawalpindi	A1	B1	RDS	1 month	Treponemal and non-treponemal positive	302	23	8	8
Pakistan[98]	2007	Lower middle	City	Abbottabad	A1	B1	RDS	1 month	Treponemal and non-treponemal positive	102	4	4	4
Sri Lanka [99]	2017	Lower middle	City	Colombo	B3	B1	RDS	Unspecified	Treponemal and non-treponemal positive with RPR reagin >1:8 titre	305	2	1	2
Sri Lanka[100]	2014	Lower middle	City	Colombo	B3	C	RDS	12 months	Treponemal and non-treponemal positive with RPR reagin >1:8 titre	326	0	0	0
Sub-Saharan Africa													
Burundi[101]	2021	Low	Subnational	Bujumbura	B2	A	RDS	6 months	Treponemal and non-treponemal positive	178	20	11	11
Burundi[101]	2021	Low	Subnational	Central East	B2	A	RDS	6 months	Treponemal and non-treponemal positive	48	7	15	15
Burundi[101]	2021	Low	Subnational	North	B2	A	RDS	6 months	Treponemal and non-treponemal positive	37	3	8	8
Burundi[101]	2021	Low	Subnational	South	B2	A	RDS	6 months	Treponemal and non-treponemal positive	30	5	17	17
Comoros[102]	2020	Lower middle	Subnational	Ngazidja, Ndzuwani, Mwali	B2	A	RDS	12 months	Unknown	88	0	0	0
Côte d'Ivoire[103]	2014	Lower middle	City	Abidjan	A1	B1	RDS	Lifetime	Treponemal and non-treponemal positive	57	0	0	0
Democratic Republic of the Congo[104]	2019	Low	National		B3	A	RDS	12 months	Treponemal and non-treponemal positive	1701	18	1	1
Ethiopia[105]	2015	Low	City	Addis Ababa	A1	B1	RDS	6 months	Treponemal and non-treponemal positive	237	12	5	5
Kenya[106]	2011	Lower middle	City	Nairobi	A1	B1	RDS	3 months	Treponemal and non-treponemal positive	269	4	2	2
Liberia[107]	2018	Low	Subnational	Montserrad-o County, Grand Cape Mount, Grand Gedeh, Margibi	B2	A	RDS	12 months	Treponemal and non-treponemal positive	515	11	2	2
Liberia[108]	2013	Low	Sub-National	9 counties	B3	B1	Convenience	Lifetime	Treponemal and non-treponemal positive	155	0	0	0
Madagascar[109]	2012	Low	City	Antananariv-o	B2	B1	RDS	6 months	Treponemal and non-treponemal positive	211	16	6	8

Country and reference	Study year	Income level	Geographic coverage	Location (if not national)	Literature grade	Method grade	Recruitment method	Recency of injecting drug use	Syphilis diagnostic method	No. syphilis tested (N)	No. syphilis positive (n)	Crude prev. est. (%)	Adj. prev. est. (%)
Madagascar[109]	2012	Low	City	Toamasina	B2	B1	RDS	6 months	Treponemal and non-treponemal positive	193	9	5	5
Madagascar[109]	2012	Low	City	Antsiranana	B2	B1	RDS	6 months	Treponemal and non-treponemal positive	176	4	2	2
Mauritius[110]	2017	Upper middle	National		B3	A	RDS	Unspecified	Treponemal and non-treponemal positive	500	40	8	8
Mauritius[111]	2017	Upper middle	National		B3	A	RDS	3 months	Treponemal and non-treponemal positive	495	27	6	6
Mauritius[112]	2011	Upper middle	National		B3	B1	RDS	3 months	Treponemal and non-treponemal positive	499	23	6	5
Mauritius[113]	2009	Upper middle	Sub-National	Port Louis, Curepipe	A1	B1	Unspecified	3 months	Treponemal and non-treponemal positive	510	10	3	2
Nigeria[114]	2010	Lower middle	City	Lagos	A1	C	RDS	12 months	Treponemal and non-treponemal positive	328	2	1	1
Seychelles[115]	2017	High	Sub-National	Mahé, Praslin, La Digue	B3	A	Unspecified	6 months	Treponemal and non-treponemal positive	143	0	0	0
Seychelles[116]	2011	High	National		B2	B1	RDS	6 months	Treponemal and non-treponemal positive	345	1	1	0
Tanzania[117]	2019	Lower middle	City	Unguja	B2	A	RDS	3 months	Treponemal and non-treponemal positive	419	1	0	0
Tanzania[117]	2018	Lower middle	City	Pemba	B2	A	Convenience	3 months	Treponemal and non-treponemal positive	57	0	0	0
Tanzania[118]	2017	Lower middle	City	Dar Es Salaam	B2	B1	RDS	3 months	Treponemal and non-treponemal positive	611	18	0	0
Western Europe													
Albania[119]	2011	Upper middle	City	Tirana	C	B1	RDS	Unspecified	Rapid test	200	2	1	1
Germany[120]	2022	High	National		B2	A	Convenience	12 months	Treponemal and non-treponemal positive	584	0	0	0
Macedonia (TFYR)[121]	2017	Upper middle	City	Skopje	B3	B1	Unspecified	1 month	Treponemal positive without non-treponemal confirmation	288	2	1	0
Serbia[122]	2015	Upper middle	City	Kragujevac	A1	C	Convenience	Unspecified	Treponemal and non-treponemal positive	99	1	1	1
Serbia[123]	2014	Upper middle	Sub-National	Prishtina, Kosovo	B3	B1	RDS	1 month	Treponemal positive without non-treponemal confirmation	299	6	2	1
Serbia[123]	2014	Upper middle	Sub-National	Prizren, Kosovo	B3	B1	RDS	1 month	Treponemal positive without non-treponemal confirmation	199	1	1	0
Serbia[124]	2012	Upper middle	Sub-National	Belgrade and Novi Sad	B2	B1	RDS	1 month	Unknown	450	7	2	1

Notes. RDS = respondent-driven sampling. Where studies aggregated data over multiple years, the most recent year is reported as the study year; where studies reported disaggregated data over multiple years, prevalence for each year was extracted separately. Literature grade key: A1 = peer-reviewed journal article; A2 = abstract of published article only; B1 = published book/report/monograph from scholarly or commercial publisher; B2 = published book/report/monograph from international governmental or monitoring organisation; B3 = published book/report/monograph from other source; C = conference abstract; D = other unpublished report. Method grade key: A = multisite study with >1 sample types (e.g. needle-syringe programmes, drug treatment centres), B1 = single sample type and multiple sites; B2 = multiple sample types and a single site; C = single sample type and single site. Prevalence studies that used self-report data were not included in meta-analysis; these data are presented in Appendix K. Information on correction of syphilis are provided in Appendix D. Information on gender/sex-specific prevalence estimates are reported in Appendix I.

Appendix G. Country-level syphilis prevalence estimates, grouped by UNAIDS region.

	Crude prevalence	Heterogeneity (I^2)	Adjusted prevalence	Heterogeneity (I^2)	Data source
**Australasia**					
Australia	2.34 (0.49-6.70)	-	1.52 (0.19, 5.53)	-	[125]
New Zealand					
**Caribbean**					
Bahamas					
Bermuda					
Puerto Rico					
Dominican Republic					
Haiti					
Jamaica					
**Central Asia**					
Kazakhstan					
Kyrgyzstan	8.41 (6.68-10.41)	-	4.43 (3.18-5.98)	-	[3]
Tajikistan	2.18 (1.62-2.81)	-	1.87 (1.36-2.46)		[4,5]
Turkmenistan					
Uzbekistan					
**East and Southeast Asia**					
Brunei Darussalam					
Cambodia	3.81 (5.16-6.7)	-	3.55 (2.43-4.86)	-	[6-8]
China	6.87 (5.57-8.29)	88.9	4.97 (4.1-5.92)	80.0	[9-24]
Hong Kong (China)					
Indonesia	1.05 (0.22-2.33)	76.9	0.78 (0.11-1.87)	74.2	[25,26]
Japan					
Lao PDR					
Malaysia					
Mongolia					
Myanmar	1.12 (0.65-1.7)	84.1	0.70 (0.4-1.07)	72.1	[27-33]
Philippines	1.73 (0.12-4.87)	93.6	1.32 (0.09-3.67)	91.3	[34,35]
Republic of Korea					
Singapore					
Taiwan	19.54 (11.82-29.43)	-	14.94 (8.2-24.2)	-	[36]
Thailand	2.31 (0.18-6.35)	-	1.78 (0.19-4.62)	-	[37]
Timor Leste					
Viet Nam					
**Eastern Europe**					
Armenia	0.71 (0.06-1.83)	72.5	0.56 (0.03-1.53)	68.9	[38-41]
Azerbaijan	6.34 (4.88-7.97)	53.5	4.11 (3.07-5.29)	38.7	[42,43]
Belarus	0.19 (0-0.88)	68.0	0.15 (0-0.66)	53.0	[44]
Bosnia & Herzegovina	0.27 (0-0.79)	0.0	0.27 (0-0.79)	0.0	[45]
Bulgaria					
Czech Republic	0 (0-6.27)	-	1.3 (0.42-3.01)	-	[46]
Estonia	8.43 (6.58-12.99)	-	4.86 (2.86-7.66)	-	[47]
Georgia	5.89 (4.56-7.38)	-	3.13 (2.15-4.27)	-	[48]
Hungary	3.76 (1.53-7.6)	-	3.76 (1.53-7.6)	-	[49]
Latvia	4.42 (2.64-6.9)	-	2.46 (1.18-4.47)	-	[50]

	Crude prevalence	Heterogeneity (I^2)	Adjusted prevalence	Heterogeneity (I^2)	Data source
Lithuania	7.00 (4.70-9.96)	-	3.75 (2.11-6.11)	-	[50]
Moldova	3.69 (1.93-5.95)	85.3	2.62 (1.37-4.23)	78.6	[51,52]
Poland	0.52 (0.01-2.85)	-	0.52 (0.01-2.85)	-	[53]
Romania					
Russian Federation	11.40 (9.95-13.00)	-	6.55 (5.41-7.8)	-	[54,55]
Slovakia					
Ukraine	5.91 (4.35-7.67)	93.2	3.29 (2.46-4.23)	83.9	[56-58]
**Latin America**					
Argentina					
Bolivia					
Brazil					
Chile					
Colombia					
Costa Rica					
Ecuador					
El Salvador					
Guatemala					
Guyana					
Honduras					
Mexico	7.47 (3.85-12.14)	-	7.47 (3.85-12.14)	-	[59-61]
Nicaragua	1.7 (0.03-4.88)	-	1.7 (0.03-4.88)	-	[62,63]
Panama					
Paraguay					
Peru					
Suriname					
Uruguay					
Venezuela					
**Middle East and North Africa**					
Algeria					
Bahrain					
Cyprus					
Egypt					
Iraq					
Israel					
Jordan					
Kuwait					
Lebanon					
Libyan Arab Jamahiriya					
Morocco					
Occ. Palestinian Terr.					
Oman					
Qatar					
Saudi Arabia					
South Sudan					
Sudan					

	Crude prevalence	Heterogeneity (I^2)	Adjusted prevalence	Heterogeneity (I^2)	Data source
Syrian Arab Rep.					
Tunisia					
Turkey					
United Arab Emirates					
Yemen					
**North America**					
Canada	1.84 (0.22-6.47)	-	1.84 (0.22-6.47)	-	[64]
United States	4.27 (2.22-6.88)	-	3.95 (1.98-6.47)	-	[60,65]
**Pacific Island States and Territories**					
American Samoa					
Fed. States of Micronesia					
Fiji					
French Polynesia					
Guam					
Kiribati					
Marshall Islands					
New Caledonia					
Northern Mariana Islands					
Palau					
Papua New Guinea					
Samoa					
Solomon Islands					
Tonga					
Vanuatu					
**South Asia**					
Afghanistan	4.18 (2.28-6.58)	88.7	3.3 (1.81-5.16)	84.9	[66-69]
Bangladesh	2.22 (1.59-2.93)	80.7	4.67 (3.38-6.16)	91.9	[70-73]
Bhutan					
India	6.47 (4.04-9.41)	96.0	6.47 (4.04-9.41)	96.0	[74-79]
Iran	0.45 (0.12-1.15)	-	0.23 (0.03-0.81)	-	[80]
Maldives	0 (0-0.70)	-	0 (0-0.70)	-	[81]
Nepal	1.64 (0.92-2.54)	84.3	1.5 (0.82-2.35)	84.1	[82-97]
Pakistan	6.53 (4.27-9.20)	-	6.53 (4.27-9.20)	-	[98]
Sri Lanka	0.2 (0-0.79)	-	0.48 (0.04-1.24)	-	[99,100]
**Sub Saharan Africa**					
Angola					
Benin					
Botswana					
Burkina Faso					
Burundi	11.54 (7.98-15.61)	0.0	11.54 (8.00-15.61)	0.0	[101]
Cameroon					
Cape Verde					
Central African Rep.					
Chad					
Comoros	0 (0-4.11)	-	0 (0-4.11)	-	[102]

	Crude prevalence	Heterogeneity (I^2)	Adjusted prevalence	Heterogeneity (I^2)	Data source
Congo (Kinshasa)					
Côte d'Ivoire	1.82 (0.74-3.72)	-	0 (0-6.27)	-	[103]
Democratic Republic of the Congo	1.06 (0.63-1.67)	-	1.06 (0.63-1.67)	-	[104]
Djibouti					
Equatorial Guinea					
Eritrea					
Ethiopia	5.06 (2.64-8.68)	-	5.06 (2.64-8.68)	-	[105]
Gabon					
Gambia					
Ghana					
Guinea					
Guinea-Bissau					
Kenya	1.49 (0.12-1.15)	-	1.49 (0.41-3.76)	-	[106]
Lesotho					
Liberia	1.34 (0.56-2.41)	-	1.34 (0.56-2.41)	-	[107,108]
Madagascar	4.67 (2.11-8.1)	-	4.67 (2.11-8.1)	-	[109-113]
Malawi					
Mali					
Mauritania					
Mauritius					
Mozambique					
Namibia					
Niger					
Nigeria	0.61 (0.07-2.19)	-	0.61 (0.07-2.19)	-	[114]
Rwanda					
Sao Tome & Principe					
Senegal					
Seychelles	0.14 (0-0.82)	-	0.14 (0-0.82)	-	[115,116]
Sierra Leone					
Somalia					
South Africa					
Swaziland					
Togo					
Uganda					
United Rep. of Tanzania	0.79 (0-3.55)	-	0.79 (0-3.55)	-	[117,118]
Zambia					
Zimbabwe					
**Western Europe**					
Albania	1 (0.12-3.57)	-	0.5 (0.01-2.75)	-	[119]
Andorra					
Austria					
Belgium					
Croatia					
Denmark					
England					
Finland					

	Crude prevalence	Heterogeneity (I^2)	Adjusted prevalence	Heterogeneity (I^2)	Data source
FYR of Macedonia	0.69 (0.08-2.49)	-	0.45 (0.01-1.92)	-	[121]
France					
Germany	0 (0-0.63)	-	0 (0-0.63)	-	[120]
Greece					
Greenland					
Iceland					
Ireland					
Italy					
Liechtenstein					
Luxembourg					
Malta					
Monaco					
Montenegro					
Netherlands					
Northern Ireland					
Norway					
Portugal					
San Marino					
Scotland					
Serbia	1.32 (0.67-2.16)	0.0	0.88 (0.35-1.61)	0.0	[122-124]
Slovenia					
Spain					
Sweden					
Switzerland					
Wales					

Appendix H. Syphilis prevalence estimates stratified by sex/gender.

Table H.1. Syphilis prevalence estimates located among people who inject drugs and identify as man/male.

Country and reference	Study year	Income level	Geographic coverage	Location (if not national)	Literature grade	Method grade	Recruitment method	Recency of injecting drug use	Syphilis diagnostic method	No. syphilis tested (N)	No. syphilis positive (n)	Crude prev. est. (%)	Adj. prev. est. (%)
East and Southeast Asia													
Cambodia[8]	2017	Lower middle	National		B3	B1	RDS	12 months	Rapid test	310	16	5.2	3.6
China[20]	2008	Upper middle	City	Nanning	A1	C	Snowballing	Unspecified	Treponemal and non-treponemal positive	200	20	10	10
China[20]	2007	Upper middle	City	Nanning	A1	C	Snowballing	Unspecified	Treponemal and non-treponemal positive	200	10	5	5
Myanmar[28]	2014	Lower middle	City	Yangon	B2	A	Convenience	Unspecified	Non-treponemal positive without treponemal confirmation	200	21	10.5	5.6
Myanmar[28]	2014	Lower middle	City	Mandalay	B2	A	Convenience	Unspecified	Non-treponemal positive without treponemal confirmation	200	4	2	1.1
Myanmar[28]	2014	Lower middle	City	Lashio	B2	A	Convenience	Unspecified	Non-treponemal positive without treponemal confirmation	200	0	0	0
Myanmar[28]	2014	Lower middle	City	Tachileik	B2	A	Convenience	Unspecified	Non-treponemal positive without treponemal confirmation	87	3	3.5	1.8
Myanmar[28]	2014	Lower middle	City	Muse	B2	A	Convenience	Unspecified	Non-treponemal positive without treponemal confirmation	100	0	0	0
Myanmar[28]	2014	Lower middle	City	Myitkyina	B2	A	Convenience	Unspecified	Non-treponemal positive without treponemal confirmation	200	0	0	0
Myanmar[30]	2011	Lower middle	City	Yangon	B3	B1	Convenience	Unspecified	Non-treponemal positive without treponemal confirmation	200	7	3.5	1.9
Myanmar[30]	2011	Lower middle	City	Mandalay	B3	B1	Convenience	Unspecified	Non-treponemal positive without treponemal confirmation	200	1	0.5	0.3
Myanmar[30]	2011	Lower middle	City	Taunggyi	B3	B1	Convenience	Unspecified	Non-treponemal positive without treponemal confirmation	100	4	4	2.1
Myanmar[30]	2011	Lower middle	City	Lashio	B3	B1	Convenience	Unspecified	Non-treponemal positive without treponemal confirmation	200	0	0	0
Myanmar[30]	2011	Lower middle	City	Muse	B3	B1	Convenience	Unspecified	Non-treponemal positive without treponemal confirmation	200	0	0	0

Country and reference	Study year	Income level	Geographic coverage	Location (if not national)	Literature grade	Method grade	Recruitment method	Recency of injecting drug use	Syphilis diagnostic method	No. syphilis tested (N)	No. syphilis positive (n)	Crude prev. est. (%)	Adj. prev. est. (%)
Myanmar[30]	2011	Lower middle	City	Myitkyeena	B3	B1	Convenience	Unspecified	Non-treponemal positive without treponemal confirmation	200	0	0	0
Philippines[34]	2013	Lower middle	National		B2	B1	RDS	Unspecified	Unknown	767	37	4.82	3.6
Eastern Europe													
Armenia[39]	2018	Upper middle	City	Gyumri	B3	B1	Unspecified	1 month	Unknown	150	0	0	0
Armenia[39]	2018	Upper middle	City	Vanadzor	B3	B1	Unspecified	1 month	Unknown	150	0	0	0
Armenia[40]	2012	Upper middle	City	Gyumri	B2	B1	RDS	3 months	Treponemal and non-treponemal positive	45	0	0	0
Armenia[40]	2012	Upper middle	City	Vanadzor	B2	B1	RDS	3 months	Treponemal and non-treponemal positive	50	0	0	0
Armenia[41]	2011	Upper middle	City	Yerevan	B3	B1	RDS	3 months	Treponemal and non-treponemal positive	263	7	2.7	2.7
Azerbaijan[43]	2008	Upper middle	City	Sumgait	B3	B1	Convenience	1 month	Treponemal positive without non-treponemal confirmation	150	8	5.3	2.8
Azerbaijan[43]	2008	Upper middle	City	Masalli	B3	B1	Convenience	1 month	Treponemal positive without non-treponemal confirmation	150	16	10.7	5.7
Georgia[48]	2009	Upper middle	City	Telavi	B3	A	Unspecified	1 month	Treponemal positive without non-treponemal confirmation	205	11	5.4	2.8
Ukraine[57]	2015	Lower middle	National		B2	B1	RDS	1 month	Unknown	7424	245	3.3	2.5
Ukraine[58]	2009	Lower middle	Sub-National	17 oblasts	B2	B1	Unspecified	1 month	Treponemal positive without non-treponemal confirmation	3036	39	1.3	0.7
Latin America													
Mexico[59]	2007	Upper middle	City	Tijuana	A1	B1	RDS	6 months	Treponemal and non-treponemal positive	898	51	5.7	5.7
South Asia													
Afghanistan[67]	2009	Low	City	Kabul	A1	A	RDS	1 month	Treponemal and non-treponemal positive	483	6	1.2	1.2
Afghanistan[68]	2009	Low	City	Herat	A1	B1	RDS	1 month	Rapid test	160	3	2	1.3
Afghanistan[68]	2009	Low	City	Kabul	A1	B1	RDS	1 month	Rapid test	286	10	3.5	2.4
Afghanistan[68]	2009	Low	City	Mazar-i-Sharif	A1	B1	RDS	1 month	Rapid test	102	17	16.7	11.7
Afghanistan[69]	2008	Low	City	Herat	A1	B1	Convenience	6 months	Treponemal and non-treponemal positive	333	4	1.2	1.2
Afghanistan[69]	2008	Low	City	Jalalabad	A1	B1	Convenience	6 months	Treponemal and non-treponemal positive	96	0	0	0
Afghanistan[69]	2008	Low	City	Kabul	A1	B1	Convenience	6 months	Treponemal and non-treponemal positive	463	10	2.16	2.2

Country and reference	Study year	Income level	Geographic coverage	Location (if not national)	Literature grade	Method grade	Recruitment method	Recency of injecting drug use	Syphilis diagnostic method	No. syphilis tested (N)	No. syphilis positive (n)	Crude prev. est. (%)	Adj. prev. est. (%)
Afghanistan[69]	2008	Low	City	Mazar-i-Sharif	A1	B1	Convenience	6 months	Treponemal and non-treponemal positive	187	26	13.9	13.9
Bangladesh[70]	2020	Lower middle	Sub-National	Mymensingh	B2	A	Probability	1 month	Treponemal and non-treponemal positive	376	1	0.3	0.3
Bangladesh[71]	2011	Lower middle	City	Dhaka	B3	A	Convenience	12 months	Treponemal and non-treponemal positive with RPR reagin >1:8 titre	1243	53	4.3	10.7
Bangladesh[71]	2011	Lower middle	City	Mymensingh	B3	A	Convenience	12 months	Treponemal and non-treponemal positive with RPR reagin >1:8 titre	375	8	2.1	5.3
Bangladesh[71]	2011	Lower middle	City	Narayanganj	B3	A	Convenience	12 months	Treponemal and non-treponemal positive with RPR reagin >1:8 titre	261	14	5.4	13.4
Bangladesh[71]	2011	Lower middle	City	Tongi	B3	A	Convenience	12 months	Treponemal and non-treponemal positive with RPR reagin >1:8 titre	149	5	3.4	8.4
Bangladesh[71]	2011	Lower middle	City	Norsingdi	B3	A	Convenience	12 months	Treponemal and non-treponemal positive with RPR reagin >1:8 titre	101	8	7.9	19.8
Bangladesh[71]	2011	Lower middle	City	Chandpur	B3	A	Convenience	12 months	Treponemal and non-treponemal positive with RPR reagin >1:8 titre	115	7	6.1	15.2
Bangladesh[71]	2011	Lower middle	City	Teknaf	B3	A	Convenience	12 months	Treponemal and non-treponemal positive with RPR reagin >1:8 titre	96	5	5.2	13
Bangladesh[71]	2011	Lower middle	City	Rajshahi	B3	A	Convenience	12 months	Treponemal and non-treponemal positive with RPR reagin >1:8 titre	401	19	2.5	11.8
Bangladesh[71]	2011	Lower middle	City	Chapai Nawabganj	B3	A	Convenience	12 months	Treponemal and non-treponemal positive with RPR reagin >1:8 titre	220	4	1.8	4.5
Bangladesh[71]	2011	Lower middle	City	Kanshat	B3	A	Convenience	12 months	Treponemal and non-treponemal positive with RPR reagin >1:8 titre	92	1	1.1	2.7
Bangladesh[71]	2011	Lower middle	City	Char Norendrapur	B3	A	Convenience	12 months	Treponemal and non-treponemal positive with RPR reagin >1:8 titre	124	1	0.8	2
Bangladesh[71]	2011	Lower middle	City	Rangpur	B3	A	Convenience	12 months	Treponemal and non-treponemal positive with RPR reagin >1:8 titre	103	3	2.9	7.3
Bangladesh[71]	2011	Lower middle	City	Naogaon	B3	A	Convenience	12 months	Treponemal and non-treponemal positive with RPR reagin >1:8 titre	382	3	0.8	2

Country and reference	Study year	Income level	Geographic coverage	Location (if not national)	Literature grade	Method grade	Recruitment method	Recency of injecting drug use	Syphilis diagnostic method	No. syphilis tested (N)	No. syphilis positive (n)	Crude prev. est. (%)	Adj. prev. est. (%)
Bangladesh[71]	2011	Lower middle	City	Pabna	B3	A	Convenience	12 months	Treponemal and non-treponemal positive with RPR reagin >1:8 titre	101	0	0	0
Bangladesh[71]	2011	Lower middle	City	Ishwardi	B3	A	Convenience	12 months	Treponemal and non-treponemal positive with RPR reagin >1:8 titre	57	3	5.3	13.2
Bangladesh[71]	2011	Lower middle	City	Sirajganj	B3	A	Convenience	12 months	Treponemal and non-treponemal positive with RPR reagin >1:8 titre	344	6	1.7	4.4
Bangladesh[71]	2011	Lower middle	City	Hili	B3	A	Convenience	12 months	Treponemal and non-treponemal positive with RPR reagin >1:8 titre	138	3	2.2	5.4
Bangladesh[71]	2011	Lower middle	City	Dinajpur	B3	A	Convenience	12 months	Treponemal and non-treponemal positive with RPR reagin >1:8 titre	385	2	0.5	1.3
Bangladesh[71]	2011	Lower middle	City	Jessore	B3	A	Convenience	12 months	Treponemal and non-treponemal positive with RPR reagin >1:8 titre	190	5	2.6	6.6
Bangladesh[71]	2011	Lower middle	City	Sathkhira	B3	A	Convenience	12 months	Treponemal and non-treponemal positive with RPR reagin >1:8 titre	285	0	0	0
Bangladesh[71]	2011	Lower middle	City	Srimongol	B3	A	Convenience	12 months	Treponemal and non-treponemal positive with RPR reagin >1:8 titre	79	2	2.5	6.3
Bangladesh[71]	2011	Lower middle	City	Barisal	B3	A	Convenience	12 months	Treponemal and non-treponemal positive with RPR reagin >1:8 titre	404	3	0.7	1.9
Bangladesh[71]	2011	Lower middle	City	Benapole	B3	A	Convenience	12 months	Treponemal and non-treponemal positive with RPR reagin >1:8 titre	96	0	0	0
Bangladesh [72]	2006	Lower middle	City	Dhaka	A1	A	Convenience	12 months	RPR plus any Treponemal test (regardless of RPR titer)	4095	79	1.9	4.8
India[74]	2012	Lower middle	Sub-National	West Bengal	A1	C	Convenience		VDRL plus any Treponemal test (regardless of VDRL titer)	58	0	0	0
India[75]	2010	Lower middle	Sub-National	Punjab	A1	B1	Convenience	3 months	RPR plus any Treponemal test (regardless of RPR titer)	1155	20	2	1.7
India[76]	2010	Lower middle	Sub-National	Mumbai-Thane	B2	B1	RDS	6 months	RPR plus any Treponemal test (regardless of RPR titer)	327	27	8.3	8.3
India[76]	2010	Lower middle	Sub-National	Bishnupur	B2	B1	RDS	6 months	RPR plus any Treponemal test (regardless of RPR titer)	410	17	4.1	4.1
India[76]	2010	Lower middle	Sub-National	Churachandpur	B2	B1	RDS	6 months	RPR plus any Treponemal test (regardless of RPR titer)	411	11	2.7	2.7
India[76]	2010	Lower middle	Sub-National	Phek	B2	B1	RDS	6 months	RPR plus any Treponemal test (regardless of RPR titer)	418	58	13.9	13.9

Country and reference	Study year	Income level	Geographic coverage	Location (if not national)	Literature grade	Method grade	Recruitment method	Recency of injecting drug use	Syphilis diagnostic method	No. syphilis tested (N)	No. syphilis positive (n)	Crude prev. est. (%)	Adj. prev. est. (%)
India[76]	2010	Lower middle	Sub-National	Wokha	B2	B1	RDS	6 months	RPR plus any Treponemal test (regardless of RPR titer)	411	68	16.6	16.5
India[77]	2009	Lower middle	Sub-National	Manipur	A1	B1	RDS	6 months	Treponemal and non-treponemal positive	821	32	3.9	3.9
India[77]	2009	Lower middle	Sub-National	Nagaland	A1	B1	RDS	6 months	Treponemal and non-treponemal positive	829	111	13.4	13.4
India[78]	2008	Lower middle	City	Bishnupur	A1	B1	RDS	6 months	Treponemal and non-treponemal positive	420	24	5.7	5.7
India[78]	2008	Lower middle	City	Churachandpur	A1	B1	RDS	6 months	Treponemal and non-treponemal positive	419	4	0.9	1
India[78]	2008	Lower middle	City	Phek	A1	B1	RDS	6 months	Treponemal and non-treponemal positive	440	33	7.4	7.5
India[78]	2008	Lower middle	City	Wokha	A1	B1	RDS	6 months	Treponemal and non-treponemal positive	420	82	19.5	19.5
India[78]	2008	Lower middle	City	Mumbai/Thane	A1	B1	RDS	6 months	Treponemal and non-treponemal positive	355	17	4.9	4.8
India[79]	2006	Lower middle	Sub-National	Manipur	A1	B1	RDS	6 months	Treponemal and non-treponemal positive	839	34	4	4.1
India[79]	2006	Lower middle	Sub-National	Nagaland	A1	B1	RDS	6 months	Treponemal and non-treponemal positive	821	99	12	12.1
Iran[80]	2007	Lower middle	City	Tehran	A1	A	Convenience		Non-treponemal positive without treponemal confirmation	849	3	0.4	0.2
Maldives[81]	2008	Upper middle	City	Male'	B2	B1	Snowballing	6 months	Unknown	143	0	0	0
Maldives[81]	2008	Upper middle	City	Addu	B2	B1	Snowballing	6 months	Unknown	124	0	0	0
Nepal[82]	2020	Lower middle	Subnational	Province 1	B2	A	RDS	6 months	Unknown	200	0	0	0
Nepal[82]	2020	Lower middle	Subnational	Province 2	B2	A	RDS	6 months	Unknown	200	0	0	0
Nepal[82]	2020	Lower middle	Subnational	Bagmati	B2	A	RDS	6 months	Unknown	470	8	2	0
Nepal[82]	2020	Lower middle	Subnational	Gandaki	B2	A	RDS	6 months	Unknown	350	8	2	1
Nepal[82]	2020	Lower middle	Subnational	Lumbini	B2	A	RDS	6 months	Unknown	250	1	0	0
Nepal[82]	2020	Lower middle	Subnational	Karnali	B2	A	RDS	6 months	Unknown	100	0	0	0
Nepal[82]	2020	Lower middle	Subnational	Sudurpaschim	B2	A	RDS	6 months	Unknown	120	0	0	0
Nepal[83]	2020	Lower middle	National		B3	B2	RDS	6 months	Treponemal and non-treponemal positive	1690	20	1.2	1.2

Country and reference	Study year	Income level	Geographic coverage	Location (if not national)	Literature grade	Method grade	Recruitment method	Recency of injecting drug use	Syphilis diagnostic method	No. syphilis tested (N)	No. syphilis positive (n)	Crude prev. est. (%)	Adj. prev. est. (%)
Nepal[84]	2017	Lower middle	Sub-National	Kathmandu, Bhaktapur and Lalitpur districts	B3	A	RDS	3 months	Treponemal and non-treponemal positive with RPR reagin >1:8 titre	340	3	0.9	2.2
Nepal[86]	2017	Lower middle	Sub-National	West to Far West Terai Districts	B3	B2	Probability	3 months	Treponemal and non-treponemal positive with RPR reagin >1:8 titre	300	6	2	5
Nepal[89]	2015	Lower middle	Sub-National	Eastern Terai	B3	B1	Probability	3 months	Treponemal and non-treponemal positive with RPR reagin >1:8 titre	360	4	1.1	2.8
Nepal[90]	2015	Lower middle	Sub-National	Kathmandu Valley	B3	B1	RDS	3 months	Treponemal and non-treponemal positive with RPR reagin >1:8 titre	340	0	0	0
Nepal[91]	2015	Lower middle	Sub-National	Pokhara Valley	B3	B1	RDS	3 months	Treponemal and non-treponemal positive with RPR reagin >1:8 titre	345	4	1.1	2.9
Nepal[92]	2012	Lower middle	Sub-National	Eastern Terai	B3	B1	Probability	3 months	Treponemal and non-treponemal positive	100	6	1.7	6
Nepal[93]	2011	Lower middle	Sub-National	Kathmandu Valley	B3	B1	RDS	3 months	Treponemal and non-treponemal positive with RPR reagin >1:8 titre	340	0	0	0
Nepal[94]	2009	Lower middle	Sub-National	Eastern Terai	B3	B1	Probability	3 months	Treponemal and non-treponemal positive	345	6	1.7	1.7
Nepal[95]	2009	Lower middle	Sub-National	Kathmandu Valley	B3	B1	RDS	3 months	Treponemal and non-treponemal positive	300	5	1.5	1.7
Nepal[96]	2009	Lower middle	Sub-National	Pokhara Valley	B3	B1	RDS	3 months	Treponemal and non-treponemal positive	300	2	0.5	0.7
Nepal[97]	2009	Lower middle	Sub-National	Western to Far-Western Terai	B3	B1	Probability	3 months	Treponemal and non-treponemal positive	300	5	1.7	1.7
Sub-Saharan Africa													
Democratic Republic of the Congo[104]	2019	Low	National		B3	A	RDS	12 months	Treponemal and non-treponemal positive	1603	14	0.9	0.9
Liberia[108]	2013	Low	Sub-National	9 Counties	B3	B1	Convenience	Lifetime	Treponemal and non-treponemal positive	155	0	0	0
Madagascar[109]	2012	Low	City	Antananarivo	B2	B1	RDS	6 months	Treponemal and non-treponemal positive	132	8	6.1	6.1
Madagascar[109]	2012	Low	City	Toamasina	B2	B1	RDS	6 months	Treponemal and non-treponemal positive	163	5	3.1	3.1
Madagascar[109]	2012	Low	City	Antsiranana	B2	B1	RDS	6 months	Treponemal and non-treponemal positive	167	4	2.4	2.4
Mauritius[110]	2017	Upper middle	National		B3	A	RDS	Unspecified	Treponemal and non-treponemal positive	431	25	5.9	5.8

Country and reference	Study year	Income level	Geographic coverage	Location (if not national)	Literature grade	Method grade	Recruitment method	Recency of injecting drug use	Syphilis diagnostic method	No. syphilis tested (N)	No. syphilis positive (n)	Crude prev. est. (%)	Adj. prev. est. (%)
Nigeria[114]	2010	Lower middle	City	Lagos	A1	C	RDS	12 months	Treponemal and non-treponemal positive	328	2	0.6	0.6
Tanzania[117]	2018	Lower middle	City	Pemba	B2	A	Convenience	3 months	Treponemal and non-treponemal positive	57	0	0	0

Notes. RDS = respondent-driven sampling. Where studies aggregated data over multiple years, the most recent year is reported as the study year; where studies reported disaggregated data over multiple years, prevalence for each year was extracted separately. Literature grade key: A1 = peer-reviewed journal article; A2 = abstract of published article only; B1 = published book/report/monograph from scholarly or commercial publisher; B2 = published book/report/monograph from international governmental or monitoring organisation; B3 = published book/report/monograph from other source; C = conference abstract; D = other unpublished report. Method grade key: A = multisite study with >1 sample types (e.g. needle-syringe programmes, drug treatment centres), B1 = single sample type and multiple sites; B2 = multiple sample types and a single site; C = single sample type and single site. Prevalence studies that used self-report data are presented in Appendix K.

Table H.2. Syphilis prevalence estimates located among people who inject drugs and identify as woman/female.

Country and reference	Study year	Income level	Geographic coverage	Location (if not national)	Literature grade	Method grade	Recruitment method	Recency of injecting drug use	Syphilis diagnostic method	No. syphilis tested (N)	No. syphilis positive (n)	Crude prev. est. (%)	Adj. prev. est. (%)
East and Southeast Asia													
Cambodia[8]	2017	Lower middle	Sub-National	Phnom Penh & 11 Provinces	B3	B1	RDS	12 months	Rapid test	83	8	9.6	6.7
China[20]	2008	Upper middle	City	Guangxi, Nanning city	A1	C	Snowballing	Unspecified	Treponemal and non-treponemal positive	50	6	12	12
China[20]	2007	Upper middle	City	Guangxi, Nanning city	A1	C	Snowballing	Unspecified	Treponemal and non-treponemal positive	33	3	9.1	9.1
Eastern Europe													
Armenia[40]	2012	Upper middle	City	Gyumri	B2	B1	RDS	3 months	Treponemal and non-treponemal positive	5	0	0	0
Armenia[41]	2011	Upper middle	City	Yerevan	B3	B1	RDS	3 months	Treponemal and non-treponemal positive	7	4	57.1	57.1
Ukraine[57]	2015	Lower middle	National		B2	B1	RDS	1 month	Unknown	1851	94	5.1	3.8
Ukraine[58]	2009	Lower middle	Sub-National	17 oblasts	B2	B1	Unspecified	1 month	Treponemal positive without non-treponemal confirmation	926	31	3.4	1.8
Latin America													
Mexico[59]	2007	Upper middle	City	Tijuana	A1	B1	RDS	6 months	Treponemal and non-treponemal positive	158	25	15.8	15.8
South Asia													
Bangladesh[72]	2006	Lower middle	City	Dhaka	A1	A	Convenience	12 months	Treponemal and non-treponemal positive with RPR reagin >1:8 titre	121	12	9.9	24.8
Iran[80]	2007	Lower middle	City	Tehran	A1	A	Convenience	Unspecified	Non-treponemal positive without treponemal confirmation	38	1	2.6	1.4
Maldives[81]	2008	Upper middle	City	Male'	B2	B1	Snowballing	6 months	Unknown	4	0	0	0
Maldives[81]	2008	Upper middle	City	Addu	B2	B1	Snowballing	6 months	Unknown	5	0	0	0
Nepal[82]	2020	Lower middle	Subnational	Province 1, Bagmati, Gandanki	B2	A	RDS	6 months	Unknown	150	15	10	5
Nepal[83]	2020	Lower middle	National		B3	B2	Convenience	6 months	Treponemal and non-treponemal positive	150	15	10	10

Nepal[85]	2017	Lower middle	Sub-National	Pokhara Valley	B3	B1	Convenience	3 months	Treponemal and non-treponemal positive	155	6	3.9	3.9
Nepal[87]	2016	Lower middle	City	Kathmandu	C	C	Probability	Unspecified	Non-treponemal positive without treponemal confirmation	160	13	8	4.3
Nepal[88]	2016	Lower middle	Sub-National	Kathmandu valley	B3	A	Probability	3 months	Rapid test	160	12	7.5	5.3
Sub-Saharan Africa													
Democratic Republic of the Congo[104]	2019	Low	National		B3	A	RDS	12 months	Treponemal and non-treponemal positive	98	2	2	2
Madagascar[109]	2012	Low	City	Antananarivo	B2	B1	RDS	6 months	Treponemal and non-treponemal positive	79	8	10.1	10.1
Madagascar[109]	2012	Low	City	Toamasina	B2	B1	RDS	6 months	Treponemal and non-treponemal positive	30	4	13.3	13.3
Madagascar[109]	2012	Low	City	Antsiranana	B2	B1	RDS	6 months	Treponemal and non-treponemal positive	9	0	0	0
Mauritius[110]	2017	Upper middle	National		B3	A	RDS	Unspecified	Treponemal and non-treponemal positive	62	11	17.4	17.7

Notes. RDS = respondent-driven sampling. Where studies aggregated data over multiple years, the most recent year is reported as the study year; where studies reported disaggregated data over multiple years, prevalence for each year was extracted separately. Literature grade key: A1 = peer-reviewed journal article; A2 = abstract of published article only; B1 = published book/report/monograph from scholarly or commercial publisher; B2 = published book/report/monograph from international governmental or monitoring organisation; B3 = published book/report/monograph from other source; C = conference abstract; D = other unpublished report. Method grade key: A = multisite study with >1 sample types (e.g. needle-syringe programmes, drug treatment centres), B1 = single sample type and multiple sites; B2 = multiple sample types and a single site; C = single sample type and single site. Prevalence studies that used self-report data are presented in Appendix K.

Appendix I. Sensitivity analyses of syphilis prevalence estimates.

Table I.1. Prevalence of syphilis among people who inject drugs, restricted to the most recent data for each country (i.e., data points within five years of the most recent data point).

	Number of countries with evidence of injecting drug use	Number of countries with syphilis prevalence data	Number of point prevalence data	Study sample size (range)	Number of people who inject drugs tested (column %)	Number of people who inject drugs with positive syphilis test	Uncorrected syphilis prevalence (95% CI)	Corrected syphilis prevalence (95% CI)
**Australasia**	2	1	1	128	128 (0.2)	3	2.3 (0.5-6.7)	1.6 (0.2-5.5)
**Caribbean**	8	-	-	-	-	-	-	-
**Central Asia**	5	2	2	904-1916	2820 (4.1)	89	4.7 (2.9-5.9)	2.5 (1.7-3.5)
**East and Southeast Asia**	17	7	41	55-2267	18120 (26.6)	572	4.4 (3.3-5.7)	4.0 (3.1-5.2)
**Eastern Europe**	17	14	48	100-6001	16757 (24.6)	573	9.2 (6.7-12.3)	6.9 (4.8-9.8)
**Latin America**	19	2	4	100-1056	1568 (2.3)	112	7.1 (3.6-11.8)	7.1 (3.6-11.8)
**Middle East & North Africa**	21	-	-	-	-	-	-	-
**North America**	2	2	2	71-109	180 (0.3)	5	0.1 (0-0.3)	0.1 (0-0.3)
**Pacific Island states & terr.**	15	-	-	-	-	-	-	-
**South Asia**	9	8	57	58-1690	20189 (29.6)	887	5.4 (3.2-8.1)	5.3 (3.2-8)
**Sub-Saharan Africa**	44	12	20	30-1701	6293 (9.2)	197	1.8 (0.8-4.4)	1.8 (0.8-4.4)
**Western Europe**	31	4	7	99-584	2119 (3.1)	19	0.3 (0.1-1.1)	0.2 (0.1-1)
**Global**	**190**	**51**	**181**	**30-6001**	**68174 (100)**	**2457**	**3.9 (2.6-5.7)**	**3.4 (2.2-5.0)**

Notes. CI = confidence interval. See Appendix D for information of syphilis prevalence correction.

Table I.2. Prevalence of syphilis among people who inject drugs, restricted to people who had recently injected drugs (i.e., within the past 12 months).

	Number of countries with evidence of injecting drug use	Number of countries with syphilis prevalence data	Number of point prevalence data	Study sample size (range)	Number of people who inject drugs tested (column %)	Number of people wo inject drugs with positive syphilis test	Uncorrected syphilis prevalence (95% CI)	Corrected syphilis prevalence (95% CI)
**Australasia**	2	-	-	-	-	-	-	-
**Caribbean**	8	-	-	-	-	-	-	-
**Central Asia**	5	2	3	904-1916	3308 (4.3)	166	5.4 (3.8-6.7)	3.2 (2.3-4.3)
**East and Southeast Asia**	17	6	35	55-2267	21845 (21.7)	842	5.4 (3.9-7.1)	5 (3.9-6.3)
**Eastern Europe**	17	8	63	100-6001	29472 (32.7)	1269	9.5 (7.9-11.2)	5.1 (4-6.4)
**Latin America**	19	1	3	100-1056	1468 (2.9)	111	5.9 (3.7-8.7)	5.2 (3.2-7.8)
**Middle East & North Africa**	21	-	-	-	-	-	-	-
**North America**	2	1	2	71-109	250 (0.3)	11	4.3 (2.1-7.3)	4.3 (2.1-7.3)
**Pacific Island states & terr.**	15	-	-	-	-	-	-	-
**South Asia**	9	7	88	58-1690	33126 (32.9)	1275	6.3 (4-9.1)	6.4 (4.1-9.2)
**Sub-Saharan Africa**	44	11	21	30-1701	7090 (4.9)	191	1.8 (0.7-4.4)	1.8 (0.7-4.4)
**Western Europe**	31	3	5	99-584	1820 (0.4)	16	0.3 (0.1-1.1)	0.2 (0.1-1)
**Global**	**190**	**39**	**220**	**30-6001**	**98379 (100)**	**3881**	**5.3 (3.6-7.5)**	**4.4 (2.9-6.4)**

Notes. CI = confidence interval. See Appendix D for information of syphilis prevalence correction.

Table I.3. Prevalence of syphilis among people who inject drugs, excluding samples that only reported syphilis prevalence for one gender/sex.

	Number of countries with evidence of injecting drug use	Number of countries	Number of point prevalence data	Study sample size (range)	Number of people who inject drugs tested (column %)	Number of people wo inject drugs with positive syphilis test	Uncorrected syphilis prevalence (95% CI)	Corrected syphilis prevalence (95% CI)
**Australasia**	2	1	1	128	128 (0.1)	3	2.3 (0.5-6.7)	1.6 (0.2-5.5)
**Caribbean**	8	-	-	-	-	-	-	-
**Central Asia**	5	2	3	488-1916	3308 (3.8)	166	5.4 (3.5-6.7)	3.2 (2.3-4.3)
**East and Southeast Asia**	17	7	65	55-2530	30214 (34.5)	842	6.3 (5-7.9)	4.6 (3.7-5.7)
**Eastern Europe**	17	14	69	50-9407	32304 (36.9)	1269	8.1 (6.8-9.8)	4.7 (3.7-6)
**Latin America**	19	2	5	41-1056	1609 (1.8)	111	7.2 (3.6-11.7)	7.2 (3.6-11.7)
**Middle East & North Africa**	21	-	-	-	-	-	-	-
**North America**	2	2	4	71-150	430 (0.5)	11	0.1 (0-0.3)	0.1 (0-0.3)
**Pacific Island states & terr.**	15	-	-	-	-	-	-	-
**South Asia**	9	6	20	102-4216	10230 (11.7)	1275	5.6 (3.3-8.3)	5.5 (3.3-8.2)
**Sub-Saharan Africa**	44	11	21	30-1701	7262 (8.3)	191	2.6 (1.4-4.3)	2.6 (1.4-4.3)
**Western Europe**	31	4	7	99-584	2119 (2.4)	16	0.3 (0.1-1.1)	0.2 (0.1-1)
**Global**	**190**	**48**	**194**	**30-4216**	**87595 (100)**	**3379**	**4.3 (3.2-5.9)**	**3.3 (2.3-4.6)**

Notes. CI = confidence interval. See Appendix D for information of syphilis prevalence correction.

Appendix J. Association between study-level and country-level characteristics and syphilis prevalence.

Figure J.1. Association between study-level demographic and behavioural profiles of people who inject drugs with syphilis prevalence.

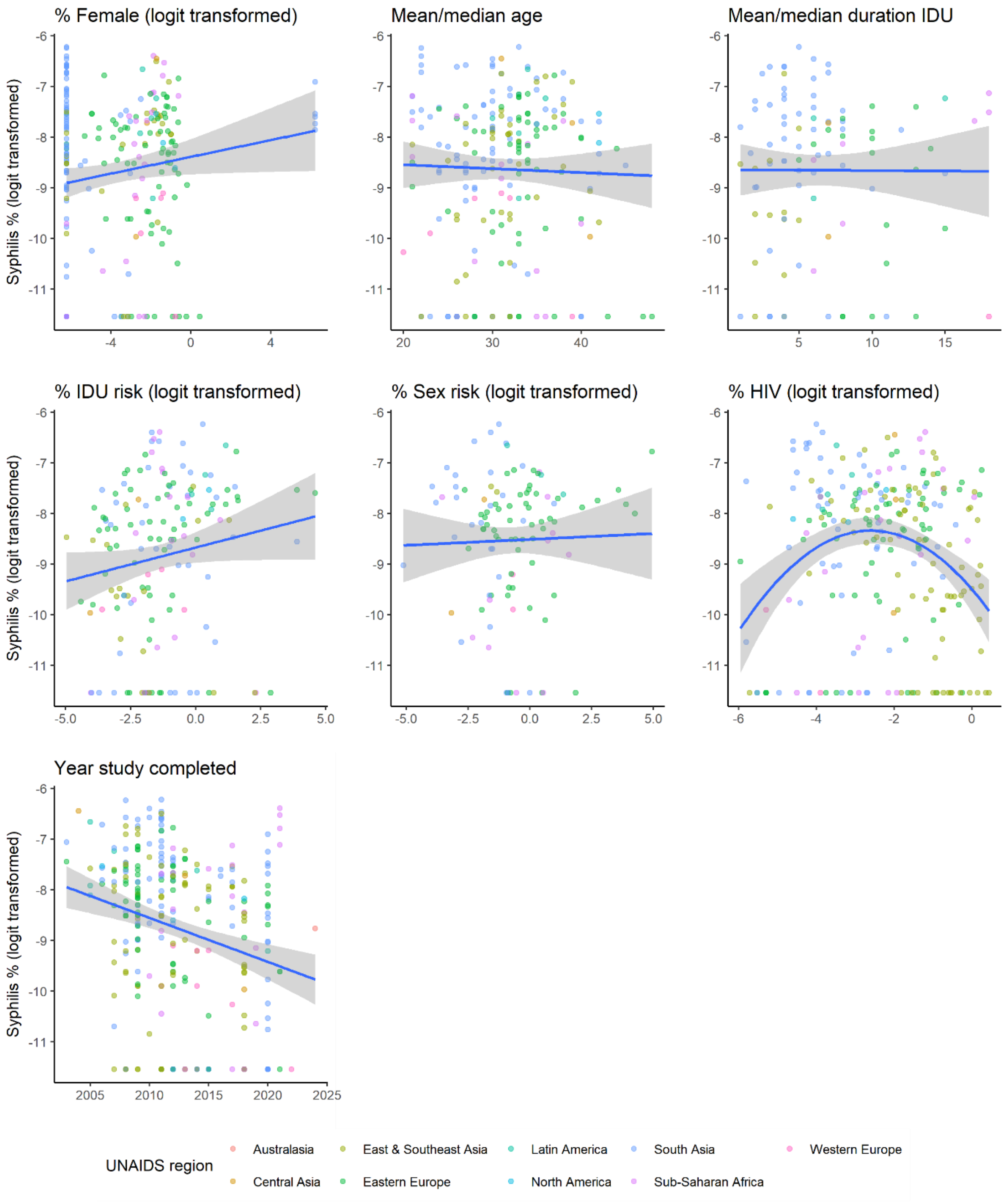

Figure J.2. Association between country-level demographic and behavioural profiles of people who inject drugs with syphilis prevalence.

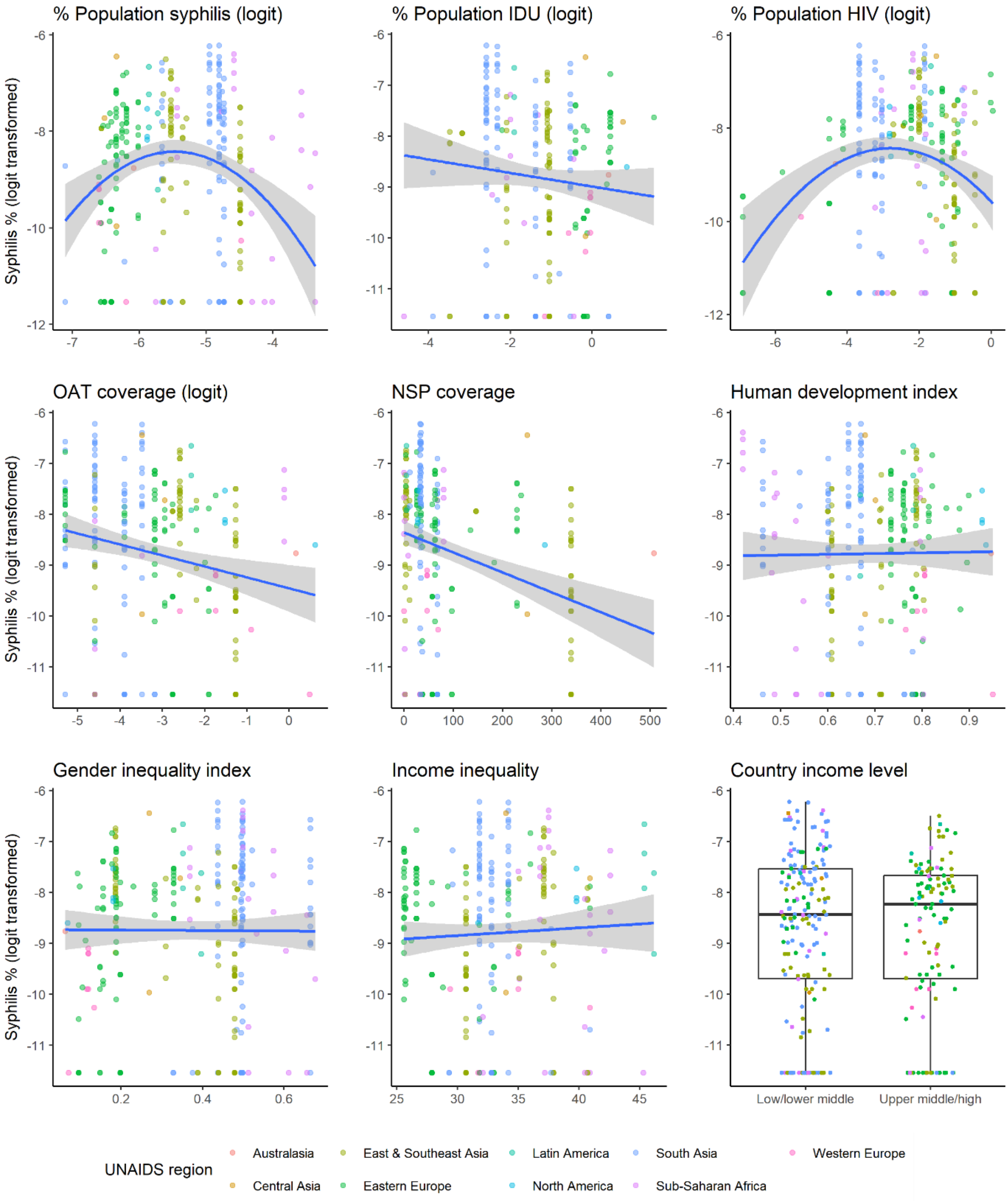

Appendix K. Studies reporting prevalence of sexually transmitted infections using participant self-report data.

Country and reference	Study year	Income level	Geographic coverage	Location (if not national)	Literature grade	Method grade	Recruitment method	Recency of injecting drug use	Timeframe for STI diagnosis	Sample size (N)	Prev. (%)	Gender-specific sample size (N)	Gender-specific prev. (%)
Any sexually transmitted infection													
Afghanistan[69]	2008	Low	Sub-National	Hirat, Jalalabad, Kabul, Mazar-i-Sharif	A1	B1	Convenience	6 months	6 months	1078	6	M: 1078	M: 6
Australia[126]	2024	High	National		B2	B1	Convenience	6 months	6 months	802	3		
Australia[127]	2019	High	National		B3	A	Convenience	1 month	12 months	855	3		
Azerbaijan[42]	2012	Upper middle	City		B2	B2	Unspecified	12 months	12 months	1200	9		
Benin[128]	2015	Lower middle	National		B2	B1	Convenience	12 months	12 months	383	14		
Bosnia and Herzegovina[45]	2012	Upper middle	National		B3	B1	RDS	1 month	Lifetime	977	8		
Burundi[129]	2017	Low	City	Bujumbura	A1	B1	Unspecified	3 months	Lifetime	127	22		
Cambodia[6]	2017	Lower middle	National		A1	A	RDS	12 months	12 months	310	28		
Canada[130]	2012	High	City	Prince George	B3	B1	Convenience	6 months	Lifetime	150	35		
Canada[131]	2011	High	Sub-National	Ontario	D	B1	Convenience	6 months	Lifetime	1323	36		
China[132]	2010	Upper middle	Sub-National	Yunnan	A1	A	Convenience	Lifetime	Lifetime	1699	16		
Croatia[133]	2007	High	Sub-National		A1	B1	Unspecified	Unspecified	Lifetime	76	30		
Democratic Republic of the Congo[134]	2014	Low	National		B3	C	Convenience	Unspecified	12 months	98	2		
Ethiopia[135]	2019	Low	City	Addis Ababa Ababa	A1	B1	RDS	3 months	12 months	276	24		
France[136]	2015	High	City	Paris	A1	C	Convenience	Lifetime	12 months	80	1		
Iran[137]	2016	Lower middle	City	Tehran	A1	C	Convenience	1 month	Lifetime	500	28	M: 500	M: 28
Iran[138]	2006	Lower middle	City	Tehran	A1	B1	Convenience	Unspecified	Lifetime	417	7	M: 417	M: 7
Kazakhstan[139]	2010	Upper middle	National		B2	B1	Snowballing	1 month	12 months	503	5		
Kenya[140]	2017	Lower middle	Sub-national	Kisumu, Nairobi, Mombasa, Kilifi	B3	A	Probability	Unspecified	3 months	1131	25		
Kyrgyzstan[139]	2010	Lower middle	National		B2	B1	Snowballing	1 month	12 months	520	7		
Lebanon[141]	2015	Lower middle	City	Beirut	B3	A	RDS	3 months	12 months	390	13		

Country and reference	Study year	Income level	Geographic coverage	Location (if not national)	Literature grade	Method grade	Recruitment method	Recency of injecting drug use	Timeframe for STI diagnosis	Sample size (N)	Prev. (%)	Gender-specific sample size (N)	Gender-specific prev. (%)
Malaysia[142]	2017	Upper middle	National		B3	A	RDS	1 month	12 months	1413	1	M: 1413	M: 1
Mauritius[112]	2011	Upper middle	National		B3	B1	RDS	3 months	12 months	500	4		
Mexico[143]	2023	Upper middle	City	El Paso, Cd. Juarez	A	A	RDS	Not specified	Lifetime	363	36		
Mozambique[144]	2014	Low	Sub-National	Maputo and Nampula/ Nacala	A1	B1	RDS	Lifetime	12 months	492	16		
Mozambique[145]	2014	Low	Sub-National	Maputo and Nampula/ Nacala	A1	C	RDS	Lifetime	12 months	492	13		
Mozambique[146]	2014	Low	Sub-National	Maputo and Nampula/ Nacala	B3	A	RDS	Lifetime	12 months	492	17		
Mozambique [147]	2014	Low	Sub-National	Maputo and Nampula/ Nacala	A1	A	RDS	Lifetime	12 months	492	24		
Myanmar[27]	2018	Lower middle	National		B3	A	RDS	1 month	12 months	6061	3		
Nepal[148]	2011	Lower middle	National		B3	B1	Convenience	12 months	6 months	596	14		
Nepal[94]	2009	Lower middle	Sub-National	Eastern Terai	B3	B1	Probability	3 months	12 months	345	13		
Nepal[95]	2009	Lower middle	Sub-National	Kathmandu Valley	B3	B1	RDS	3 months	12 months	300	7		
Nepal[96]	2009	Lower middle	Sub-National	Pokhara Valley	B3	B1	RDS	3 months	12 months	300	12		
Nepal[97]	2009	Lower middle	Sub-National	Western to Far-Western Terai	B3	B1	Probability	3 months	12 months	300	19		
Nigeria[149]	2020	Lower middle	National		B2	A	Probability	12 months	12 months	4414	34		
Occupied Palestinian Territory[150]	2010	Upper middle	City	East Jerusalem Governorate	B2	B1	RDS	1 month	Lifetime	192	5		
Pakistan [151]	2014	Lower middle	Sub-National	Punjab	B3	B1	Probability	6 months	6 months	3840	14		
Pakistan[152]	2011	Lower middle	National		A1	B1	Probability	6 months	6 months	3544	3		
Pakistan[153]	2008	Lower middle	National		B2	B1	Probability	6 months	6 months	2979	8		
Pakistan[154]	2005	Lower middle	Sub-National	Rawalpindi	A1	C	Probability	6 months	6 months	200	5		

Country and reference	Study year	Income level	Geographic coverage	Location (if not national)	Literature grade	Method grade	Recruitment method	Recency of injecting drug use	Timeframe for STI diagnosis	Sample size (N)	Prev. (%)	Gender-specific sample size (N)	Gender-specific prev. (%)
Philippines[35]	2011	Lower middle	Sub-National	Cebu, Gen Santos, Zamboanga, Mandaue	B3	B1	RDS	6 months	12 months	1283	5		
Puerto Rico[155]	2014	High	National		B3	B1	Convenience	12 months	Month	17	29	F: 17	F: 29
Puerto Rico[156]	2009	High	City	San Juan	B3	B1	RDS	12 months	12 months	460	5	M: 400<br>F: 73	M: 3<br>F: 15
Puerto Rico[157]	2006	High	City	San Juan	B3	B1	RDS	12 months	12 months	507	14	M: 442<br>F: 67	M: 12<br>F: 27
Russia[158]	2004	Upper middle	City	Togliatti	A1	B1	RDS	1 month	Lifetime	96	9		
Russia[158]	2001	Upper middle	City	Togliatti	A1	B1	Convenience	1 month	Lifetime	133	2		
Seychelles[116]	2011	High	National		B2	B1	RDS	6 months	12 months	345	7		
Sierra Leone[159]	2013	Low	National		B2	B1	Convenience	Unspecified	12 months	210	25	M: 192<br>F: 18	M: 25<br>F: 22
Spain[160]	2019	High	City	Barcelona	A1	C	Convenience	6 months	12 months	98	6		
Spain[161]	2019	High	Sub-National	Catalonia	B3	B1	Unspecified	6 months	Lifetime	697	3	M: 565<br>F: 125	M: 2<br>F: 6
Spain[162]	2017	High	City	Barcelona	A1	C	Convenience	6 months	12 months	410	7		
Spain[163]	2015	High	Sub-National	Catalonia	A1	B1	Convenience	6 months	Lifetime	120	36	F: 120	F: 36
Spain[164]	2012	High	Sub-National	Catalonia	A1	B1	Convenience	6 months	Lifetime	2198	26		
Spain[165]	2011	High	Sub-National	Catalonia	A1	B2	Unspecified	6 months	Lifetime	754	15		
Sri Lanka[99]	2017	Lower middle	City	Colombo	B3	B1	RDS		12 months	305	1		
Sri Lanka[100]	2014	Lower middle	City	Colombo	B3	C	RDS	12 months	12 months	218	3		
Taiwan[166]	2013	High	Sub-National	Taipei	A1	C	Convenience	Lifetime	Lifetime	827	17		
Tajikistan[167]	2015	Lower middle	City	Kulob, Khudjand	A1	C	Convenience	1 month	Lifetime	199	13		
Tajikistan[139]	2010	Lower middle	National		B2	B1	Snowballing	1 month	12 months	431	17		
Tajikistan[5]	2004	Lower middle	City	Dushanbe	A1	B1	Convenience	1 month	Lifetime	488	21		
Togo[168]	2018	Low	National		A	A	Snowballing	6 months	Lifetime	384	17		
Tunisia[169]	2011	Lower middle	Sub-National	Tunis, Bizerte	B2	B1	RDS	Unspecified	12 months	802	21		
Uganda[129]	2017	Low	Sub-National	Kampala, Mbale	A1	B2	Unspecified	3 months	Lifetime	125	57		

Country and reference	Study year	Income level	Geographic coverage	Location (if not national)	Literature grade	Method grade	Recruitment method	Recency of injecting drug use	Timeframe for STI diagnosis	Sample size (N)	Prev. (%)	Gender-specific sample size (N)	Gender-specific prev. (%)
United States[170]	2019	High	Sub-National	Seattle and King County, Washington	A1	B2	Convenience	3 months	12 months	720	5		
United States[171]	2018	High	National		B3	B1	RDS	12 months	12 months	11437	6	M: 7891<br>F: 3449	M: 5<br>F: 8
United States[172]	2018	High	National		C	B1	RDS	12 months	12 months	3391	8	F: 3391	F: 8
United States[173]	2016	High	City	Seattle	C	B1	Unspecified	Unspecified	12 months	377	8	F: 377	F: 8
United States[174]	2015	High	National		A1	B2	Probability	Lifetime	Lifetime	323	11	M: 181<br>F: 142	M: 8<br>F: 15
United States[175]	2012	High	National		B3	B1	RDS	12 months	12 months	10140	4	M: 7183<br>F: 2858	M: 4<br>F: 6
United States[176]	2010	High	City	San Diego	A1	A	RDS	3 months	Lifetime	510	18		
United States[156]	2009	High	National		B3	B1	RDS	12 months	12 months	9700	7	M: 7540<br>F: 2745	M: 5<br>F: 11
United States[177]	2009	High	National		A1	B1	RDS	12 months	12 months	2305	13		
United States[178]	2009	High	City	New York City	A	A	RDS	6 months	Lifetime	125	70		
United States[179]	2006	High	City	Raleigh–Durham	A1	A	Convenience	1 month	Lifetime	822	48		
United States[157]	2006	High	National		B3	B1	RDS	12 months	12 months	10631	13	M: 7383<br>F: 3100	M: 12<br>F: 16
United States[180]	2000	High	City	Chicago	A1	B1	Convenience	6 months	Lifetime	889	47		
Any sexually transmitted infection-like symptoms													
Afghanistan[69]	2008	Low	Sub-National	Hirat, Jalalabad, Kabul, Mazar-i-Sharif	A1	B1	Convenience	6 months	6 months	1078	16	M: 1078	M: 16
Bangladesh[70]	2020	Lower middle	Sub-National	Narayanganj, Cumilla, Gazipur, Dhaka, Rajshahi, Chapainawabgani, Barishal, Mymensingh	B2	A	RDS	1 month	12 months	3033	5		
Bangladesh[181]	2016	Lower middle	National		B3	B1	Convenience	12 months	12 months	1354	31	M: 1281<br>F: 73	M: 29<br>F: 76
Bangladesh[182]	2007	Lower middle	Sub-National	Dhaka, Rajshahi, Chapainawabganj, Chandpur	B3	B1	Probability	Unspecified	12 months	1196	28		
Benin[183]	2017	Lower middle	Sub-National	Atlantique, Littoral, Ouémé, Zou, Mono, Borgou, Atacora	B2	A	Convenience	Unspecified	12 months	496	12		

Country and reference	Study year	Income level	Geographic coverage	Location (if not national)	Literature grade	Method grade	Recruitment method	Recency of injecting drug use	Timeframe for STI diagnosis	Sample size (N)	Prev. (%)	Gender-specific sample size (N)	Gender-specific prev. (%)
Benin[184]	2013	Lower middle	National		B2	B1	Convenience	12 months	12 months	114	13		
Cambodia[8]	2017	Lower middle	National		B3	B1	RDS	12 months	3 months	310	28		
China[185]	2007	Upper middle	City	Yunnan, Kaiyuan	A1	A	Snowballing	6 months	6 months	314	17		
Comoros[102]	2020	Lower middle	City	Ngazidja, Ndzuwani, Mwali	B2	A	RDS	12 months	Lifetime	88	75		
India[186]	2015	Lower middle	National		B2	A	Probability	3 months	12 months	19902	16	M: 19902	M: 16
India[187]	2014	Lower middle	Sub-National	Manipur	A1	B2	RDS	3 months	3 months	1594	180	M: 1594	M: 180
India[75]	2010	Lower middle	Sub-National	Punjab	A1	B1	Convenience	3 months	12 months	1153	18	M: 1153	1M: 8
India[76]	2010	Lower middle	Sub-National	Mumbai-Thane, Bishnupur, Churachandpur, Phek	B2	B1	RDS	6 months	12 months	1566	7	M: 1566	M: 7
India[188]	2009	Lower middle	Sub-National	Northeast India	A1	B1	RDS	6 months	12 months	3392	18	M: 3392	M: 18
Indonesia[189]	2019	Upper middle	City	Badung	B2	B1	RDS	6 months	12 months	185	20		
Indonesia[190]	2015	Upper middle	City		A1	A	RDS	12 months	Month	731	44	F: 731	F: 44
Iran[191]	2017	Lower middle	National		A	A	Probability	Not specified	12 months	496	19		
Iran[138]	2006	Lower middle	City	Tehran	A1	B1	Convenience	Unspecified	Lifetime	417	8	M: 417	M: 8
Kenya[140]	2017	Lower middle	Sub-National	Kisumu, Nairobi, Mombasa, Kilifi	B3	A	Probability	Unspecified	Month	996	10		
Kenya[192]	2014	Lower middle	Sub-National	Nairobi, Mombasa	A1	B1	Unspecified	1 month	Month	690	41		
Kenya[193]	2014	Lower middle	City	Kisumu	A1	B1	Unspecified	1 month	Lifetime	151	34	M: 127<br>F: 24	M: 61<br>F: 42
Liberia[107]	2018	Low	Sub-National	Montserrado County, Grand Cape Mount, Grand Gedeh, Margibi	B2	A	RDS	12 months	12 months	515	24		
Liberia[108]	2013	Low	National		B3	B1	Convenience	Lifetime	12 months	300	35		

Country and reference	Study year	Income level	Geographic coverage	Location (if not national)	Literature grade	Method grade	Recruitment method	Recency of injecting drug use	Timeframe for STI diagnosis	Sample size (N)	Prev. (%)	Gender-specific sample size (N)	Gender-specific prev. (%)
Libya[194]	2010	Upper middle	City	Tripoli	A1	B1	Unspecified	1 month	Lifetime	250	12		
Mauritius[110]	2017	Upper middle	National		B3	A	RDS	Unspecified	12 months	500	7	M: 431<br>F: 62	M: 6<br>F: 11
Moldova (Republic of)[51]	2020	Upper middle	Sub-National	Chisinau, Balti, Tiraspol, Ribnita	B2	A	RDS	12 months	12 months	1377	4		
Mozambique[144]	2014	Low	Sub-National	Maputo and Nampula/ Nacala	A1	B1	RDS	Lifetime	12 months	492	27		
Myanmar[195]	2014	Lower middle	National		B3	A	RDS	1 month	12 months	3164	8	M: 3164	M: 8
Nepal[89]	2015	Lower middle	Sub-National	Eastern Terai	B3	B1	Probability	3 months	12 months	360	12	M: 360	M: 12
Nepal[92]	2012	Lower middle	Sub-National	Eastern Terai	B3	B1	Probability	3 months	Month	340	9	M: 340	M: 9
Nepal[93]	2011	Lower middle	Sub-National	Kathmandu Valley	B3	B1	RDS	3 months	12 months	340	10	M: 340	M: 10
Nigeria[149]	2020	Lower middle	National		B2	A	Probability	12 months	12 months	4414	21		
Occupied Palestinian Territory[196]	2010	Upper middle	Sub-National	East Jerusalem Governorate	B2	C	RDS	1 month	12 months	192	5	M: 192	M: 5
Pakistan[197]	2014	Lower middle	City	Bahawalpur	C	B1	Convenience	Unspecified	Month	60	58		
Serbia[123]	2014	Upper middle	Sub-National	Prishtina and Prizren	B3	B1	RDS	1 month	12 months	497	4	M: 497	M: 4
South Africa[198]	2013	Upper middle	Sub-National	Gauteng, KwaZulu-Natal, Western Cape Province	A1	A	Convenience	Lifetime	12 months	900	25	M: 726<br>F: 174	M: 24<br>F: 30
Syrian Arab Republic[199]	2014	Low	Sub-National	Damascus, Rif Damascus, Lattakia, Tartous	B2	B1	RDS	Unspecified	Lifetime	394	20	M: 147<br>F: 17	M: 14<br>F: 41
Tajikistan [4]	2018	Lower middle	National		B3	A	RDS	6 months	6 months	1916	9		
Tanzania[117]	2019	Lower middle	City	Unguja	B2	A	RDS	3 months	419	15			
Togo [200]	2015	Low	City	Lome	A1	C	Convenience	6 months	Month	164	17		
Chlamydia													
Azerbaijan[43]	2008	Upper middle	City	Sumgait	B3	B1	Convenience	1 month	Past year	150	2		
Canada[130]	2012	High	City	Prince George	B3	B1	Convenience	6 months	Lifetime	150	23		

Country and reference	Study year	Income level	Geographic coverage	Location (if not national)	Literature grade	Method grade	Recruitment method	Recency of injecting drug use	Timeframe for STI diagnosis	Sample size (N)	Prev. (%)	Gender-specific sample size (N)	Gender-specific prev. (%)
Canada[131]	2011	High income	Sub-National	Ontario	D	B1	Convenience	6 months	Lifetime	1323	21		
Latvia[50]	2007	High	City	Riga	B3	B1	RDS	Unspecified	Lifetime	407	4		
Puerto Rico[157]	2006	High	City	San Juan	B3	B1	RDS	12 months	Past year	600	2	M: 500<br>F: 70	M: 1<br>F: 10
Spain[201]	2011	High	City	Catalonia	A1	B1	Convenience	1 month	Lifetime	1345	1	M: 1000<br>F: 233	M: <1<br>F: 3
Ukraine[58]^	2009	Lower middle	National		B2	B1	Unspecified	1 month	Past year	3962	2		
United States[202]	2022	High	National		B2	A	RDS	12 months	12 months	7163	4		
United States[203]	2022	High	National		A	B2	Convenience	Lifetime	Lifetime	728	22		
United States[171]	2018	High	National		B3	B1	RDS	12 months	Past year	11437	3	M: 7891<br>F: 3449	M: 2<br>F: 5
United States[175]	2012	High	National		B3	B1	RDS	12 months	Past year	10300	2	M: 6892<br>F: 2823	M: 1<br>F: 4
United States[156]^	2009	High	National		B3	B1	RDS	12 months	Past year	10400	2	M: 8900<br>F: 2975	M: 1<br>F: 4
United States[157]^	2006	High	National		B3	B1	RDS	12 months	Past year	10500	4	M: 8850<br>F: 3038	M: 2<br>F: 8
Gonorrhoea													
Azerbaijan[43]	2008	Upper middle	City	Sumgait	B3	B1	Convenience	1 month	12 months	150	13		
Canada[130]	2012	High	City	Prince George	B3	B1	Convenience	6 months	Lifetime	150	12		
Canada[131]	2011	High	Sub-National	Ontario	D	B1	Convenience	6 months	Lifetime	1323	11		
Latvia[50]	2007	High	City	Riga	B3	B1	RDS	Unspecified	Lifetime	407	8		
Puerto Rico[156]	2009	High	City	San Juan	B3	B1	RDS	12 months	12 months	350	2		
Puerto Rico[157]	2006	High	City	San Juan	B3	B1	RDS	12 months	12 months	517	6	M: 317<br>F: 71	M: 7<br>W: 7
Spain[201]	2011	High	City	Catalonia	A1	B1	Convenience	1 month	Unspecified	1345	4	M: 1100<br>F: 235	M: 4<br>F: 3
Ukraine[58]^	2009	Lower middle	National		B2	B1		1 month	12 months	3962	4		
United States[202]	2022	High	National		B2	A	RDS	12 months	12 months	7050	4		
United States[203]	2022	High	National		A	B2	Convenience	Lifetime	Lifetime	728	15		
United States[171]	2018	High	National		B3	B1	RDS	12 months	12 months	11437	2.7	M: 7891<br>F: 3448	M: 2<br>F: 4
United States[175]	2012	High	National		B3	B1	RDS	12 months	12 months	10150	2	M: 7221<br>F: 2823	M: 2<br>F: 2
United States[156]^	2009	High	National		B3	B1	RDS	12 months	12 months	10800	2	M: 7450<br>F: 2233	M: 2<br>F: 3

Country and reference	Study year	Income level	Geographic coverage	Location (if not national)	Literature grade	Method grade	Recruitment method	Recency of injecting drug use	Timeframe for STI diagnosis	Sample size (N)	Prev. (%)	Gender-specific sample size (N)	Gender-specific prev. (%)
United States[157]^	2006	High	National		B3	B1	RDS	12 months	12 months	10714	7	M: 7857<br>F: 2857	M: 7<br>F: 7
Herpes simplex virus													
Canada[130]	2012	High	City	Prince George	B3	B1	Convenience	6 months	Lifetime	150	3		
Canada[131]	2011	High	Sub-National	Ontario	D	B1	Convenience	6 months	Lifetime	1323	11		
Latvia[50]	2007	High	City	Riga	B3	B1	RDS	Unspecified	Lifetime	407	3		
Puerto Rico[157]	2006	High	City	San Juan	B3	B1	RDS	12 months	Past year	500	2		
Spain[201]	2011	High	City	Catalonia	A1	B1	Convenience	1 month	Unspecified	1345	2	M: 1067<br>F: 237	M: 2<br>F: 4
Ukraine[58]^	2009	Lower middle	National		B2	B1	Unspecified	1 month	Past year	3962	2		
United States[202]	2022	High	National		B2	A	RDS	12 months	Lifetime	7111	5		
United States[203]	2022	High	National		A	B2	Convenience	Lifetime	Lifetime	728	6		
United States[171]	2018	High	National		B3	B1	RDS	12 months	12 months	11437	5	M: 7891<br>F: 3449	M: 2<br>F: 8
United States[174]	2015	High	National		A1	B2	Probability	Lifetime	Lifetime	323	11		
United States[175]	2012	High	National		B3	B1	RDS	12 months	Lifetime	10169	6	M: 7162<br>F: 2849	M: 4<br>F: 11
United States[156]^	2009	High	National		B3	B1	RDS	12 months	Past year	11800	1	M: 7200<br>F: 2300	M: 1<br>F: 2
United States[157]^	2006	High	National		B3	B1	RDS	12 months	Past year	13000	2	M: 7200<br>F: 2850	M: 2<br>F: 4
Human papillomavirus													
Canada[130]	2012	High	City	Prince George	B3	B1	Convenience	6 months	Lifetime	150	5		
Canada[131]	2011	High	Sub-National	Ontario	D	B1	Convenience	6 months	Lifetime	1323	4		
Spain[201]	2011	High	City	Catalonia	A1	B1	Convenience	1 month	Lifetime	1345	2	M: 1088<br>F: 235	M: 3<br>F: 14
United States[202]	2022	High	National		B2	A	RDS	12 months	12 months	7108	4		
United States[203]	2022	High	National		A	B2	Convenience	Lifetime	Lifetime	728	4		
United States[171]	2018	High	National		B3	B1	RDS	12 months	12 months	11437	4	M: 7891<br>F: 3449	M: 3<br>F: 6
United States[175]	2012	High	National		B3	B1	RDS	12 months	Lifetime	10087	5	M: 6775<br>F: 2856	M: 4<br>F: 7
Syphilis													
Canada[130]	2012	High	City	Prince George	B3	B1	Convenience	6 months	Lifetime	150	5		
Canada[131]	2011	High	Sub-National	Ontario	D	B1	Convenience	6 months	Lifetime	1323	3		
Puerto Rico[156]	2009	High	City	San Juan	B3	B1	RDS	12 months	12 months	467	3	M: 300<br>F: 73	M: 2<br>F: 11
Puerto Rico[157]	2006	High	City	San Juan	B3	B1	RDS	12 months	12 months	500	5	M: 375<br>F: 67	M: 4<br>F:15

Country and reference	Study year	Income level	Geographic coverage	Location (if not national)	Literature grade	Method grade	Recruitment method	Recency of injecting drug use	Timeframe for STI diagnosis	Sample size (N)	Prev. (%)	Gender-specific sample size (N)	Gender-specific prev. (%)
Spain[201]	2011	High	City	Catalonia	A1	B1	Convenience	1 month	Unspecified	1345	3	M: 1120<br>F: 236	M: 3<br>F: 6
Ukraine[204]^	2017	Lower middle	National		A1	A	RDS	1 month	Lifetime	10076	2		
United States[171]	2018	High	National		B3	B1	RDS	12 months	12 months	11437	2	M: 7891<br>F: 3449	M: 2<br>F 2
United States[174]	2015	High	National		A1	B2	Probability	Lifetime	Lifetime	323	9	M: 181<br>F: 142	M: 9<br>F: 9
United State [175]	2012	High	National		B3	B1	RDS	12 months	12 months	10117	2		
United States[156]^	2009	High	National		B3	B1	RDS	12 months	12 months	12400	1	M: 7800<br>F: 2300	M: 1<br>F: 2
United States[157]^	2006	High	National		B3	B1	RDS	12 months	12 months	9625	4	M: 8600<br>F: 2175	M: 3<br>F: 4
Trichomoniasis													
Azerbaijan[43]	2008	Upper middle	City	Sumgait	B3	B1	Convenience	1 month	12 months	150	9		
Ukraine[58]^	2009	Lower middle	National		B2	B1	Unspecified	1 month	12 months	3962	2		

Notes. ^The studies disaggregated results by geographic region; for brevity, only the national estimate is presented here.

References

1. Degenhardt L, Webb P, Colledge-Frisby S, et al. Epidemiology of injecting drug use, prevalence of injecting-related harm, and exposure to behavioural and environmental risks among people who inject drugs: a systematic review. *Lancet Glob Health* 2023; **11**(5): e659-e72.
2. World Health Organization. Global progress report on HIV, viral hepatitis and sexually transmitted infections, 2021. Accountability for the global health sector strategies 2016–2021: actions for impact. Web Annex 2. Data methods. 2021.
3. Chokmorova U, Ismailova A, Bubusara S, et al. IBBS 2013 Report. 2013.
4. Republic of Tajikistan Ministry of Health and Social Protection of the Population. Integrated biological and behavioral study on HIV infection among people who inject drugs in the Republic of Tajikistan, 2018: Republican Center for Prevention and Control of AIDS, 2019.
5. Beyrer C, Patel Z, Stachowiak JA, et al. Characterization of the emerging HIV type 1 and HCV epidemics among injecting drug users in Dushanbe, Tajikistan. *AIDS Research & Human Retroviruses* 2009; **25**(9): 853-60.
6. Mburu G, Chhoun P, Chann N, Tuot S, Mun P, Yi S. Prevalence and risk factors of HIV infection among people who inject drugs in Cambodia: findings from a national survey. *Subst Abuse Treat Prev Policy* 2019; **14**(1): 42.
7. Chhoun P, Tuot S, Yi S, et al. Syphilis infection among people who use and inject drugs in Cambodia: a cross-sectional study using the respondent-driven sampling method. *International Journal of STD and AIDS* 2020; **31**(9): 832-40.
8. Phalkun Mun ST, Boumony Kao, Navy Chann, Pheak Chhoun, and Siyan Yi. National Integrated Biological and Behavioral Survey and

Population Size Estimation among People Who Use and Inject Drugs in Cambodia (2017), 2018.
9. Jiang H, Lu R, Zhou C, et al. Trends of HIV, hepatitis C virus and syphilis seroprevalence among injection and non-injection drug users in southwestern China, 2010-2017. *AIDS Care - Psychological and Socio-Medical Aspects of AIDS/HIV* 2020.
10. Shi Y, Yang Y, Wang Y, et al. Prevalence and associated factors of Treponema pallidum infection in a rural area of southwestern China. *BMC Public Health* 2020; **20**(1): 824.
11. Deng X. Analysis of HIV and HPC in drug users (in Mandarin). 2014.
12. Zhu XH, Xun JP, Gao L, Peng JJ. Monitoring and Analysis of Clinical Blood in 613 MMT Clients. 2010.
13. Bai Y, Lai WS, Wei L. Prevalence of HIV, HCV and Syphilis Infection at Methadone Maintenance Treatment Clinic in Liuzhou City. 2009.
14. Gao LF, Yang J, Li SJ. Detection and Analysis of HIV, HBV, Syphilis and HCV Infections of Methadone Clinic in Jinchang City. 2010.
15. Han XJ, Xu YX, Jiang HM, He J, Tan B, Cao FB. Infection of HIV,HBV,HCV and Syphilis in 300 Drug Abusers in Guiyang City. 2010.
16. Wang J, Yang XJ. Prevalence of HIV, HCV Infection and Syphilis among Drug Users at Methadone Maintenance Clinics in Anshun. 2010.
17. Xia L, Zhang QS, Deng XL, et al. Prevalence of HIV, HCV and Syphilis among Heroin Addicts at Methadone Maintenance Treatment Clinic in Shenzhen City. 2010.
18. Wang M, Mao W, Zhang L, et al. Methadone maintenance therapy and HIV counseling and testing are associated with lower frequency of risky behaviors among injection drug users in China. *Substance Use & Misuse* 2015; **50**(1): 15-23.
19. Wu J, Huang J, Xu D, Lu C, Deng X, Zhou X. Infection status and risk factors of HIV, HBV, HCV, and syphilis among drug users in Guangdong, China--a cross-sectional study. *BMC Public Health* 2010; **10**: 657.
20. Tan, Zhou. 南宁市2007—2008年静脉吸毒人群艾滋病性病血清检测结果分析. 2010.
21. Yao W, Jiang ZH, Jia SG, Yang H, Liao KK. Prevalence of HIV, HCV and Syphilis Infection at Methadone Maintenance Treatment Clinic in Mianyang City. 2008.
22. Dong G, Jia H. HIV, HCV and Syphilis Infection among Drug Users in Luzhou City. 2009.
23. Chen LF. Survey of Infectious Status of HIV, HBV, Syphilis and HCV on 459 Drug Users. 2009.
24. Jia Y, Lu F, Zeng G, et al. Predictors of HIV infection and prevalence for syphilis infection among injection drug users in China: Community-based surveys along major drug trafficking routes. *Harm Reduction Journal* 2008; **5 (no pagination)**(29).

25. Ministry of Health and the National AIDS Commission Indonesia. 2013 Sero-Surveillance Survey And 2013 Rapid Behavioral Survey. 2013.
26. Morineau G, Bollen LJ, Syafitri RI, Nurjannah N, Mustikawati DE, Magnani R. HIV prevalence and risk behaviours among injecting drug users in six Indonesian cities implications for future HIV prevention programs. *Harm Reduction Journal* 2012; **9**: 37.
27. Sports MoHa. Myanmar IBBS and Population size estimates among PWID 2017-2018, 2019.
28. WHO. HIV Sentinel Sero-Surveillance Survey Report 2014. 2015.
29. AIDS Data Hub. HIV Sentinel Sero-Surveillance Survey Report 2012. 2013.
30. WHO. HIV Sentinel Sero-Surveillance Survey Report 2011. 2012.
31. WHO. HIV Sentinel Sero-Surveillance Survey Report 2010. 2011.
32. National AIDS Program Myanmar. HIV Sentinel Sero-Surveillance Survey Report 2009. 2010.
33. WHO. HIV Sentinel Sero-Surveillance Survey Report 2008. 2009.
34. HIV and AIDS Data Hub for Asia-Pacific. IHBSS Philippines. 2013.
35. HIV and AIDS Data Hub for Asia-Pacific. IHBSS Philippines. 2011.
36. Shi MD, Zhang KX, Tsai LY. Injecting drug users (IDUs) prevalence of hepatitis virus, HIV and syphilis in Southern Taiwan. *Hepatology International* 2013; **7**: S116.
37. Pansuwan N, Wisawakam P, Saengwanloy O, Jittakot Y, Pawa D. The 2012 Integrated Behavior and Biological Surveillance (IBBS) of HIV, Sexually Transmitted Infections and Associated Risk Behaviors among Injecting Drug Users. 2012.
38. National Center for Infectious Diseases of the Ministry of Health of the Republic of Armenia. Integrated Bio-Behavioral Surveillance Surveys and Key Population Size Estimations Among People Who Inject Drugs, Female Sex Workers, Men Who Have Sex With Men, and Transgender Persons, 2021.
39. National Center for AIDS Prevention of Ministry of Health of Armenia. INTEGRATED BIOLOGICAL-BEHAVIORAL SURVEILLANCE SURVEY AMONG PEOPLE WHO INJECT DRUGS, FEMALE SEX WORKERS, MEN WHO HAVE SEX WITH MEN AND TRANSGENDER PERSONS, 2018.
40. Grigoryan S, Hakobyan A, Papoyan A, et al. Results from the HIV Biological and Behavioural Surveillance in the Republic of Armenia. 2013.
41. Global Fund. Armenia IBBS. 2012.
42. WHO. The report on results of a surveillance survey on knowledge, risks and prevalence of HIV and sexually and parenterally transmitted infections in most-at-risk populations in Azerbaijan. 2012.
43. Ministry of Health the Republic of Azerbaijan. Prevalence of HIV, Hepatitis and Syphilis, and Behavioural Risk Factors Among Most-At-Risk Groups in the Republic of Azerbaijan. 2008.
44. Arkad'yevna YK. ПОВЕДЕНЧЕСКИЕ ОСОБЕННОСТИ И УРОВЕНЬ ЗНАНИЙ ПО ПРОБЛЕМЕ ВИЧ/СПИД СРЕДИ ПОТРЕБИТЕЛЕЙ ИНЪЕКЦИОННЫХ НАРКОТИКОВ. 2015.
45. Bacak V, Dominkovic Z. Report on behavioral and biological surveillance among injection drug users in Bosnia and Herzegovina, 2009: a respondent driven sampling survey, 2009. 2012.
46. Sekera JC, Frybert J. Analysis of drug-related infectious diseases in people who inject drugs - Pilsen Region, 2003-2018. *Cent Eur J Public Health* 2022; **30**(1): 13-9.
47. Uuskula A, McMahon JM, Raag M, et al. Emergent properties of HIV risk among injection drug users in Tallinn, Estonia: synthesis of individual and neighbourhood-level factors. *Sexually Transmitted Infections* 2010; **86 Suppl 3**: iii79-84.
48. Curatio International Foundation, Public Union Bemoni. Bio-behavioral surveillance surveys among injecting drug users in Georgia (Tbilisi, Batumi, Zugdidi, Telavi, Gori, 2008 - 2009). 2009.
49. Gyarmathy VA, Neaigus A, Ujhelyi E. Vulnerability to drug-related infections and co-infections among injecting drug users in Budapest, Hungary. *European Journal of Public Health* 2009; **19**(3): 260-5.
50. Expanding Network for Comprehensive and Coordinated Action on HIV/AIDS prevention among IDUs and Bridging Population (ENCAP). Prevalence of HIV and other infections and risk behaviour among Injecting Drug Users in Latvia, Lithuania and Estonia in 2007, 2009. 2009.
51. Fund TG. Integrated Biological-Behavioral Surveillance Survey Among Female Sex Workers, People Who Inject Drugs and Men Who Have Sex With Men in the Republic of Moldova. Chisinau, 2020.
52. Global Fund. Integrated Bio-Behavioural Study in key populations at higher risk: key indicators. 2013.
53. Reitox National Focal Point, Malczewski A, Kidawa M, Struzik M, Strzelecka A. 2010 National Report (2009 data) to the EMCDDA. "Poland" New Development, Trends and in-depth information on selected issues, 2011.

54. Vagaitseva N, Demyanenko E. Evaluation of HIV, HBV, HCV, syphilis and related risk behaviour prevalence among the injecting drug users (IDU) in the City of Barnaul, Russia. *Sexually Transmitted Infections Conference: STI and AIDS World Congress* 2013; **89**(no pagination).
55. Platt L, Sutton A, Vickerman P, et al. Measuring risk of HIV and HCV among injecting drug users in the Russian Federation. *European Journal of Public Health* 2009; **19**(4): 428-33.
56. Titar I, Salnikov S, Ogorodnik S, et al. Report of the 2020 Integrated Biobehavioral Study of People Who Inject Drugs. *Center of Public Health of the Ministry of Health of Ukraine* 2021.
57. Barska G, Sazonov JO. Survey Results 2015. Monitoring Behaviour and HIV Prevalence among People who use Injectable Drugs and their Sexual Partners, 2016.
58. Pohorila N, Taran Y, Kolodiy I, Diyeva T. Behavior monitoring and HIV-infection prevalence among injection drug users. 2010.
59. Rusch ML, Lozada R, Pollini RA, et al. Polydrug use among IDUs in Tijuana, Mexico: correlates of methamphetamine use and route of administration by gender. *Journal of Urban Health* 2009; **86**(5): 760-75.
60. Baumbach JP, Foster LN, Mueller M, et al. Seroprevalence of select bloodborne pathogens and associated risk behaviors among injection drug users in the Paso del Norte region of the United States-Mexico border. *Harm Reduction Journal* 2008; **5**: 33.
61. Brouwer K, Lozada R, Cornelius W, et al. Deportation along the U.S.-Mexico border: Its relation to drug use patterns and accessing care. *Journal of Immigrant and Minority Health* 2009; **11**(1): 1-6.
62. Ministry of Public Health and Social Assistance. Encuesta de Vigilancia de Comportamiento Sexual en Poblaciones Clave en Nicaragua. 2021.
63. Ministerio de Salud Nicaragua. Estudio de Vigilancia de Comportamiento Sexual y prevalencia del VIH y sífilis en poblaciones vulnerables y en mayor riesgo al VIH. 2014.
64. Pant Pai N, Behlim T, Landry G, Savard P, Joseph L, Potter M. Will a quad point-of-care multi plexed assay for HIV, HCV, HBV, syphilis be feasible, accurate and preferred by injection drug users: A pilot study from Montreal, Canada. *Canadian Journal of Infectious Diseases and Medical Microbiology* 2013; **24**: 88A-9A.
65. Cari EV, Henderson JT, Burgess D, Porterfield J, Thornton A, Leedy N. Sexually transmitted infections and hepatitis c in people who inject drugs. *Sexually Transmitted Diseases* 2020; **47**(9 SUPPL 2): S172.
66. National AIDS Control Program Afghanistan. Integrated Biological & Behavioral Surveillance (IBBS) in Selected Cities of Afghanistan. 2012.
67. Todd CS, Nasir A, Stanekzai M, et al. Prevalence and correlates of HIV, syphilis, and hepatitis B and C infection and harm reduction program use among male injecting drug users in Kabul, Afghanistan: A cross-sectional assessment. *Harm Reduction Journal* 2011; **8**: 22.
68. Ruisenor-Escudero H. Injecting drug use in Afghanistan: Risk factors for HIV, HCV, STIS, injecting drug practices, and outcomes of an opiate substitution therapy program. *Dissertation Abstracts International: Section B: The Sciences and Engineering* 2014; **75**(2-B(E)): No Pagination Specified.
69. Todd CS, Nasir A, Raza Stanekzai M, et al. Prevalence and correlates of syphilis and condom use among male injection drug users in four Afghan cities. *Sexually Transmitted Diseases* 2010; **37**(11): 719-25.
70. The Global Fund. Integrated Biological and Behavioural Survey (IBBS) among Key Populations at High Risk of HIV in Bangladesh, 2021.
71. Azim T, Rahman M, Rahman M, et al. National HIV Serological Surveillance, 2011 Bangladesh. 2011.
72. Azim T, Rahman M, Alam MS, et al. Bangladesh moves from being a low-prevalence nation for HIV to one with a concentrated epidemic in injecting drug users. *International Journal of STD & AIDS* 2008; **19**(5): 327-31.
73. Azim T, Chowdhury EI, Reza M, et al. Prevalence of infections, HIV risk behaviors and factors associated with HIV infection among male injecting drug users attending a needle/syringe exchange program in Dhaka, Bangladesh. *Substance Use & Misuse* 2008; **43**(14): 2124-44.
74. Ghosh I, Ghosh P, Bharti AC, Mandal R, Biswas J, Basu P. Prevalence of human papillomavirus and co-existent sexually transmitted infections among female sex workers, men having sex with men and injectable drug abusers from eastern India. *Asian Pacific Journal of Cancer Prevention: Apjcp* 2012; **13**(3): 799-802.

75. Panda S, Roy T, Pahari S, et al. Alarming epidemics of human immunodeficiency virus and hepatitis C virus among injection drug users in the northwestern bordering state of Punjab, India: Prevalence and correlates. *International Journal of STD and AIDS* 2014; **25**(8): 596-606.
76. FHI 360. India: IBBA 2009-2010. 2011.
77. Kermode M, Armstrong G, Medhi GK, Humtsoe C, Langkham B, Mahanta J. Sexual behaviours of men who inject drugs in Northeast India. *Harm Reduction Journal* 2015; **12**: 4.
78. Mahanta J, Medhi GK, Paranjape RS, et al. Injecting and sexual risk behaviours, sexually transmitted infections and HIV prevalence in injecting drug users in three states in India. *Aids* 2008; **22**(SUPPL. 5): S59-S68.
79. Goswami P, Medhi GK, Armstrong G, et al. An assessment of an HIV prevention intervention among people who inject drugs in the states of Manipur and Nagaland, India. *International Journal of Drug Policy* 2014; **25**(5): 853-64.
80. Rahimi-Movaghar A, Razaghi EM, Sahimi-Izadian E, Amin-Esmaeili M. HIV, hepatitis C virus, and hepatitis B virus co-infections among injecting drug users in Tehran, Iran. *International Journal of Infectious Diseases* 2010; **14**(1): e28-33.
81. Republic of Maldives. Biological and Behavioral Survey (BBS) on HIV and AIDS 2008. 2008.
82. National Center for AIDS and STD Control (NCASC). National Integrated Biological and Behavioral Surveillance (IBBS) Survey among People Who Inject Drugs (PWID) in Nepal - 2020, 2020.
83. HIV NSPo. National Integrated Biological and Behavioral Surveillance (IBBS)

Survey among People Who Inject Drugs (PWID) in Nepal-2020, 2020.
84. Ministry of Health and Population. Integrated Biological and Behavioral Surveillance (IBBS) Survey among People Who Inject Drugs (PWID) in Kathmandu Valley, 2017.
85. Ministry of Health. Integrated Biological and Behavioral Surveillance (IBBS) Survey among Female Injecting Drug Users (FIDUs) in Pokhara Valley, 2017.
86. Ministry of Health. Integrated Biological and Behavioural Surveillance (IBBS) Surveys among People Who Inject Drugs (PWID) in West to Far West Terai Districts, 2017.
87. Deuba K, Shrestha U, Shrestha MK, et al. Hepatitis C and HIV co-infection and related risk determinants among women who inject drugs in the capital city of Nepal. *Journal of the International AIDS Society* 2019; **22**(Supplement 5).
88. Health Mo. Integrated Biological and Behavioral Surveillance (IBBS) Survey among Female Injecting Drug Users in Kathmandu Valley, 2016.
89. National Centre for AIDS and STD Control (NCASC). Integrated Biological and Behavioral Surveillance (IBBS) Survey among People Who Inject Drugs (PWID- Male) in the Eastern Terai Highway Districts (Jhapa, Morang and Sunsari) of Nepal. 2015.
90. National Centre for AIDS and STD Control (NCASC). Integrated Biological and Behavioral Surveillance (IBBS) Survey among People Who Inject Drugs (PWID) in Kathmandu Valley. 2015.
91. National Centre for AIDS and STD Control (NCASC). Integrated Biological and Behavioral Surveillance (IBBS) Survey among People Who Inject Drugs (PWIDs) in Pokhara Valley. 2015.
92. Ministry of Health and Population Nepal. Integrated Biological and Behavioral Surveillance (IBBS) Survey among People who Inject Drugs (PWIDs) in Eastern Terai Highway Districts of Nepal. 2012.
93. Ministry of Health and Population Nepal. Integrated Biological and Behavioral Surveillance (IBBS) Survey among Injecting Drugs Users in Kathmandu Valley, Nepal. 2011.
94. Ministry of Health and Population Nepal. Integrated Biological and Behavioral Surveillance Survey (IBBS) among Male Injecting Drug Users (IDUs) in the Eastern Terai of Nepal Round IV. 2009.
95. Ministry of Health and Population Nepal. Integrated Biological and Behavioral Surveillance Survey (IBBS) among Injecting Drug Users in Kathmandu Valley 2009.
96. Ministry of Health and Population Nepal. Integrated Biological and Behavioral Surveillance Survey (IBBS) among Injecting Drug Users in Pokhara Valley. 2009.
97. Ministry of Health and Population Nepal. Integrated Biological and Behavioral Surveillance Survey (IBBS) among Male Injecting Drug Users (IDUs) in Western to Far-Western Terai of Nepal. 2009.
98. Platt L, Vickerman P, Collumbien M, et al. Prevalence of HIV, HCV and sexually transmitted infections among injecting drug users in Rawalpindi and Abbottabad, Pakistan: evidence for an emerging injection-related HIV epidemic. *Sexually Transmitted Infections* 2009; **85 Suppl 2**: ii17-22.
99. Frontiers NSACPaM. INTEGRATED BIOLOGICAL AND

BEHAVIOURAL SURVEILLANCE (IBBS)

SURVEY AMONG KEY POPULATIONS AT

HIGHER RISK OF HIV IN SRI LANKA, 2018.
100. Global Fund. IBBS Survey in Sri Lanka 2015.
101. Ministere de la Sante Publique et de la Lutte Contre le Sida. ENQUETE NATIONALE DE SEROPREVALENCE ET DE SURVEILLANCE DES COMPORTEMENTS FACE AU VIH, HEPATITES VIRALES ET SYPHILIS AU BURUNDI CHEZ LES CONSOMMATEURS DE DROGUES INJECTABLES. 2022.
102. Le Fonds mondial. Enquête sur la prévalence des IST/VIH et des Comportements à risques chez les Jeunes et les Populations clés aux Comores en 2020, 2020.
103. Bouscaillou J, Evanno J, Prouté M, et al. Prevalence and factors associated with HIV and tuberculosis in people who use drugs in Abidjan, Ivory Coast. *International Journal of Drug Policy* 2016: 8.
104. HEALTH MO. BIO- SURVEILLANCE SURVEY BEHAVIORAL AMONG POPULATIONS KEYS AT RISK FOR HIV INFECTION IN 14 PROVINCES, 2018-201, 2020.
105. Demissie M, Johnston LG, Muleta M, et al. Prevalence of HIV and other infections and injection behaviours among people who inject drugs in Addis Ababa, Ethiopia. *Afr J AIDS Res* 2018; **17**(3): 259-64.
106. Tun W, Sheehy M, Broz D, et al. HIV and STI prevalence and injection behaviors among people who inject drugs in Nairobi: results from a 2011 bio-behavioral study using respondent-driven sampling. *AIDS and Behavior* 2015; **19 Suppl 1**: S24-35.
107. The Global Fund. Integrated Bio-Behavioural Surveillance Survey Report Of Key Populations In Liberia (IBBSS, 2018), 2019.
108. Government of Liberia. Integrated Bio-Behavioural Surveillance Survey (IBBSS) among MARPs in Liberia. 2013.
109. African Development Bank Group. Etude comportementale et biologique chez les consommateurs de drogues injectables dans les zones urbaines à Madagascar-2012. 2012.
110. Ministry of Health and Quality of Life. A Respondent Driven Survey (RDS) among People Who Inject Drugs [PWIDs] in the Island of Mauritius, 2017.
111. Republic of Mauritius. A Respondent Driven Survey (RDS) among

People Who Inject Drugs [PWIDs] in the Island of Mauritius, 2018.
112. Ministry of Health and Quality of Life. Integrated Biological Behavioral Survey Among People Who Inject Drugs in Mauritius. 2011.
113. Johnston L, Saumtally A, Corceal S, Mahadoo I, Oodally F. High HIV and hepatitis C prevalence amongst injecting drug users in Mauritius: findings from a population size estimation and respondent driven sampling survey. *International Journal of Drug Policy* 2011; **22**(4): 252-8.
114. Tun W, Vu L, Adebajo SB, et al. Population-based prevalence of hepatitis B and C virus, HIV, syphilis, gonorrhoea and chlamydia in male injection drug users in Lagos, Nigeria. *International Journal of STD & AIDS* 2013; **24**(8): 619-25.
115. Vel B. SEYCHELLES BIOLOGICAL AND BEHAVIOURAL SURVEILLANCE OF HEROIN USERS 2017: ROUND ONE FINAL REPORT, 2018.
116. Bibi J, Faure J, Johnston L, Sinon F, Isnard R, Mangroo G. Injection Drug Use in the Republic of Seychelles, 2011. Integrated Biological and Behavioral Surveillance Survey - Round 1, 2011.
117. Zanzibar Integrated HIV Tuberculosis and Leprosy Programme (ZIHHTLP). Integrated behavioural and biological surveillance survey among key populations in Zanzibar, 2018-2019. Zanzibar, 2019.
118. National AIDS Control Programme (NACP). Integrated Bio-Behavioral Surveillance Survey among People who Inject Drugs in Dar es Salaam, 2017, 2018.
119. Boci A, Hallkaj E, Bani R. HIV and HCV prevalence and risky behaviors among needle exchange program users in Albania. *Sexually Transmitted Infections Conference: STI and AIDS World Congress* 2013; **89**(no pagination).
120. Zimmermann R, Krings A, Steffen G. DRUCK 2.0-Pilot - DRUCK 2.0 - Pilotierung eines Surveillancesystems zu durch Blut und sexuell übertragenen Infektionen bei Drogengebrauchenden. Berlin, 2023.
121. Institute for Public Health of the Republic of Macedonia. Report on the Bio Behavioural Study and Population Size Estimates of People Who Inject Drugs in Skopje, Republic of Macedonia, 2017, 2017.
122. Borovcanin N, Todorovic M, Borovcanin M, Jovanovic M, Ristanovic E, Balint B. The use of complementary serological and molecular testing for blood-borne pathogens and evaluation of socio-demographic characteristics of intravenous drug users on substitution therapy from Sumadia district of Serbia. *Vojnosanitetski Pregled* 2019; **76**(6): 587-92.

123. National Institute of Public Health. HIV Integrated Behavioral and Biological Surveillance Surveys-Kosovo. 2014.
124. Kilibarda B, Simic D, Baros S, Ministry of Health, Brandic I. National Report on Drug Situation in Serbia, 2015.
125. Read P, Wareing H, De Dassel J, et al. Responding to syphilis is vulnerable populations: point of care testing in people who use drugs and experiencing homelessness. 25th IUSTI World Congress. Sydney, Australia; 2024.
126. Agramunt S, Price O, Lenton S. Sexual health behaviours in a sample of Australians who regularly inject drugs, 2023. *Drug Trends Bulletin Series Sydney: National Drug and Alcohol Research Centre, UNSW Sydney* 2024.
127. Peacock A, Uporova, J., Karlsson, A., Gibbs, D., Swanton, R., Kelly, G., Price, O., Bruno, R., Dietze, P., Lenton, S., Salom, C., Degenhardt, L., & Farrell, M. Australian Drug Trends 2019: Key Findings from the National Illicit Drug Reporting System (IDRS), 2019.
128. Plan Benin. Benin Consommateurs de Drogues Injectables (CDI). 2015.
129. Platt L, Stengel CM, Nkurunziza M, et al. Assessing risk of HIV and hepatitis C among people who inject drugs in East Africa: Findings from a rapid assessment. *J Viral Hepat* 2019; **26**(7): 926-9.
130. Shoemaker M, Taylor L, Callaghan R. Prince George 2012 I-Track Survey Results: Findings and Discussion, 2013.
131. Millson P, White S, Leonard L, Public Health Agency of Canada. Enhanced Surveillance of Risk Behaviours and Prevalence of HIV and Hepatitis C among People who Inject Drugs, 2016.
132. Zhou YH, Yao ZH, Liu FL, et al. High prevalence of HIV, HCV, HBV and co-infection and associated risk factors among injecting drug users in Yunnan Province, China. *PLoS ONE* 2012; **7 (8) (no pagination)**(e42937).
133. Cavlek TV, Maric J, Katicic L, Kolaric B. Hepatitis C virus antibody status, sociodemographic characteristics, and risk behaviour among injecting drug users in Croatia. *Central European Journal of Public Health* 2011; **19**(1): 26-9.
134. USAID. Rapport Annuel 2014, 2015.
135. Deyessa N, Senbete B, Abdo A, Mundia BM. Population estimation and harm reduction among people who inject drugs in Addis Ababa, Ethiopia. *Harm Reduction Journal* 2020; **17**(1): 61.
136. Calin R, Massari V, Pialoux G, et al. Acceptability of on-site rapid HIV/HBV/HCV testing and HBV vaccination among three at-risk populations in distinct community-healthcare outreach centres: the ANRS-SHS 154 CUBE study. *BMC Infect Dis* 2020; **20**(1): 851.
137. Noroozi M, Armoon B, Ghisvand H, et al. Prevalence and risk factors for injection site skin infections among people who inject drugs (PWID) in Tehran. *Journal of Cosmetic Dermatology* 2018.
138. Hosseini M, SeyedAlinaghi SA, Kheirandish P, et al. Prevalence and correlates of co-infection with human immunodeficiency virus and hepatitis C virus in male injection drug users in Iran. *Archives of Iranian Medicine* 2010; **13**(4): 318-23.
139. Population Services International. HIV and TB TRaC study evaluating risk behaviors associated with HIV transmission and utilization of HIV prevention and HIV/TB co-infection prevention among IDUs. Round one. 2010.
140. National AIDS & STI Control Programme MoH. Third National Behavioural Assessment of Key Populations in Kenya POLLING BOOTH SURVEY REPORT, 2018.
141. Robert Heimer KK, Forrest Crawford, Fatma M. Shebl, Russell Barbour, Danielle Khouri, Jacques Mokhbat. Project CROSSROADS Size Estimation, Risk Behavior Assessment, and Disease Prevalence in Populations at High Risk for HIV Infection in Lebanon, 2015.
142. Disease Control Division Ministry of Health Malaysia. Integrated Biological and Behavioral Surveillance Survey 2017, 2019.
143. Lechuga J, Ramos R, Ludwig-Barron N, et al. Social and environmental determinants influencing injection drug use and HIV risk among two sister cities on the US-Mexico border: a comparative cross-sectional study, 2016-2018. *Harm Reduct J* 2023; **20**(1): 84.
144. Boothe MAS, Comé C, Semá Baltazar C, et al. High burden of self-reported sexually transmitted infections among key populations in Mozambique: the urgent need for an integrated surveillance system. *BMC Infect Dis* 2020; **20**(1): 636.
145. Semá Baltazar C, Horth R, Boothe M, et al. High prevalence of HIV, HBsAg and anti-HCV positivity among people who injected drugs: results of the first bio-behavioral survey using respondent-driven sampling in two urban areas in Mozambique. *BMC Infect Dis* 2019; **19**(1): 1022.

146. National INstitute of Health (INS) of the Ministry of Health (MISAU). The Integrated Biological and Behavioral Survey Among People Who Inject Drugs, Mozambique, 2014

Final Report, 2017.
147. Boothe MAS, Semá Baltazar C, Sathane I, et al. Young key populations left behind: The necessity for a targeted response in Mozambique. *PLoS One* 2021; **16**(12): e0261943.
148. Nepal: National Centre for AIDS and STD Control. Mapping & Size Estimation of Most-At-Risk-Population in Nepal, 2011, Injecting Drug Users. 2011.
149. Federal Republic of Nigeria Federal Ministry of Health. Integrated Biological & Behavioural Surveillance Survey (IBBSS) among Key Populations in Nigeria, 2020.
150. Chatty A, AbuRabie R, Dibeh S, et al. HIV Bio-Behavioural Suvery among Injecting Drug Users in the East Jerusalem Governorate, 2010, 2010.
151. AP Consultancies Bridge Consultants Foundation. Integrated Behavioural and Biological Surveillance, among Most at Risk Population IBBS Study – Punjab 2014 2015.
152. Emmanuel F, Salim M, Akhtar N, Arshad S, Reza TE. Second-generation surveillance for HIV/AIDS in Pakistan: results from the 4th round of Integrated Behavior and Biological Survey 2011–2012. 2013.
153. National AIDS Control Program. HIV Second Generation Surveillance in Pakistan - National Report Round III. 2008.
154. Saleem NH, Adrien A, Razaque A. Risky sexual behavior, knowledge of sexually transmitted infections and treatment utilization among a vulnerable population in Rawalpindi, Pakistan. *Southeast Asian Journal of Tropical Medicine & Public Health* 2008; **39**(4): 642-8.
155. Collazo EM. Healthcare Service Access, Sexual Aggression Experiences, and HIV-Related Risk Behaviors among Puerto Rican Female Intravenous Drug Users. ProQuest LLC: Indiana University; 2015.
156. Centers for Disease Control and Prevention. HIV Infection and Risk, Prevention, and Testing Behaviors Among Injecting Drug Users - National HIV Behavioral Surveillance System, 20 U.S. Cities, 2009. *Morbidity and Mortality Weekly Report* 2014; **63**(6).
157. Centers for Disease Control and Prevention. Risk, Prevention, and Testing Behaviors Related to HIV and Hepatitis Infections: National HIV Behavioral Surveillance System Injecting Drug Users May 2005-February 2006. 2011.
158. Platt L, Rhodes T, Hickman M, et al. Changes in HIV prevalence and risk among new injecting drug users in a Russian city of high HIV prevalence. *JAIDS Journal of Acquired Immune Deficiency Syndromes* 2008; **47**(5): 623-31.
159. UNAIDS. Population Size Estimation of Key Populations. 2013.
160. Antuori A, Gonzalez-Gomez S, Saludes V, et al. Evaluation of the Xpert HCV VL Fingerstick point-of-care assay and dried blood spot HCV-RNA testing as simplified diagnostic strategies among people who inject drugs in Catalonia, Spain. *International Journal of Drug Policy* 2020; **80**: 102734.
161. REDAN Study. Monitoring of HIV, HCV and its determinants in people who inject drugs using Damage Reduction Centers in Catalonia, 2019.
162. Folch C, Reyes-Urena J, Casabona J, et al. The hepatitis C care cascade among people who inject drugs accessing harm reduction services in Catalonia: Major gaps for migrants. *International Journal of Drug Policy* 2021; **90**: 103057.
163. Cinta Folch JC, Xavier Majó, Mercè Meroño, Victoria González, Joan Colom, M. Teresa Brugal, Albert Espelt. Women who inject drugs and violence: Need for an integrated response. 2021.
164. Pares-Badell O, Espelt A, Folch C, et al. Undiagnosed HIV and Hepatitis C infection in people who inject drugs: From new evidence to better practice. *Journal of Substance Abuse Treatment* 2017; **77**: 13-20.
165. Folch C, Casabona J, Espelt A, et al. High Prevalence and Incidence of HIV and HCV Among New Injecting Drug Users With a Large Proportion of Migrants--Is Prevention Failing? *Subst Use Misuse* 2016; **51**(2): 250-60.
166. Yen YF, Yen MY, Lin T, et al. Prevalence and factors associated with HIV infection among injection drug users at methadone clinics in Taipei, Taiwan. *BMC Public Health* 2014; **14**: 682.
167. Zule WA, Latypov A, Otiashvili D, Bangel S, Bobashev GV. Feasibility of needle and syringe programs in Tajikistan distributing low dead space needles. *Harm Reduct J* 2018; **15**(1): 44.
168. Kpelly E, Schauder S, Bohm MK, Sounga D, Moukouta C. Profiles and health risks (STIs, HCV, HIV) of injecting drug users. *Rev Epidemiol Sante Publique* 2022; **70**(6): 299-304.
169. Tunisia Ministry of Health TAfIaOoH. Enquête sérocomportementale du VIH et des hépatites virales C auprès des usagers de drogues injectables en Tunisie. 2015.

170. Glick SN, Tinsley J, Golden MR, Klein KS. Increasing Heroin-Methamphetamine (Goofball) Use and Related Morbidity Among Seattle Area People Who Inject Drugs. *American Journal on Addictions* 2021; **30**(2): 183-91.
171. Prevention CfDCa. HIV Infection Risk, Prevention, and Testing Behaviors among Persons Who Inject Drugs—National HIV Behavioral Surveillance: Injection Drug Use, 23 U.S. Cities, 2018. HIV Surveillance Special Report 24. http://www.cdc.gov/hiv/library/reports/hiv-surveillance.html.: Centers for Disease Control and Prevention, 2020.
172. Rushmore J, Buchacz K, Broz D, Agnew-Brune C, Johnson Jones M, Cha S. Factors associated with exchange sex among women and men who inject drugs-23 us cities, 2018. *Sexually Transmitted Infections* 2021; **97(SUPPL 1)**: A90.
173. Curtis M, Barbee L, Glick S, Moreno C. Bacterial sexually transmitted infections among women who inject drugs and exchange sex in king county, Washington. *Sexually Transmitted Infections* 2019; **95**(Supplement 1): A307.
174. Brookmeyer KA, Haderxhanaj LT, Hogben M, Leichliter J. Sexual risk behaviors and STDs among persons who inject drugs: A national study. *Prev Med* 2019; **126**: 105779.
175. Centers for Disease Control and Prevention. HIV Infection, Risk, Prevention, and Testing Behaviors among Persons Who Inject Drugs: National HIV Behavioral Surveillance Injection Drug Use 20 U.S. Cities, 2012. 2015.
176. Garfein RS, Rondinelli A, Barnes RFW, et al. HCV infection prevalence lower than expected among 18-40-year-old injection drug users in San Diego, CA. *Journal of Urban Health* 2013; **90**(3): 516-28.
177. Nerlander LM, Hess KL, Rose CE, et al. Exchange Sex and HIV Infection Among Women Who Inject Drugs-20 US Cities, 2009. *J Acquir Immune Defic Syndr* 2017; **75 Suppl 3**(Suppl 3): S333-s40.
178. Gordon KS, Chiasson MA, Hoover DR, Martins SS, Wilson PA, Lewis CF. Difference in HIV testing behavior by injection status, among users of illicit drugs. *AIDS Care* 2022; **34**(6): 776-83.
179. Zulea WA, Bobashev G. High dead-space syringes and the risk of HIV and HCV infection among injecting drug users. *Drug and Alcohol Dependence* 2009; **100**(3): 204-13.
180. Huo D, Ouellet LJ. Needle exchange and sexual risk behaviors among a cohort of injection drug users in Chicago, Illinois. *Sexually Transmitted Diseases* 2009; **36**(1): 35-40.
181. National AIDS/STD Programme Bangladesh. Mapping Study and Size Estimation of Key Populations in Bangladesh. 2016.
182. National AIDS/STD Program Bangladesh. Behavioral Surveillance Survey 2006-07: Technical Report. 2008.
183. Plan International. Enquete de Surveillance de Deuxieme Generation Relative Aux Ist, Vih et Sida (ESDG) Edition 2017. Akpapkanme Akowéhonto, Benin: Institute for Analysis of Communication and Social Assemblies (INCACES - SARL), 2017.
184. Plan Bénin. Enquête de surveillance de deuxième génération du VIH et des IST auprès des utilisateurs de drogues injectables au Bénin. 2014.
185. Yao Y, Wang N, Chu J, et al. Sexual behavior and risks for HIV infection and transmission among male injecting drug users in Yunnan, China. *International Journal of Infectious Diseases* 2009; **13**(2): 154-61.
186. National AIDS Control Organization (NACO). India: National Integrated Biological and Behavioural Surveillance (IBBS) 2014-15. 2015.
187. Ganesh B, Mosoniro K, Vasna J, Elangovan A, Santhakumar A, Shobini R. Factors associated with human immunodeficiency virus infection and self-assessed risk to human immunodeficiency virus among injecting drug users in Manipur, India. *Indian J Public Health* 2020; **64**(Supplement): S61-s6.
188. Armstrong G, Humtsoe C, Kermode M. HIV risk behaviours among injecting drug users in Northeast India following scale-up of a targeted HIV prevention programme. *BMC Public Health* 2011; **11 Suppl 6**: S9.
189. Wisaksana R, Rusyda Hinduan Z. Integrated Biological-Behavioral Surveillance Survey Among Adolescent and Young People Who Inject Drugs, Female Sex Workers, Males Who Have Sex With Males and Male to Female Transgender Persons: Bandung, Indonesia 2018-2019. 2019.
190. Stoicescu C, Ameilia R, Irwanto, Praptoraharjo I, Mahanani M. Syndemic and Synergistic Effects of Intimate Partner Violence, Crystal Methamphetamine, and Depression on HIV Sexual Risk Behaviors among Women Who Inject Drugs in Indonesia. *J Urban Health* 2019; **96**(3): 477-96.
191. Roshanfekr P, Karimi SE, Narouee S, et al. Life-time HIV testing among people who inject drugs in Iran: results from the National Rapid Assessment and Response survey. *Front Public Health* 2023; **11**: 1253407.

192. Bhattacharjee P, McClarty LM, Musyoki H, et al. Monitoring HIV prevention programme outcomes among key populations in Kenya: Findings from a national survey. *PLoS ONE* 2015; **10 (8) (no pagination)**(e0137007).
193. Syvertsen JL, Agot K, Ohaga S, et al. Evidence of injection drug use in Kisumu, Kenya: Implications for HIV prevention. *Drug and Alcohol Dependence* 2015; **151**: 262-6.
194. Mirzoyan L, Berendes S, Jeffery C, et al. New evidence on the HIV epidemic in Libya: Why countries must implement prevention programs among people who inject drugs. *JAIDS Journal of Acquired Immune Deficiency Syndromes* 2013; **62**(5): 577-83.
195. National AIDS Program Myanmar. Myanmar Integrated Biological and Behavioral Surveillance Survey of People Who Inject Drugs, 2014.
196. WHO. HIV Bio-Behavioral Survey among Injecting Drug Users in the East Jerusalem Governorate. 2010.
197. Ayyaz Z, Dolan K. Narcotics are cheaper than food in Pakistan: Our PWIDs are dying from AIDS. *Sexually Transmitted Infections* 2017; **93**(Supplement 2): A231-A2.
198. Scheibe A, Makapela D, Brown B, et al. HIV prevalence and risk among people who inject drugs in five South African cities. *International Journal of Drug Policy* 2016; **30**: 107-15.
199. The Global Fund. The Integrated Bio-Behavioral Survey (IBBS) in Syria: 2013-2014. 2014.
200. Kpelly DEE, Bernoussi A, Masson J, Schauder S, Kokou-Kpolou K. The estimate of risks to users of injectable drugs in Togo. *Psychotropes (Belgium)* 2018; **24**(1): 77-92.
201. Folch C, Casabona J, Espelt A, et al. Gender differences in HIV risk behaviours among intravenous drug users in Catalonia, Spain. *Gaceta Sanitaria* 2013; **27**(4): 338-43.
202. Centers for Disease Control and Prevention. HIV Infection Risk, Prevention, and Testing Behaviors Among Persons Who Inject Drugs National HIV Behavioral Surveillance, 20 U.S. Cities, 2022, 2022.
203. Marks LR, Durkin MJ, Ayres K, Ellis M. Drug preparation, injection-related infections, and harm reduction practices among a national sample of individuals entering treatment for opioid use disorder. *Harm Reduct J* 2024; **21**(1): 16.
204. Sazonova Y, Kulchynska R, Sereda Y, et al. HIV treatment cascade among people who inject drugs in Ukraine. *PLoS One* 2020; **15**(12): e0244572.
